# Heme cytotoxicity is the consequence of endoplasmic reticulum stress in atherosclerotic plaque progression

**DOI:** 10.1038/s41598-021-89713-3

**Published:** 2021-05-17

**Authors:** Dávid Pethő, Zoltán Hendrik, Annamária Nagy, Lívia Beke, Andreas Patsalos, László Nagy, Szilárd Póliska, Gábor Méhes, Csaba Tóth, László Potor, John W. Eaton, Harry S. Jacob, György Balla, József Balla, Tamás Gáll

**Affiliations:** 1 Division of Nephrology, Department of Internal Medicine, Faculty of Medicine, University of Debrecen, Debrecen, Hungary; 2 Kálmán Laki Doctoral School, Faculty of Medicine, University of Debrecen, Debrecen, Hungary; 3 Department of Pathology, Faculty of Medicine, University of Debrecen, Debrecen, Hungary; 4 Department of Biochemistry and Molecular Biology, Faculty of Medicine, University of Debrecen, Debrecen, Hungary; 5 Departments of Medicine and Biological Chemistry, Johns Hopkins University School of Medicine and All Children’s Hospital, St. Petersburg, FL USA; 6 Proteomics Core Facility, Department of Biochemistry and Molecular Biology, Faculty of Medicine, University of Debrecen, Debrecen, Hungary; 7 Division of Vascular Surgery, Department of Surgery, Faculty of Medicine, University of Debrecen, Debrecen, Hungary; 8 HAS-UD Vascular Biology and Myocardial Pathophysiology Research Group, Hungarian Academy of Sciences, University of Debrecen, Debrecen, Hungary; 9 James Graham Brown Cancer Center, University of Louisville, Louisville, KY USA; 10 Department of Medicine, University of Minnesota, Minneapolis, MN USA; 11 Department of Pediatrics, Faculty of Medicine, University of Debrecen, Debrecen, Hungary

**Keywords:** Cell biology, Medical research

## Abstract

Hemorrhage and hemolysis with subsequent heme release are implicated in many pathologies. Endothelial cells (ECs) encounter large amount of free heme after hemolysis and are at risk of damage from exogenous heme. Here we show that hemorrhage aggravates endoplasmic reticulum (ER) stress in human carotid artery plaques compared to healthy controls or atheromas without hemorrhage as demonstrated by RNA sequencing and immunohistochemistry. In EC cultures, heme also induces ER stress. In contrast, if cultured ECs are pulsed with heme arginate, cells become resistant to heme-induced ER (HIER) stress that is associated with heme oxygenase-1 (HO-1) and ferritin induction. Knocking down HO-1, HO-2, biliverdin reductase, and ferritin show that HO-1 is the ultimate cytoprotectant in acute HIER stress. Carbon monoxide-releasing molecules (CORMs) but not bilirubin protects cultured ECs from HIER stress via HO-1 induction, at least in part. Knocking down HO-1 aggravates heme-induced cell death that cannot be counterbalanced with any known cell death inhibitors. We conclude that endothelium and perhaps other cell types can be protected from HIER stress by induction of HO-1, and heme-induced cell death occurs via HIER stress that is potentially involved in the pathogenesis of diverse pathologies with hemolysis and hemorrhage including atherosclerosis.

## Introduction

The rupture of red blood cells followed by hemoglobin (Hb) and subsequent heme release is a hallmark of various human pathologies such as organ/tissue injuries, malaria, brain hemorrhages, rhabdomyolysis with kidney failure, atherosclerosis with ruptured plaques, complications of surgery or blood transfusion, inherited hemolytic syndromes, sickle cell disease, sepsis resulting in high free heme concentration in the plasma or the extracellular space^1–3^. Although heme is essential for oxygen- and electron transport systems as the prosthetic group of hemoproteins such as hemoglobin, myoglobin, and cytochromes, free heme is toxic to cells via its pro-oxidant, pro-inflammatory, and cytotoxic effects^4,5^. From an evolutionary perspective, intracellular free heme is strictly controlled not only in eukaryotes^6^, but also in bacteria^7^. In mammals, multiple defense mechanisms have been evolved to neutralize free heme by scavenging extracellular heme by specific heme-binding proteins such as hemopexin^8^ and alpha-1-microglobulin^9^, or by catabolizing intracellular labile heme by heme oxygenase-1 (HO-1) breaking it into carbon monoxide, iron, and biliverdin (BV), the latter being converted to bilirubin (BR). Importantly, not only heme but also its end-products are carefully catabolized. BR neurotoxicity, especially in newborns, is well documented^10^. Free iron, in the presence of reactive oxygen species, damages biological systems via the Fenton-reaction^11^ that is inhibited by specific iron-binding proteins with ferroxidase activity, such as ceruloplasmin and the evolutionarily ancient protein ferritin. Carbon monoxide (CO) and BR also possess remarkable antioxidant-, anti-inflammatory- and anti-apoptotic effects^12,13^.

ER stress is involved in diverse human pathologies such as Alzheimer's disease, Parkinson's disease, and bipolar disorder^14^, diabetes, neurodegeneration, and cancer^15^, and atherosclerosis^16^. Earlier, we found that heme, abundantly released during hemolysis and hemorrhage, triggers ER stress in human aortic smooth muscle cells^17^. Given that endothelial cells (ECs) are in direct contact with the bloodstream, ECs are the first innocent bystanders of hemolysis- and hemorrhage-driven heme toxicity. This prompted us to examine whether heme triggers ER stress in ECs and whether heme-induced ER (HIER) stress is present in hemorrhaged atherosclerotic plaques during disease progression. As HO-1, the only inducible heme metabolizing enzyme of eukaryotic cells targeting intracellular free heme, anchors to the cytoplasmic surface of the ER, we speculated that this localization might have significance in heme-driven toxicity.

Here, we used multiple approaches to identify how HIER stress might contribute to the pathogenesis of hemolytic/hemorrhagic diseases, and to provide evidence of how HIER stress might lead to cellular injury. RNA sequencing and immunohistochemistry showed that hemorrhage aggravated ER stress in complicated human atherosclerotic lesions. Activation of the HO-1/ferritin system by the non-toxic heme alternative, heme arginate, provided tolerance to HIER stress in ECs. Knocking down HO-1 but not ferritin heavy chain and biliverdin reductase aggravated HIER stress. We showed that HO-1 dramatically lessens cytotoxicity but that currently known cell death inhibitors had no effect. We also found that heme catabolism by-product CO but not BR attenuated HIER stress. Overall, we demonstrate that HIER stress is, at least partly, involved in the pathogenesis of hemolytic/hemorrhagic human pathologies, and heme arginate and CO releasing molecules might have therapeutic potential in these pathologies.

## Results

### Plaque hemorrhage increases ER stress in atherosclerotic patients

**Figure 1:**
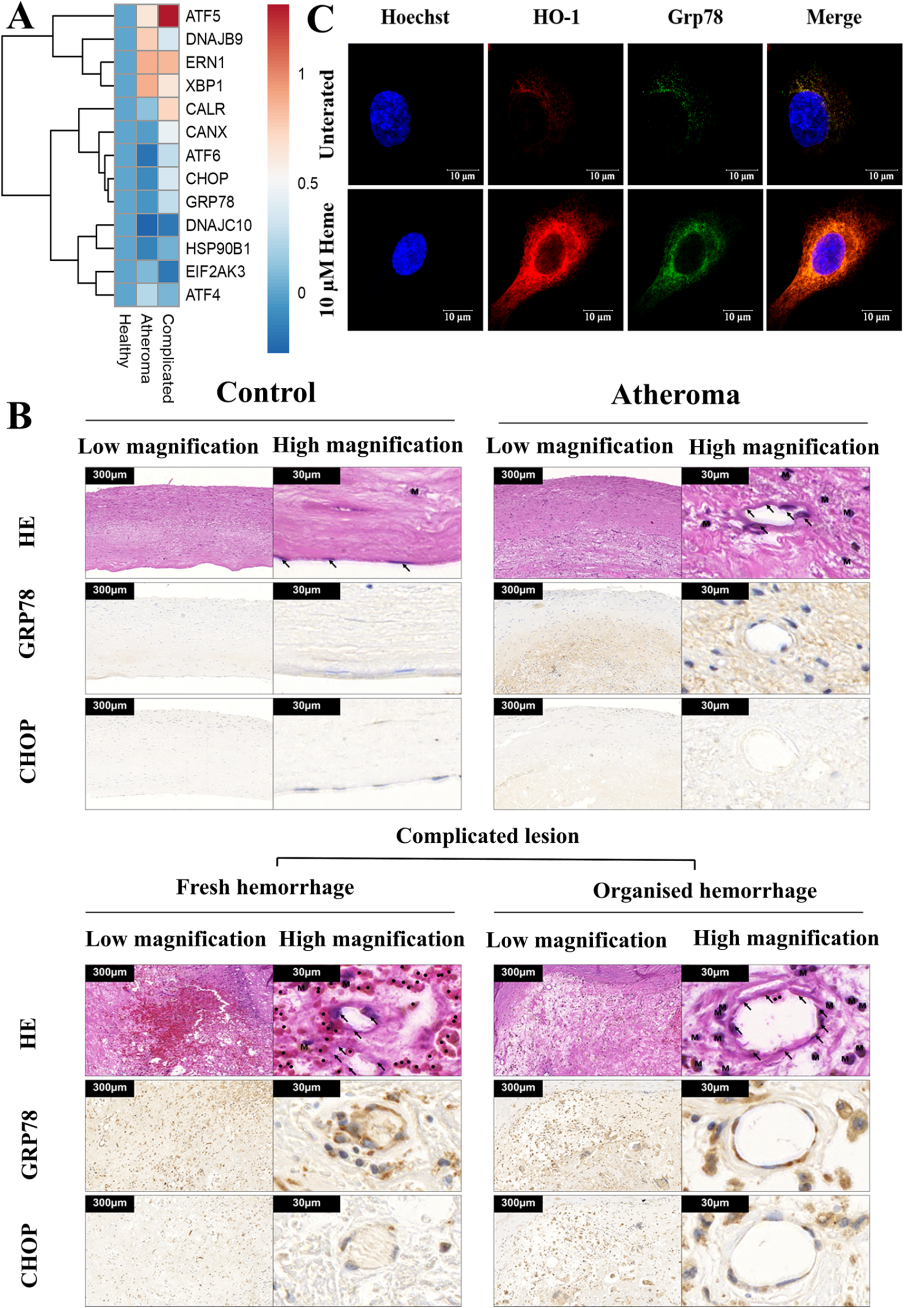
Hemorrhage and heme induces endoplasmic reticulum (ER) stress in human carotid artery plaques and endothelial cell (EC) cultures. (**A**) RNA sequencing analysis of ER stress marker expression in healthy carotid arteries, atheromas, and complicated plaques with hemorrhage. To obtain global transcriptome data of human carotid arteries biopsies from patients (n = 5) with atherosclerotic lesions, high throughput mRNA sequencing analysis was performed and log(Fold Changes) are shown compared to the healthy samples. Raw sequencing data (fastq) was aligned to human reference genome version GRCh37 using HISAT2 algorithm and BAM files were generated. Downstream data analysis was performed using Strand NGS software, Version 3.4, Build 230243 (Strand Life Sciences, Bangalore, India; https://www.strand-ngs.com/). BAM files were imported into the software and DESeq algorithm was used for normalization. To identify differentially expressed genes between atherosclerotic lesions and healthy biopsies, ANOVA with Tukey post hoc test was used. Heatmaps were drawn using R packages pheatmap (R Core Team (2020). R: A language and environment for statistical computing. R Foundation for Statistical Computing, Vienna, Austria. URL http://www.R-project.org/ and ggplot2^72^. (**B**) Expression of ER chaperone Grp78 and death signal protein CHOP in healthy carotid wall, atheromatous plaque, and complicated lesion with fresh or organized hemorrhage. Immunomorphological features associated with different stages of the atherosclerotic process: serial sections of healthy carotid wall (upper panel, left), atheromatous plaque (upper panel, right), complicated lesion with fresh (lower panel, left), or organized hemorrhage (lower panel, right) are shown. H&E staining, CHOP, and Grp78 immunohistochemistry are presented from the same tissue areas, all pictures and all inserts with the same magnification. (**C**) EC cultures were exposed to heme (10 μM) for 2 h in serum-free CM199, then the medium was changed to CM199 with 10% FCS and antibiotics. Grp78 and HO-1 expression were analyzed with immunofluorescence using high-resolution microscopy after 16 h.

During atherosclerotic plaque progression, hemorrhage frequently occurs due to plaque rupture or blood leakage from the immature neovasculature. To assess how ER stress marker expression changes in response to hemorrhage, we performed RNA-seq analysis of healthy control carotid arteries, atheromas without hemorrhage, and complicated hemorrhaged lesions. Genes involved in ER stress are depicted in the clustered heatmap in Fig. 1A and summarized in Table 1. ER stress gene expressions were markedly changed during plaque progression. The difference in expression was considerable between the atheroma and the complicated, hemorrhaged group with a marked increase in several genes (ATF5, CALR, CANX, ATF6, CHOP, GRP78, HSP90B1), while others decreased in response to hemorrhage (DNAJB9, XBP1, EIF2AK3, ATF4). These results indicate that, in humans, the ER stress gene expression pattern is being dynamically changed during atherogenesis, and hemorrhage markedly modulates the ER stress response.

**Table 1:** The role of ER stress responsive proteins in atherosclerosis.

Gene		Function/role in atherosclerosis	References
ATF5	Activating transcription factor 5	Induced by CHOP triggering a vaiety of cellular responses including cell death	^55^
DNAJB9	DnaJ heat shock protein family (Hsp40) member B9	Implicated in ER-associated degradation (ERAD) of multiple unfolded secretory proteins	^27^
ERN1	Endoplasmic reticulum to nucleus signaling 1	A proxiamal sensor of ER stress splicing XBP1; targeting ERN1 (IRE1) counteracts with atherosclerosis progression	^56,57^
XBP1	X-box-binding protein 1	Sustained XBP1 splicing activation leads to endothelial apoptosis and atherosclerosis development in response to disturbed flow	^58^
CALR	Calreticulin	Regulation of intracellular Ca^2+^ homoeostasis and ER Ca^2+^ capacity, ER chaperone	^59^
CANX	Calnexin	ER chaperone	^60^
ATF6	Activating transcription factor 6	Activates transcription of ER chaperones and components of ER-associated degradation, elevated expression of active ATF6 aggravates endoplasmic reticulum stressinduced vascular endothelial cell apoptosis	^61^
DDIT3 (CHOP)	DNA damage inducible transcript 3	Involved in ER stress-dependent cell death in many pathologies among them atherosclerosis	^62^
HSPA5 (GRP78)	Heat shock protein family A (Hsp70) member 5	Molecular chaperone of ER; induced by atherogenic stimuli	^63,64^
DNAJC10	DnaJ heat shock protein family (Hsp40) member C10	ER disulfide reductase associated both with the correct protein folding and degradation of misfolded proteins	^65^
HSP90B1	Heat shock protein 90 beta family member 1	ER chaperone; also induced in atherosclerosis	^66,67^
EIF2AK3	Eukaryotic translation initiation factor 2 alpha kinase 3	Is activated by autophosphorylation, attenuates protein translation in response to ER stress	^68^
ATF4	Activating transcription factor 4	Increases the expression of genes essential for the recovery from ER stress	^69^

Glucose-regulated protein 78 (GRP78) is a major ER chaperone protein crucial in protein quality control of the ER, which also controls the activation of the ER-transmembrane signaling proteins PERK, IRE1, and ATF6^18^. ER stress increases the level of C/EBP-homologous protein (CHOP) that mediates cell death^19^. As ECs primarily regulate vascular functions, we wondered whether hemorrhage modulates CHOP and Grp78 expressions in ECs during plaque progression in human carotid artery samples (Fig. 1B). We found that ECs both in fresh and organized hemorrhaged plaques accumulated high levels of CHOP and Grp78 compared to atheromas without hemorrhage or healthy controls. Notably, macrophages also showed significant ER stress in hemorrhaged plaques. Since hemorrhaged plaques are characterized by a significant amount of free heme^17^, we next investigated whether heme triggers ER stress in ECs in vitro. We showed that heme also induces ER stress in cultured EC (Fig. 1C) suggesting that elevated ER stress in ECs might be associated with hemorrhage.

### Heme induces ER stress in ECs

After establishing the clinical relevance of hemorrhage-induced ER stress in atherosclerosis, we next analyzed whether heme induces ER stress in EC cultures. In hemolytic conditions, high concentrations of heme (20–50 µM) can be achieved in the plasma^1^. Total plasma heme concentration also raises after RBC transfusion and predicts mortality in critically ill patients reaching high level of heme (54 [35–136] µM)^20^. In murine hemolytic model, plasma heme concentration increases to 120 µM from a steady level of 23 µM detected at basal condition^21^. Heme content of hemorrhaged atherosclerotic plaques is also fourfold compared to healthy carotid arteries reaching as high as 100 µmol/mg protein^17^.

As activating transcription factor-5 (ATF-5) as well as Endoplasmic Reticulum To Nucleus Signaling 1 (ERN1) mRNAs were highly elevated in hemorrhaged atherosclerotic plaques, we first analyzed whether these genes are also induced by heme in ECs in vitro. We showed that heme induced ATF-5 expression neither at mRNA nor at protein level (Supplementary Figure 1). Interestingly, ERN1 mRNA was dose-dependently induced by heme at mRNA level (about twofold), however, this induction was naot evident at protein level (Supplementary Figure 2). This prompted us to examine in vitro other ER stress genes that also showed considerable change in RNAseq, namely CHOP and Grp78.

**Figure 2:**
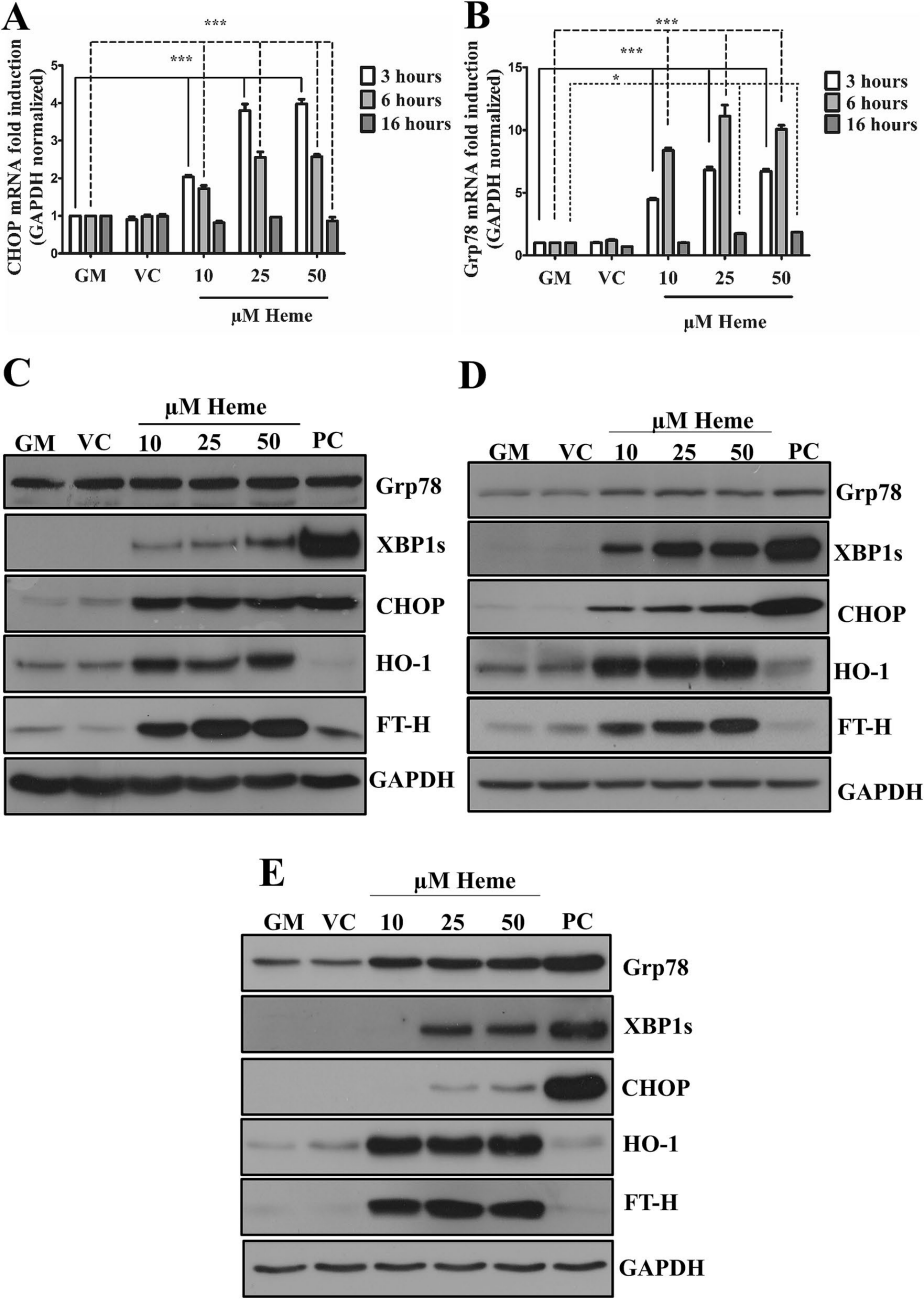
Heme activates ER stress in EC cultures in a time- and dose-dependent manner. ECs were treated with various doses of heme (10, 25, and 50 μM) or corresponding vehicle solution to the highest heme dose (50 μM) in serum- and antibiotics-free CM199 for 2 h, then the medium was changed to CM199 with 10% FCS and antibiotics. Thapsigargin (1 μM) treated cells were used as positive control. (**A**) Relative expression of CHOP and (**B**) Grp78 mRNA were analyzed after 3, 6, or 16 h and normalized to GAPDH. Representative immunoblots of three independent experiments are shown representing Grp78, spliced XBP1, CHOP, HO-1, and ferritin heavy chain levels (**C**) 3, (**D**) 6, and (**E**) 16 h after the heme treatments. *GM* growth medium, *VC* vehicle control, *PC* positive control. Data are shown as mean ± SEM of three independent experiments. Immunoblots are cropped from different parts of the same gel. Uncropped immunoblots are presented in the Supplementary information. Statistical analysis was performed by one-way ANOVA test followed by Bonferroni correction. A value of *p* < 0.05 was considered significant. * p < 0.05, ***p < 0.001.

Consistent with our RNA seq and immunohistochemistry data, we found that as low as 10 µM of heme triggered the expression of ER stress marker CHOP and Grp78 as early as 3 h, and catalyzed the expression of the spliced XBP1 (XBP1s) in a time- and dose-dependent manner (Fig. 2A–E). As expected, heme also triggered HO-1 and ferritin heavy chain (FT-H) expressions (Fig. 2C–E). This highlights the relevance of ER stress in heme-driven EC damage.

**Figure 3:**
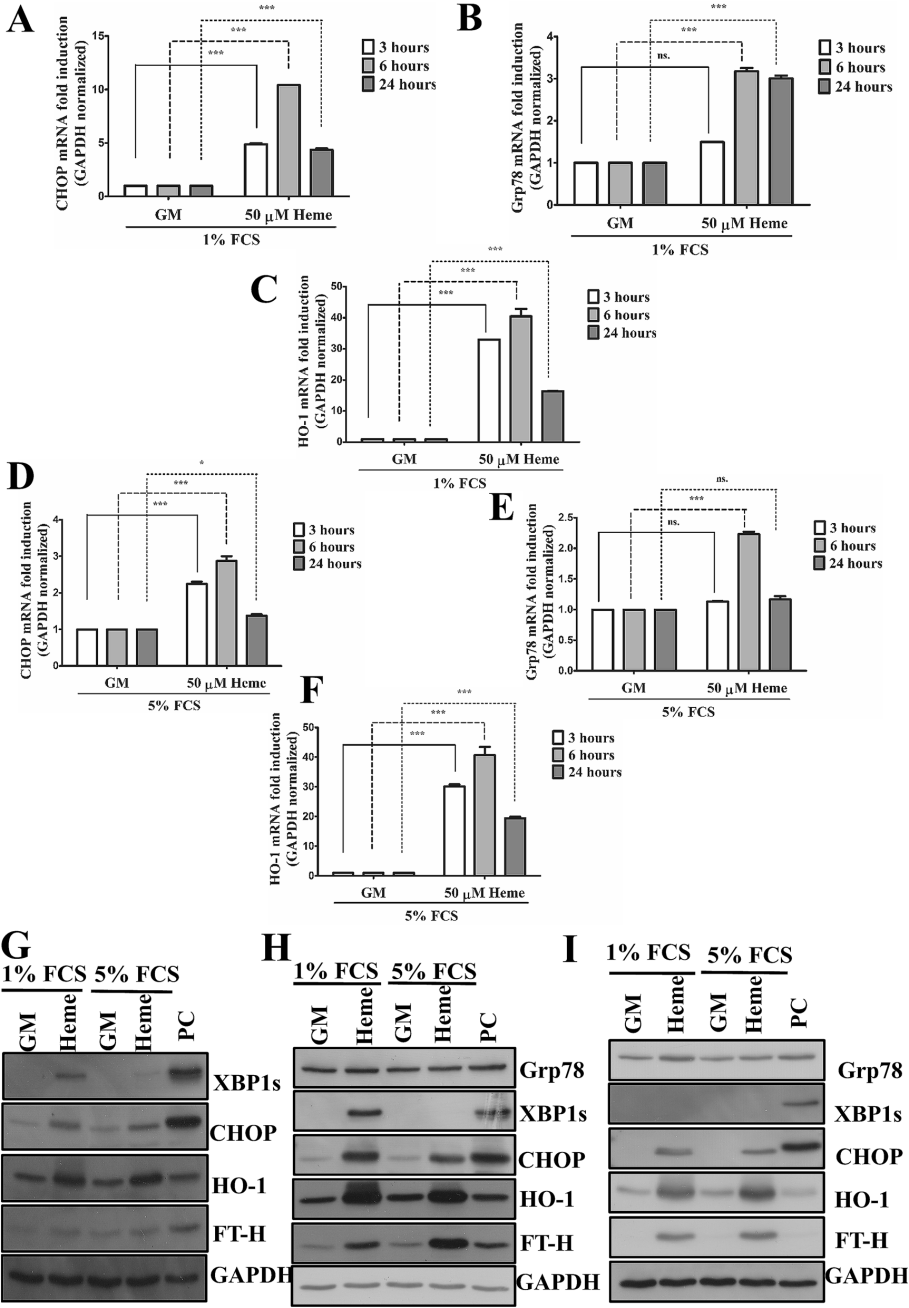
Heme activates ER stress in EC cultures in the presence of serum. ECs were incubated with heme (50 µM) in presence of FCS (1 and 5%) for 3, 6, and 24 h. Thapsigargin (1 μM) treated cells were used as positive control. (**A**) Relative expression of CHOP (DDIT3), (**B**) Grp78 and HO-1, (**C**) mRNA in culture medium containing 1% FCS were analyzed after 3, 6 or 24 h and normalized to GAPDH. (**D**) Relative expression of CHOP (DDIT3), (**E**) Grp78 and HO-1 (**F**) mRNA in culture medium containing 5% FCS were analyzed after 3, 6 or 24 h and normalized to GAPDH. Representative immunoblots of three independent experiments are shown representing Grp78, spliced XBP1, CHOP, HO-1, and ferritin heavy chain levels after (**G**) 3, (**H**) 6, and (**I**) 24 h. *GM* growth medium, *VC* vehicle control, *PC* positive control. Data are shown as mean ± SEM of three independent experiments. Immunoblots are cropped from different parts of the same gel. Uncropped immunoblots are presented in the Supplementary information. Statistical analysis was performed by one-way ANOVA test followed by Bonferroni correction. A value of *p* < 0.05 was considered significant. * p < 0.05, ***p < 0.001; *ns* non-significant.

In another set of experiments, we have tested whether HIER stress is also detectable in ECs when serum components are present. In these experiments, EC were incubated with heme (50 µM) in presence of FCS (1 and 5%) for 3, 6, and 24 h. Our results showed that heme triggers ER stress (Fig. 3A,B,D,E,G,H) in the presence of serum components, however, higher serum concentration decreases ER stress compared to the lower serum level. As expected, heme also induced HO-1 and FT-H expressions (Fig. 3C,F–H). Interestingly, XBP1s activation considerably decreased over time compared to CHOP protein level which was detectable even after 24 h suggesting a sustained CHOP activation in ECs when heme is permanently present in the experimental medium (Fig. 3G–I).

Since GAPDH has been found to be a heme chaperone binding free heme^22^. To exclude that GAPDH gene expression itself is changed under heme stress, we exposed ECs to various doses of heme (10–50 µM) for 2 h in serum- and antibiotics-free CM199 medium followed by a 3-h-incubation in CM199 medium containing 10% FCS and antibiotics, then, a qPCR analysis of a set of housekeeping genes, such as Phosphoglycerate Kinase 1 (PGK1), β-actin, and TATA-binding protein 1 (TBP1)^23^, together with GAPDH were performed. GAPDH mRNA expression was normalized to a set of the above mentioned housekeeping genes We showed GAPDH mRNA expression was not strongly altered by the experimental conditions used (Supplementary Figure 3).

**Figure 4:**
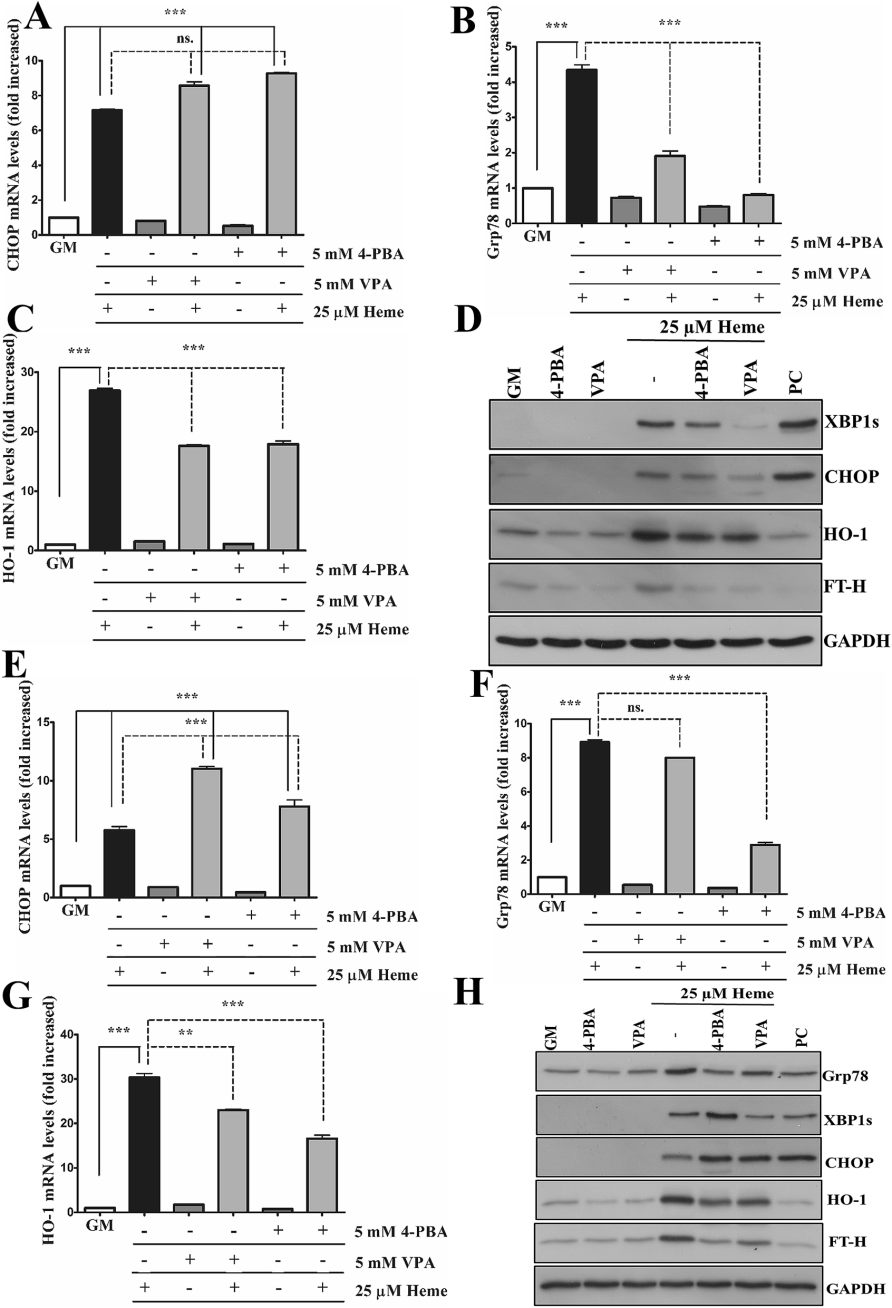
ER stress inhibitors does not protect against HIER stress. ECs were pre-incubated with either 4-phenylbutyric acid (4-PBA) (5 mM) or valproic acid (VPA) (5 mM) overnight, then exposed to heme (25 µM) in serum- and antibiotics-free CM199 supplemented with either 4-PBA (5 mM) or VPA (5 mM), then further incubated with CM199 supplemented with 10% FCS, antibiotics, and either 4-PBA (5 mM) or VPA (5 mM) for 3 h and 6 h. (**A**) Relative expression of CHOP, (**B**) Grp78, and (**C**) HO-1 were analyzed after 3 h and (**E**) CHOP, (**F**) Grp78, (**G**) HO-1 6 h. Representative immunoblots of three independent experiments are shown representing Grp78, spliced XBP1, CHOP, HO-1, and ferritin heavy chain levels after (**D**) 3 and (**H**) 6 h. *GM* growth medium, *VC* vehicle control, *PC* positive control. Data are shown as mean ± SEM of three independent experiments. Immunoblots are cropped from different parts of the same gel. Uncropped immunoblots are presented in the Supplementary information. Statistical analysis was performed by one-way ANOVA test followed by Bonferroni correction. A value of *p* < 0.05 was considered significant. *p < 0.05, *p < 0.01, ***p < 0.001.

4-Phenylbutyric acid (4-PBA) and valproic acid (VPA) are widely used ER stress inhibitors. To investigate whether 4-PBA and VPA inhibit HIER stress, ECs were pre-incubated with either 4-PBA (5 mM) or VPA (5 mM) overnight, then exposed to heme (25 µM) in serum- and antibiotics-free CM199 supplemented with either 4-PBA (5 mM) or VPA (5 mM), then further incubated with CM199 supplemented with 10% FCS, antibiotics, and either 4-PBA (5 mM) or VPA (5 mM) for 3 h and 6 h. Our results showed that neither 4-PBA nor VPA reduced CHOP mRNA expression (Fig. 4A) but reduced Grp78 (Fig. 4B) after 3 h; interestingly, both ER stress inhibitors increased heme-induced CHOP mRNA expression. On the contrary, CHOP expression was not elevated at protein level at this early time point, VPA even reduced heme-induced CHOP protein expression after 3 h (Fig. 4D). In addition, VPA but not 4-PBA markedly reduced XBP1s expression after 3 h (D). Both 4-PBA and VPA lowered heme-induced Grp78 induction after 3 h (Fig. 4C). Importantly, both ER stress inhibitors lowered heme-induced HO-1 and FT-H expression (Fig. 4C,D). After 6 h, both ER stress inhibitors increased CHOP (Fig. 4E,H) but decreased Grp78 (Fig. 4F,H) expression in response to heme. 4-PBA induced XBP1s expression in heme-treated ECs compared to heme alone (Fig. 4H). Similar to 3 h, both 4-PBA and VPA decreased HO-1 (Fig. 4G,H) and FT-H (Fig. 4H) expression in heme-treated cells after 6 h. Overall, these results suggest that both ER stress inhibitors decrease Grp78 expression in heme-treated cells in both time points. VPA is more effective to inhibit XBP1s activation compared to 4-PBA, however, both 4-PBA and VPA aggravated CHOP expression and decreased HO-1/FT-H levels in response to heme.

### Heme arginate ameliorates HIER stress

**Figure 5:**
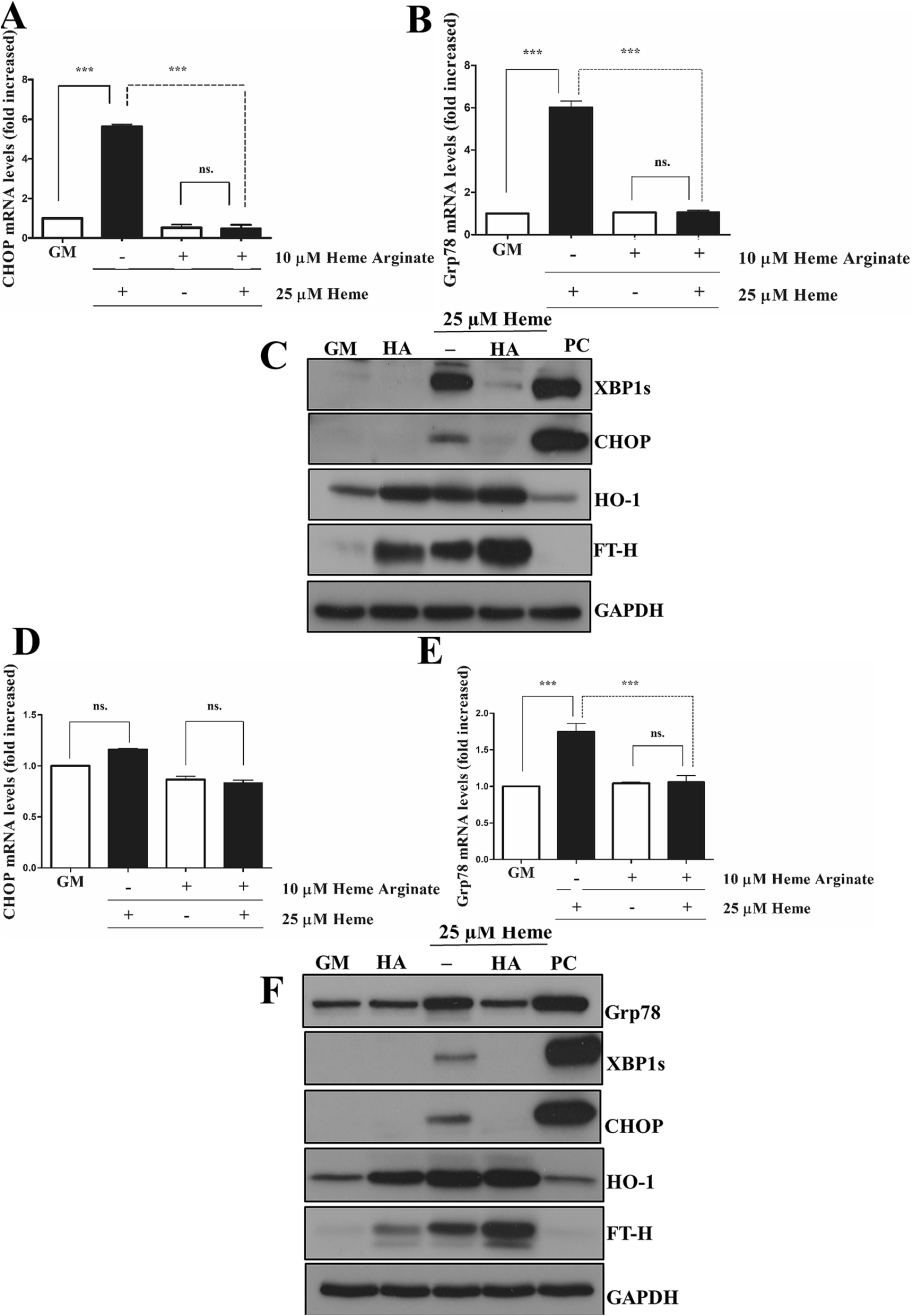
Heme arginate attenuates HIER stress in EC cultures. ECs were treated with 10 µM of HA in CM199 with 10% FCS and antibiotics overnight, then challenged with heme (25 µM) for 2 h in serum- and antibiotics-free CM199. Then, the medium was changed to CM199 with 10% FCS and antibiotics. Relative expression of (**A**) CHOP and (**B**) Grp78 mRNAs as well (**C**) spliced XBP1, CHOP, HO-1, and ferritin heavy chain protein levels were analyzed 3 h after the heme treatment. In another set of experiments, relative expression of (**D**) CHOP and (**E**) Grp78 mRNAs as well (**F**) Grp78, spliced XBP1, CHOP, HO-1, and ferritin heavy chain protein levels were analyzed 16 h after the heme treatment. *GM* growth medium, *HA* heme arginate, *PC* positive control. Relative mRNA expressions were normalized to GAPDH. GAPDH was used as a loading control in immunoblots. Immunoblots are cropped from different parts of the same gel. Uncropped immunoblots are presented in the Supplementary information. Data are shown as mean ± SEM of three independent experiments. Statistical analysis was performed by one-way ANOVA test followed by Bonferroni correction. A value of *p* < 0.05 was considered significant. ***p < 0.001.

We have previously shown that ECs pulsed with a low-dose of heme become more resistant to heme-mediated oxidant injury. To determine whether the induction of HO-1/ferritin before heme stress protects against HIER stress, we pre-treated ECs with a non-toxic alternative of heme, heme-arginate (HA) (10 µM) followed by heme (25 µM) exposure. HA significantly prevented both early (Fig. 5A–C) and late-phase (Fig. 5D–F) HIER stress. HA alone markedly increased HO-1/FTH expression (Fig. 5C,F). This suggests that HA pre-treatment blunts HIER stress, which might be attributed to its HO-1/ferritin-inducing effect.

In another set of experiments, we tested whether low-dose heme could inhibit HIER stress in the same condition as HA which showed that induction of HO-1/FT-H either by HA or low-dose heme also protected against HIER stress provoked by higher heme concentration (Supplementary Figure 4).

### Knocking down HO-1 but not ferritin heavy chain aggravates HIER stress

**Figure 6:**
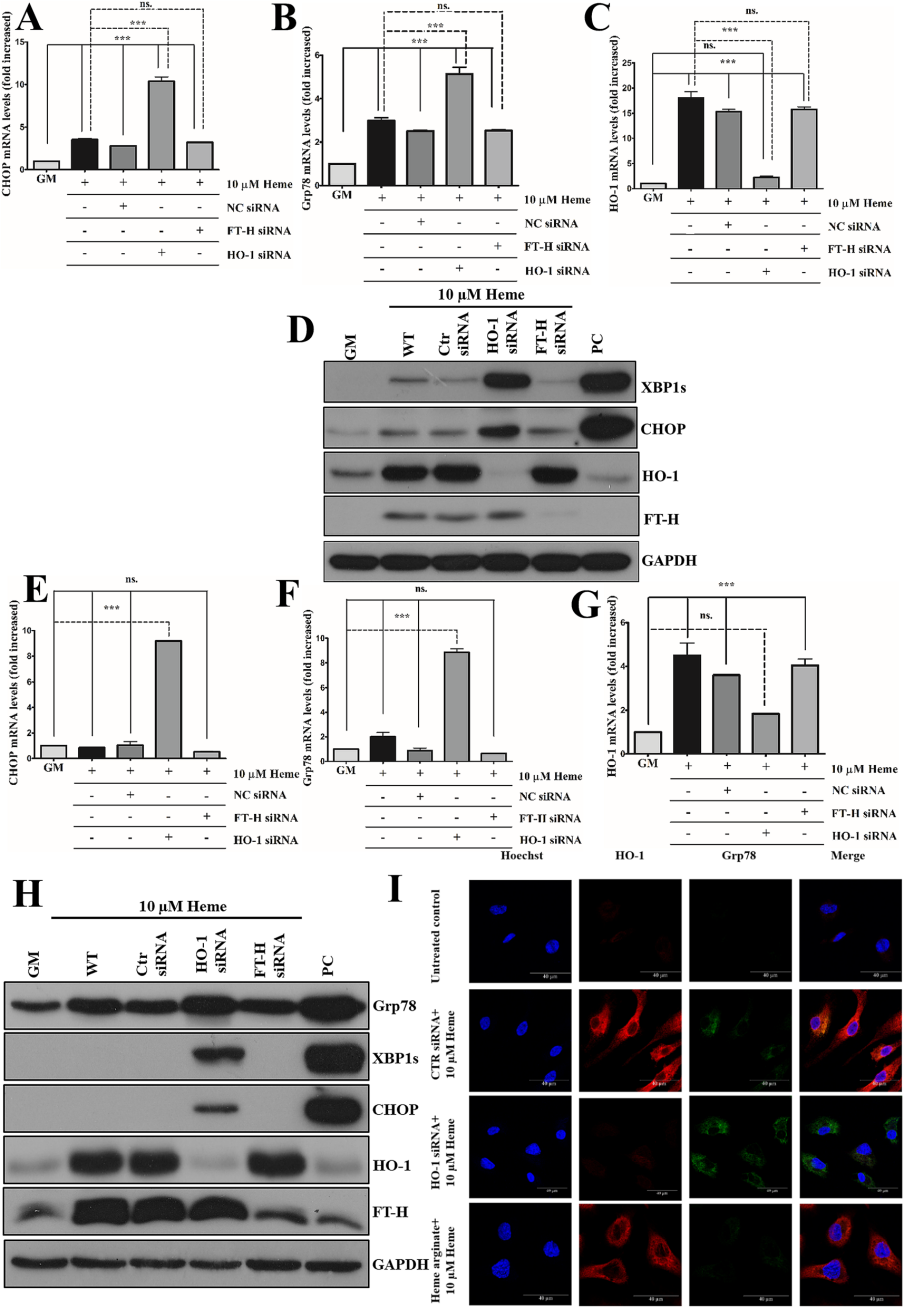
Knocking down HO-1 but not ferritin heavy chain aggravates HIER stress in EC cultures. HO-1 and ferritin heavy chain expressions were knocked down with small interfering RNAs, then ECs were exposed to heme (10 μM) for 2 h in serum- and antibiotics-free CM199. Then, the medium was changed to CM199 with 10% FCS and antibiotics. Relative expression of (**A**) CHOP, (**B**) Grp78, and (**C**) HO-1 mRNAs as well (**D**) spliced XBP1, CHOP, HO-1, and ferritin heavy chain protein levels were analyzed 3 h after the heme treatment. In another set of experiments, relative expression of (**E**) CHOP, (**F**) Grp78, and (**G**) HO-1 mRNAs as well (**H**) Grp78, spliced XBP1, CHOP, HO-1, and ferritin heavy chain protein levels were analyzed 16 h after the heme treatment. (**I**) HO-1 was knocked down or upregulated with heme arginate as described above. ECs were exposed to heme (10 μM) for 2 h in serum- and antibiotics-free CM199. Then, the medium was changed to CM199 with 10% FCS and antibiotics and HO-1 as well as Grp78 expressions were analyzed with immunofluorescence using super-resolution microscopy. *GM* growth medium, *NC* non-coding siRNA, *HA* heme arginate, *PC* positive control. Relative mRNA expressions were normalized to GAPDH. GAPDH was used as a loading control in immunoblots. Immunoblots are cropped from different parts of the same gel. Uncropped immunoblots are presented in the Supplementary information. Data are shown as mean ± SEM of three independent experiments. Statistical analysis was performed by one-way ANOVA test followed by Bonferroni correction. A value of *p* < 0.05 was considered significant. ***p < 0.001.

As HA, presumably by the activation of the HO-1/ferritin system, protected against HIER stress, we next examined how knocking down these key proteins affects HIER stress. Given that ferritin heavy chain (FT-H) protects against heme catalyzed free radical toxicity in various cell types^24–26^, we hypothesized that FT-H might protect ECs against HIER stress. As HO-1 is responsible for catabolism of intracellular heme, HO-1 and FT-H were knocked down in ECs (Fig. 6A–J) followed by heme challenge (10 µM). We found that knocking down HO-1 but not FT-H significantly aggravated HIER stress in the early (Fig. 6B–D) as well as in the late stage (Fig. 6F–H). This effect was more prominent in the late stage where ER stress marker expressions were below the detection limit in wild-type cells but were still elevated when HO-1 was knocked down. Interestingly, marked expression of FT-H was also detected in HO-1-knocked down cells (Fig. 6D,H). Induction of HO-1 by HA inhibited, while knocking down HO-1 dramatically aggravated Grp78 expression (Fig. 6I). This suggests that FT-H expression in ECs does not affect the extent of HIER stress that is likely dependent on HO-1 expression.

### Lack of protection by HA in HO-1-knocked down cells

**Figure 7:**
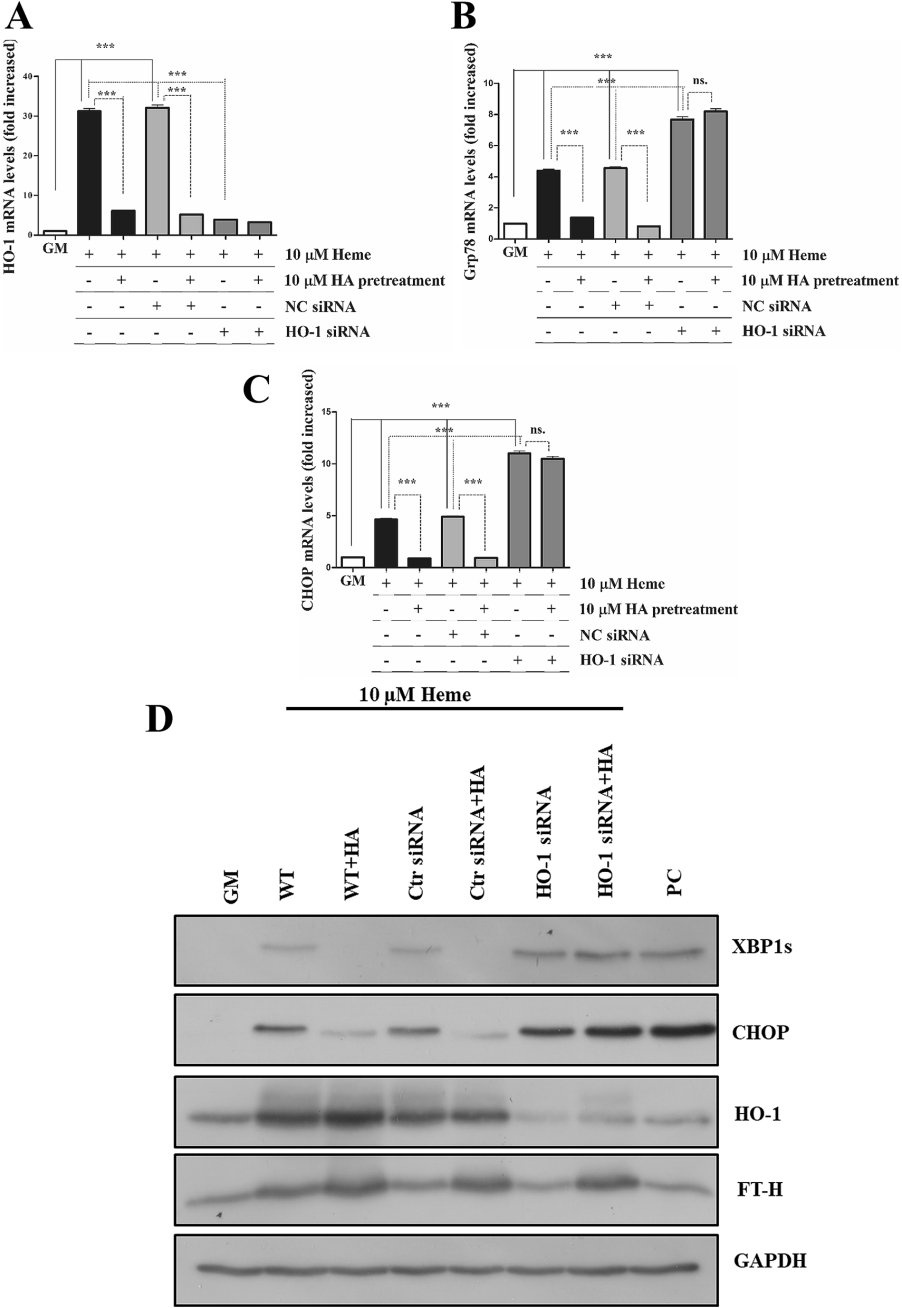
Lack of protection by heme arginate against HIER stress in HO-1-silenced cells. HO-1 expression was knocked down by siRNA antiHO-1 as described above, then ECs were pre-incubated with HA (10 µM) in CM199 medium supplemented with 10% FCS and antibiotics for 16 h. Then, ECs were washed and exposed to heme (10 µM) for 2 h in CM199 medium without FCS and antibiotics. ECs were then washed with HBSS+ and further incubated with CM199 supplemented with 10% FCS and antibiotics for 3 h. (**A**) Relative expression of HO-1, (**B**) Grp78, (**C**) CHOP were analyzed and normalized to GAPDH. (**D**) Representative immunoblot showing XBP1s, CHOP, HO-1, and FT-H expression. *GM* growth medium, *NC* non-coding siRNA, *HA* heme arginate, *PC* positive control. Relative mRNA expressions were normalized to GAPDH. GAPDH was used as a loading control in immunoblots. Immunoblots are cropped from different parts of the same gel. Uncropped immunoblots are presented in the Supplementary information. Data are shown as mean ± SEM of three independent experiments. Statistical analysis was performed by one-way ANOVA test followed by Bonferroni correction. A value of *p* < 0.05 was considered significant. ***p < 0.001.

To test whether HO-1 is the decisive downstream effector of HA-mediated protection against HIER stress, we exposed HO-1-silenced cells to HA as described above then challenged cells with heme (10 µM) as described. We showed the lack of protection by HA in HO-1-knocked down cells suggesting that HO-1 is the decisive downstream effector of HA (Fig. 7A–D).

### Knocking down heme oxygenase-2 (HO-2) does not aggravate HIER stress

**Figure 8:**
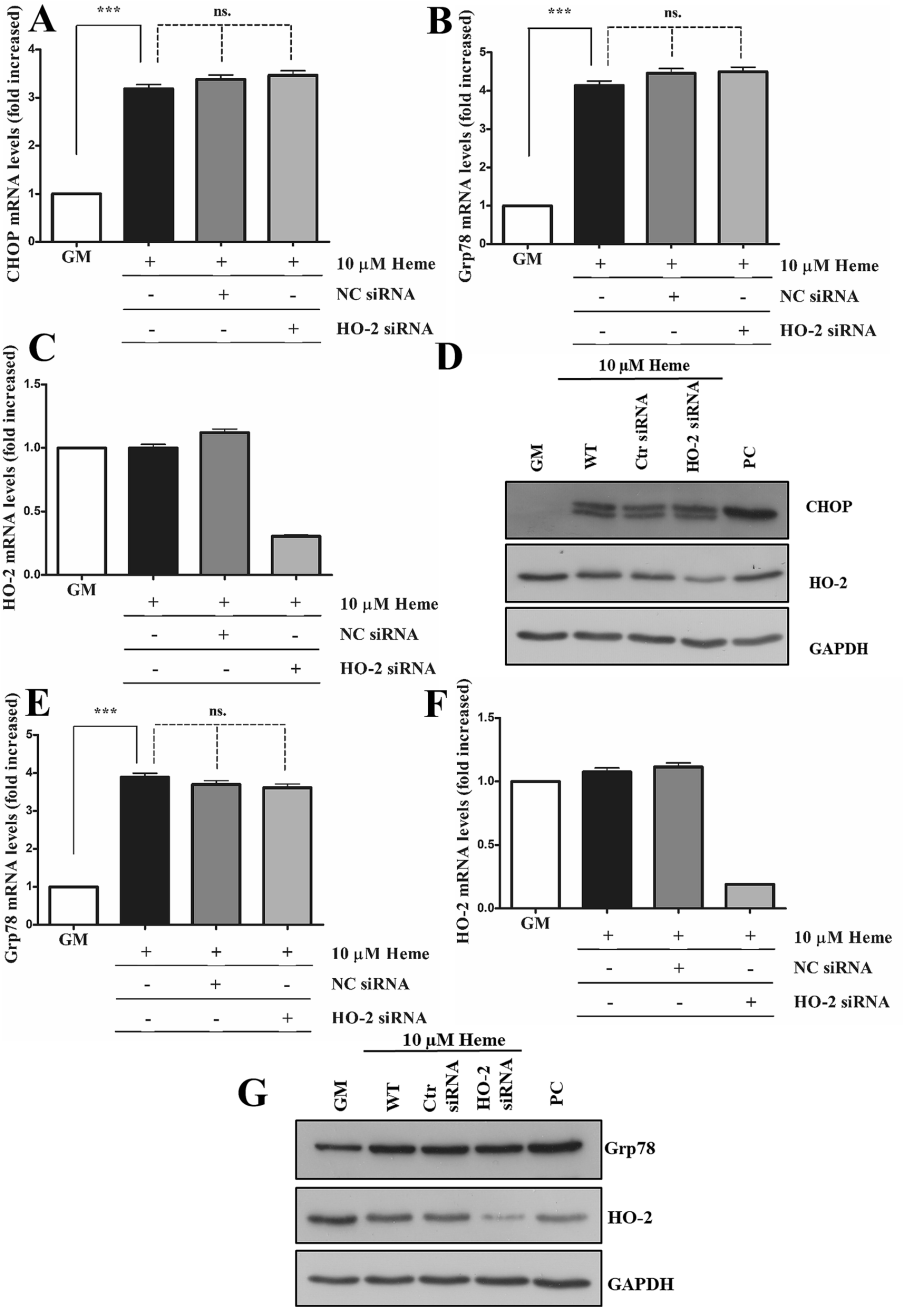
Knocking down heme oxygenase-2 (HO-2) does not aggravate HIER stress. HO-2 expression was knocked down with small interfering RNA, then ECs were exposed to heme (10 μM) for 2 h in serum- and antibiotics-free CM199. Then, the medium was changed to CM199 with 10% FCS and antibiotics. Relative expression of (**A**) CHOP, (**B**) Grp78, and (**C**) HO-2 mRNAs as well (**D**) CHOP and HO-2 protein levels were analyzed 3 h after the heme treatment. In another set of experiments, relative expression of (**E**) Grp78 and (**F**) HO-2 mRNA and (**G**) protein levels were analyzed 16 h after the heme treatment. *GM* growth medium, *NC* non-coding siRNA, *HA* heme arginate, *PC* positive control. Relative mRNA expressions were normalized to GAPDH. GAPDH was used as a loading control in immunoblots. Immunoblots are cropped from different parts of the same gel. Uncropped immunoblots are presented in the Supplementary information. Data are shown as mean ± SEM of three independent experiments. Statistical analysis was performed by one-way ANOVA test followed by Bonferroni correction. A value of *p* < 0.05 was considered significant. ***p < 0.001.

The other known HO isoform is heme oxygenase-2 (HO-2) considered as the constitutive isoform having similar catalytic mechanism and efficiency ^27^. This raises the interesting notion that HO-2 could serve as an effective stopgap as HO-1 is being induced to provide defense against HIER stress in ECs. Therefore, we next tested how knocking down HO-2 could influence HIER stress in ECs. We showed that siRNA-mediated knocking down of HO-2 had no effect on boosting HIER stress in ECs, since HO-2 silencing did not influence ER stress marker expression either at early (3 h) (Fig. 8A–D) or at late (16 h) (Fig. 8E–G) time points after heme exposure.

### Knocking down BVR does not aggravate HIER stress

**Figure 9:**
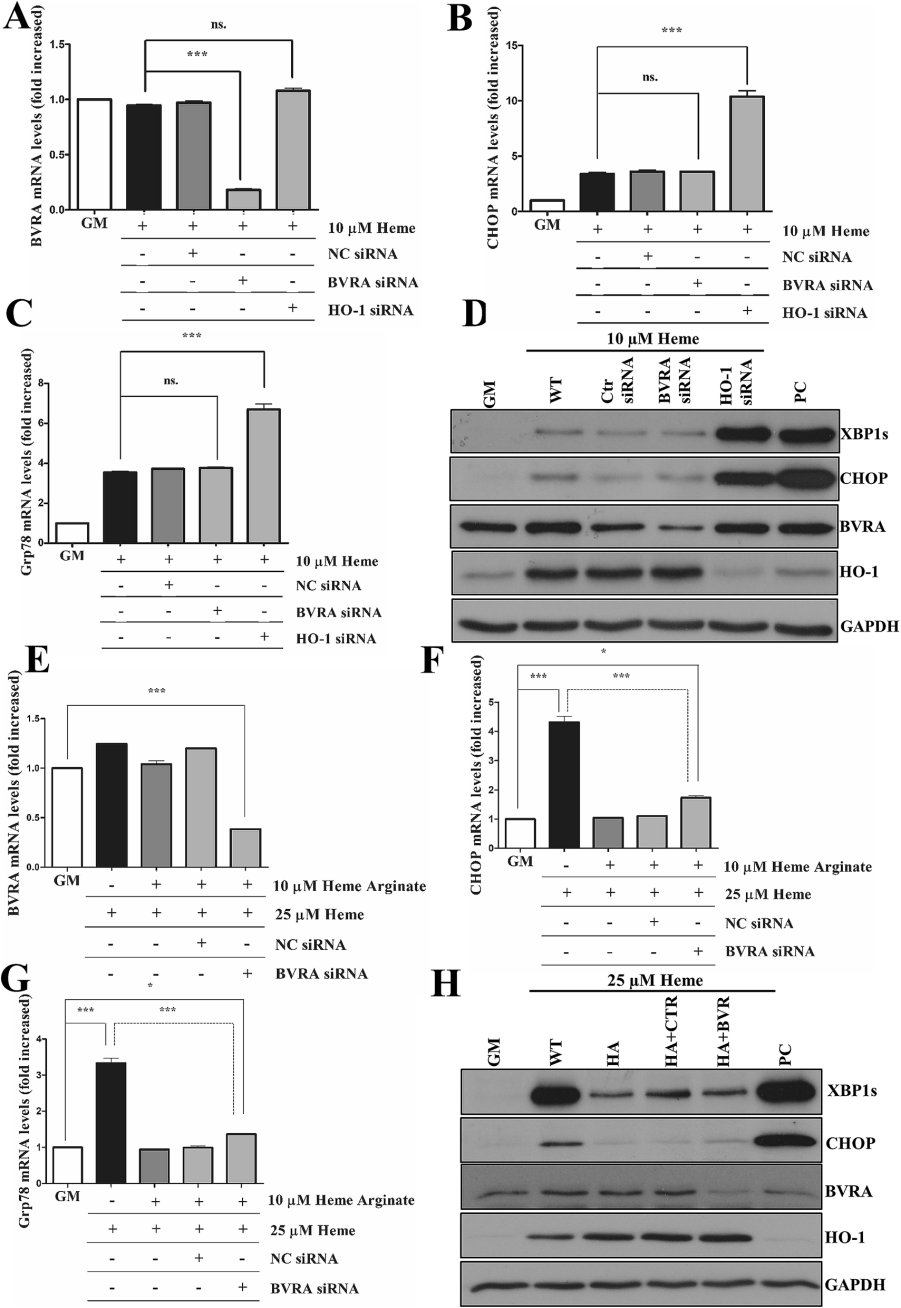
Knocking down biliverdin reductase does not aggravate HIER stress in EC cultures. HO-1 and biliverdin reductase A expressions were knocked down with small interfering RNAs, then ECs were exposed to heme (10 μM) for 2 h in serum- and antibiotics-free CM199. Then, the medium was changed to CM199 with 10% FCS and antibiotics. Relative expression of (**A**) BVRA, (**B**) CHOP, and (**C**) Grp78 mRNAs as well (**D**) spliced XBP1, CHOP, HO-1, and BVRA protein levels were analyzed 3 h after the heme treatment. In another set of experiments, BVRA was knocked down, then, ECs were treated with heme arginate as described above. Then, ECs were exposed to heme (25 μM) for 2 h in serum- and antibiotics-free CM199. Then, the medium was changed to CM199 with 10% FCS and antibiotics. Relative expression of (**E**) BVRA, (**F**) CHOP, and (**G**) Grp78 mRNAs as well (**H**) spliced XBP1, CHOP, HO-1, and BVRA protein levels were analyzed 3 h after the heme treatment. Data are shown as mean ± SEM of three independent experiments. Immunoblots are cropped from different parts of the same gel. Uncropped immunoblots are presented in the Supplementary information. Statistical analysis was performed by one-way ANOVA test followed by Bonferroni correction. A value of *p* < 0.05 was considered significant. *p < 0.05, ***p < 0.001.

Biliverdin reductase converts biliverdin (BV) to BR, the latter having remarkable antioxidant activity^28^. Depletion of biliverdin reductase A (BVRA) aggravates ROS formation and cell death^29^. Therefore, we questioned whether BVRA expression in ECs is also essential to establish tolerance to HIER stress. First, we showed that, unlike HO-1, BVRA depletion (Fig. 9A,D) did not aggravate HIER stress (Fig. 9B–D). We also found that BVRA knock-down failed to influence ER stress tolerance to HIER stress in response to HA (Fig. 9E–H). This reinforces the notion that heme degradation by HO-1 is the limiting factor in the establishment of tolerance against heme triggering ER stress.

### Exogenous carbon monoxide but not bilirubin ameliorates HIER stress

**Figure 10:**
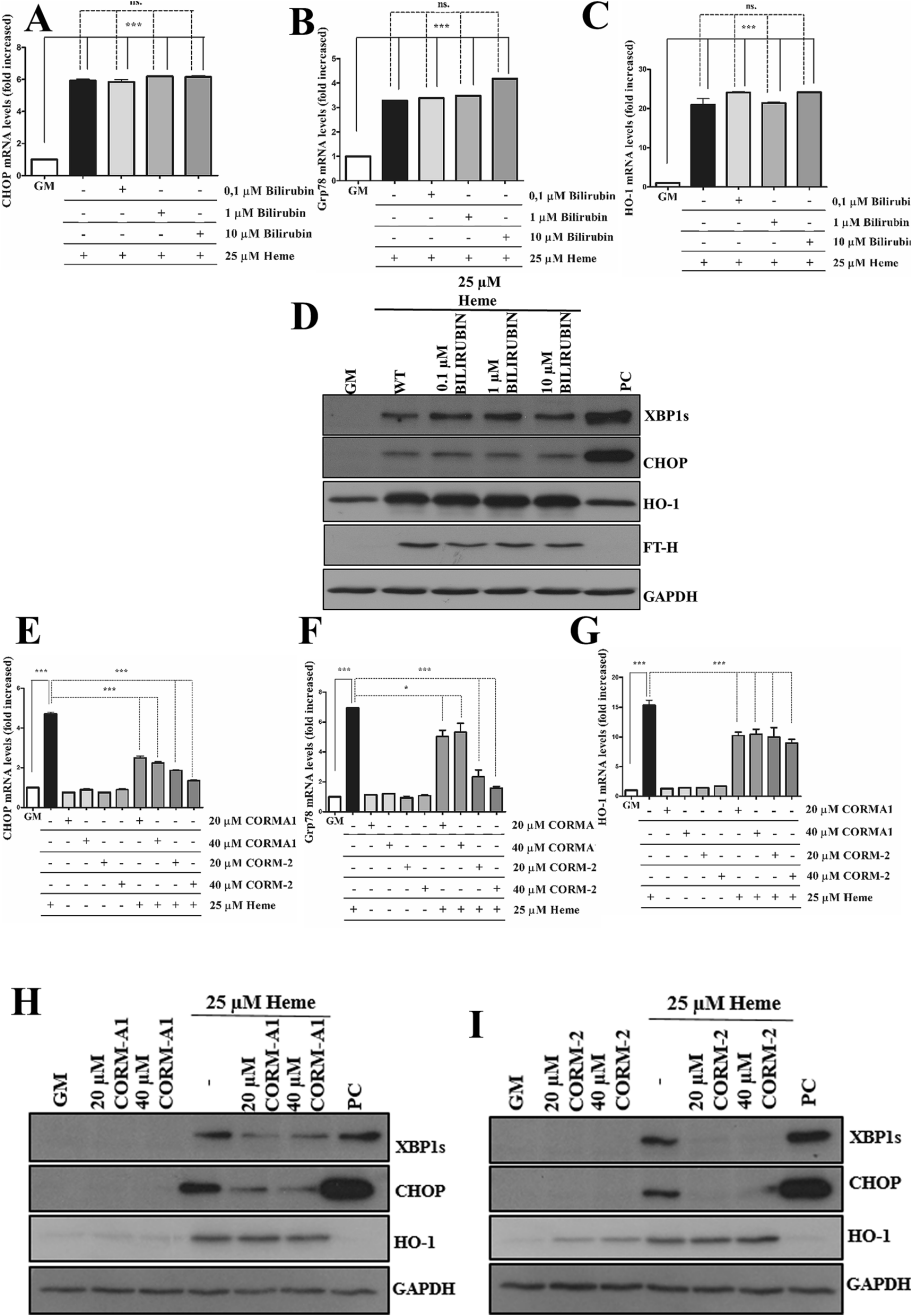
Exogenous carbon monoxide but not bilirubin attenuates HIER stress. ECs were treated with various concentration of bilirubin (0.1, 1, and 10 µM) in reduced serum medium containing 1% FBS (referring to 2.91 µM albumin) for 16 h, then cells were treated with heme (25 µM) for 2 h in serum- and antibiotics-free CM199. Then, the medium was changed to CM199 with 10% FCS and antibiotics. Relative expression of (**A**) CHOP, (**B**) Grp78, and (**C**) HO-1 mRNAs, as well as (**D**) spliced XBP1, CHOP, HO-1 and BVRA protein levels, were analyzed 3 h after the heme treatment. For CO experiments, ECs were pre-treated to various doses (20 and 40 μM) of CORM-2 or CORMA1 in CM199 supplemented with 2% FCS and antibiotics for 6 h, then ECs were treated with heme (25 μM) for 2 h in serum- and antibiotics-free CM199. Then, the medium was changed to CM199 with 10% FCS and antibiotics. Relative expression of (**E**) CHOP, (**F**) Grp78, and (**G**) HO-1 mRNAs as well (**H**,**I**) spliced XBP1, CHOP, HO-1, and BVRA protein levels were analyzed 3 h after the heme treatment. Immunoblots are cropped from different parts of the same gel. Uncropped immunoblots are presented in the Supplementary information. Data are shown as mean ± SEM of three independent experiments. Statistical analysis was performed by one-way ANOVA test followed by Bonferroni correction. A value of *p* < 0.05 was considered significant. ***p < 0.001.

BR protects ECs against a wide range of stress stimuli (Table 2). As knocking down BVR did not influence HIER stress, we examined whether exogenous BR inhibits HIER stress. In these experiments, we used the same concentration range of BR that has been applied in studies with ECs (Table 2). As demonstrated in Fig. 10A–D, exogenous BR did not attenuate the expression of CHOP and XBP1s in response to heme.

**Table 2:** Protective role of bilirubin against variety of cellular stresses in endothelial cells.

Cell type	BR concentration	Effect	References
Human coronary artery endothelial cells	1, 5, and 10 µM BR	Preincubation of HCAECs with BR protects against TNF-α-mediated endothelial inflammation	^70^
Human glomerular endothelial cell	10 µM	BR significantly attenuates vascular endothelin-1 release in placental ischemia model	^71^
Bovine aortic endothelial cells	0.5 µM	BR protects endothelial cells against high glucose-induced damage	^72^
Human aortic endothelial cells	5 µM	BR attenuates vascular endothelial activation and dysfunction	^73^
Cerebral microvascular endothelial cells	1 µM	BR prevents TNF-α-induced apoptosis	^74^

CO, a gasotransmitter, is released during heme degradation by HO-1 and has remarkable anti-inflammatory and anti-apoptotic potential^12^. Therefore, we next explored whether CO is involved in the protective effect of HO-1 induction before HIER stress. ECs were exposed to various concentrations (20 and 40 µM) of CO donors releasing CO with different kinetics (CORMA1—slow releaser, CORM-2 fast releaser). Comparing the two different CORMs, we showed that CORM-2 more effectively attenuated HIER stress compared to CORMA1 (Fig. 10E–I). Unlike CORMA1, CORM-2 induced HO-1 expression which is possibly involved in its more efficient protective effect (Fig. 10D,E). Notably, at the mRNA level, both CORMs only slightly induced HO-1 (Fig. 10G). These results suggest that exogenous CO but not BR ameliorates HIER stress in ECs.

### HO-1 but not FT-H and BVRA is the ultimate cytoprotectant in heme-induced cell death

**Figure 11:**
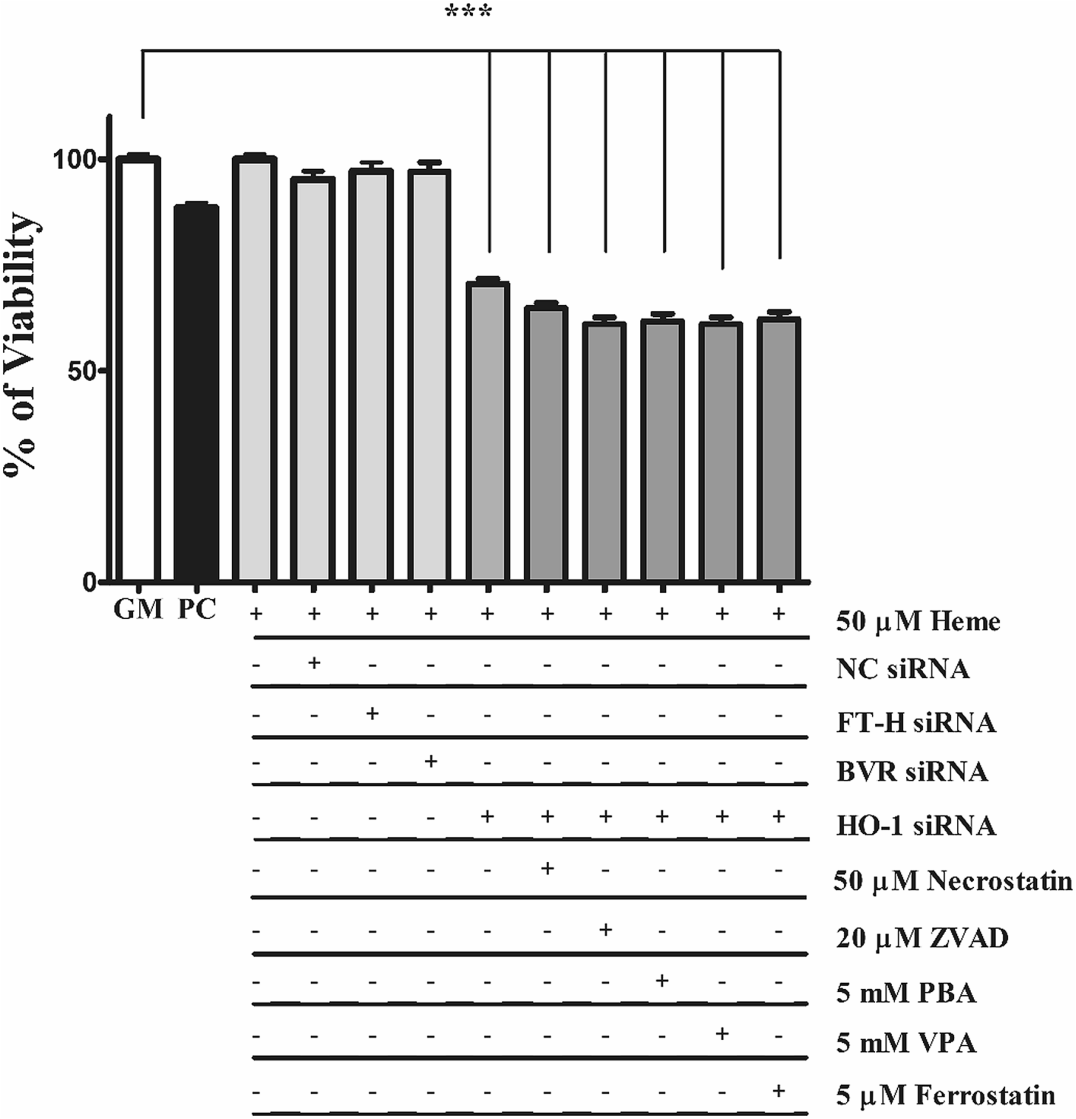
Knocking down HO-1 induces cell death in EC cultures that cannot be counterbalanced by currently known cell death inhibitors. Cells were plated on 96-well black plates and silenced as described above. ECs were exposed to heme (50 μM) in a culture medium containing 5% FBS. Parallel with that, ECs were exposed to well-known inhibitors of necroptosis (50 μM necrostatin), apoptosis (20 μM ZVAD), and ferroptosis (5 μM ferrostatin), or, ER stress inhibitors (5 mM phenylbutyric acid or 5 mM valproic acid) for 30 min in complete growth medium with 10% FCS, then exposed to heme (50 μM) for 24 h in CM199 with 5% FCS. Cells were then washed with HBSS+ and calcein AM was added to the cell in a final concentration of 1 µM and further incubated for 30 min in HBSS+. Fluorescence was measured at Ex/Em = 485/530 nm. Data are shown as mean ± SEM of three independent experiments. Statistical analysis was performed by one-way ANOVA test followed by Bonferroni correction. A value of *p* < 0.05 was considered significant. ***p < 0.001.

HO-1 deficiency or knocking down HO-1 dramatically decreases viability when cells are exposed to heme^30,31^ Given that the protective HO-1 system involves not only the heme-catabolizing HO-1 but also other important proteins such as BR-producing BVR and iron sequestering ferritin. Therefore, we next focused whether BVR or FT-H are vital components of the protective HO-1/ferritin system. To test whether knocking down FT-H or BVRA also affects viability or whether the lack of HO-1 is compensated by cell death inhibitors, FT-H, BVRA, and HO-1 expression was knocked down by specific si-RNAs (Fig. 7) followed by exposure to heme (50 μM) in culture medium containing 5% FBS. We showed that HO-1 silencing reduced cell viability by about 40%, but neither FT-H nor BVR-A knock down decreased viability. Parallel with that, ECs were exposed to well-known inhibitors of necroptosis (50 μM necrostatin), apoptosis (20 μM ZVAD), and ferroptosis (5 μM ferrrostatin), or, ER stress inhibitors (5 mM phenylbutyric acid or 5 mM valproic acid) for 30 min in complete growth medium with 10% FCS, then exposed to heme (50 μM) for 24 h in CM199 with 5% FCS (Fig. 11). Our results indicate that none of the inhibitors attenuate cell death in HO-1-silenced cells. This suggests that HO-1 deficiency severely affects cell viability that cannot be rescued either with cell death or ER stress inhibitors.

## Discussion

In this study, we demonstrate the presence of HIER stress in atherosclerotic plaque development in human carotid arteries and provide data on how HIER stress can influence vascular EC injury. It suggests that HIER stress plays a part in diverse human pathologies associated with hemorrhage and hemolysis.

Intraplaque neovascularization plays a pivotal role in atherosclerotic plaque progression^32^. Due to weak newly formed neovasculture resulting in blood leakage and plaque hemorrhage^33,34^ followed by Hb and heme release^17,35^. ER stress is implicated in a myriad of diseases^14,15^, and also involved in atherosclerosis^16^ (Table 1). The analysis of human carotid artery samples from patients who underwent carotid endarterectomy showed high levels of heme in atherosclerotic plaques and ER stress in aortic smooth muscle cells^17^. This makes atherosclerotic plaque progression an ideal model to understand how HIER stress might contribute to the pathogenesis of human disorders with hemolysis and hemorrhage. Using an integrated genomic approach, here we found trends toward more severe ER stress during the progression of atherosclerosis in human carotid arteries, and this trend is associated with hemorrhage. Immunohistochemical analysis of hemorrhaged atherosclerotic plaques also revealed significant ER stress in the ECs and macrophages in the hemorrhaged area. These interesting findings support the concept that free heme aggravates ER stress in all resident cells of the vessel wall in hemorrhaged atherosclerotic plaques. Furthermore, recent research has shown in the murine model of intravascular hemolysis that hemoglobin/heme promote acute kidney injury with excessive ER stress^36^. This supports our hypothesis that HIER stress might be implicated in the pathogenesis of various diseases with hemorrhage.

Numerous human disorders are associated with massive hemorrhages followed by heme release^3,37^. Hemolysis and hemorrhage trigger a wide variety of stress stimuli in ECs (reviewed by Frimat et al.^38^. The sensitivity of ECs to heme-induced cell damage is also suggested by Yachie and co-workers who found that hemolysis targets the endothelium in human HO-1 deficiency^30^. Having established that the endothelium shows increased ER stress in hemorrhaged plaques, we focused our research on ECs which are the first innocent by-standers of hemolysis encountering a significant amount of free heme in hemolytic/hemorrhagic pathologies. To support our findings in human patients, we first determined the susceptibility of ECs against HIER stress. We showed that heme induced ER stress in ECs suggesting its potential pathogenic role in hemolytic/hemorrhagic human pathologies. Besides, heme also induces ER stress in the presence of serum components. Importantly, a sustained CHOP activation is detected in ECs when heme is permanently present in the experimental medium suggesting persistent ER stress. This supports our finding in carotid artery specimens where hemorrhage also induced ER stress not only in fresh but also in organized hemorrhage where intact red blood cells are no longer detectable. In addition to this, heme also contributes to EC dysfunction in several ways. Heme induces EC activation which increases the expression of adhesion molecules and cytokine production^39,40^, impairs angiogenesis by provoking paraptosis^41^.

4-PBA is generally used to treat urea cycle disorders, however, it is also a low molecular weight chemical chaperone that prevents misfolded protein aggregation and mitigates ER stress^42^. VPA is also known to alleviate ER stress triggered by a broad spectrum of stressors^43,44^. Here, we have demonstrated that HIER stress cannot be resolved neither by 4-PBA nor by VPA, on the contrary, ECs showed increased sensitivity towards HIER stress characterized by greater CHOP activation because of diminished HO-1 expression in 4-PBA and VPA-treated cells. Our observation is supported by Palsamy et al*.* who have found that VPA suppresses Nuclear factor erythroid 2-related factor 2 (Nrf2)-Kelch-like ECH-associated protein 1 (Keap1)-dependent antioxidant protection through induction of ER^45^. As Nrf2 is the master transcription regulator of antioxidant genes, among them HO-1^46^, it is reasonable to assume that this mechanism is also responsible for decreasing HO-1 expression and increasing CHOP activation in ECs exposed to heme.

Having considered that ER stress inhibitors did not ameliorated or even aggravated HIER stress, next we focused on the HO-1/ferritin system that has remarkable protective properties in diverse pathologies^47,48^. Considering this, we activated the HO-1/ferritin system before heme exposure with heme arginate (HA) that is far less vasculotoxic compared to hematin but efficiently induces the HO-1/ferritin system^49^ making HA an ideal candidate for the safe induction of the HO-1/ferritin system. Here we showed that a single, non-toxic dose of HA efficiently induced HO-1/ferritin expression and provided resistance against HIER stress in ECs supporting the concept that HA might protect against HIER stress and heme-driven cellular-and tissue injuries. This has also been shown by randomized clinical trials indicating that a single high dose of HA improves reperfusion during ischemia–reperfusion injury in humans^50^ and that intravenous HA infusion 24 h before surgery induces HO-1 accumulation in the cardiac tissue^51^. Importantly, combined treatment with HA, heme and siRNA antiHO-1 supports our observation that HA-mediated protection against HIER stress is dependent on HO-1 and its heme catabolizing activity.

Having established the protective role of the HO-1/ferritin system in HIER stress, we then explored whether HO-1 or ferritin responsible for this protective effect. Ferritin, an evolutionarily ancient protein is induced by iron liberated during heme catabolism. Ferritin is composed of light and heavy chains, the latter of which is responsible for preventing cellular damage triggered by reactive oxygen species (ROS)^52^. Ferritin also protects astrocytes, neuronal cells, and leukemia cells against heme/hemoglobin-induced cell damage^24–26^. Previously, we have shown that ferritin but not HO-1 protects ECs from heme-catalyzed free radical injury triggered by activated polymorphonuclear leukocytes or other free radical sources, and ferritin is the ultimate cytoprotectant^26^. Others have found that the overexpression of ferritin heavy chain in HO-1-knocked down immortalized human bronchial epithelial and A549 cells completely prevents heme-mediated cell damage^31^. Heme-induced protein aggregation in the cytosol with subsequent aggresome formation is also mediated by iron release, and ferritin prevents excessive aggresome formation^53^. In contrast, we showed that HIER is independent of iron release and ferritin effect, but heavily dependent on HO-1 expression. With this in mind, we postulate that heme can induce cell damage in multiple ways, and cells govern cellular protective responses to heme stress by different bandmaster proteins.

HO-2 has an important role in the regulation of EC homeostasis. ECs isolated fromHO-2(−/−) mice show increased oxidative stress, inflammation, and angiogenesis^54^. HO-2 also preserves EC viability during hypoxia^55^. Having similar catalytic mechanism and efficiency as HO-1^27^ and being constitutively expressed, it is reasonable to assume that HO-2 could be a stopgap in HIER stress until HO-1 is induced. This prompted us to examine whether knocking down HO-2 could exacerbate HIER stress. Here we show that knocking down HO-2 has no effect on HIER stress under our experimental conditions suggesting the primary role of HO-1 in coping with HIER stress in ECs.

A central question of this study is how HO-1 protects against HIER stress in ECs. Given its remarkable antioxidant activity^28,56^, BR should significantly contribute to the cytoprotective effect of HO-1 in HIER stress. Given that numerous studies have shown that BR protects ECs against a variety of stress stimuli in a wide concentration range (Table 2), it is reasonable to speculate that BR also provides tolerance to HIER stress. Here we demonstrate that BR does not protect against HIER stress in ECs. We postulate that this might be attributed to the intracellular localization of BR that is present in the nucleus and the cytosolic facing membrane of the ER, while the ER lumen is relatively BR depleted^57^. As the folding and quality control of secreted, plasma membrane and organelle proteins is taken place in the ER lumen^58^, we speculate that low luminal BR concentration in the ER might account for the fact why BR is ineffective to reduce HIER stress.

Biliverdin reductase (BVR) is a part of the protective HO-1 system by converting BV to BR. In an animal model of diabetes, HO-1 overexpression improves vascular function in a BVRA-dependent manner^59^. Others have found that the silencing of BVR caused increased oxidative stress and damage in human umbilical vein endothelial cells (HUVECs) challenged by lipopolysaccharide^60^. Here we showed that BVR is not essential to cope with HIER stress in ECs, and it is not involved in the HO-1-conferred protection of cells against HIER stress in HA-treated cells. We suggest that BVR and its end-product BR have a limited role in resolving HIER stress.

Considering that neither BR nor BVR influenced HIER stress in ECs, we focused our work on the heme degradation product carbon monoxide (CO). Both CO and CO-releasing molecules (CORMs) have gained a lot of interest due to their anti-apoptotic, anti-ischemic, and anti-inflammatory effects^61,62^. CO is also implicated in the regulation various biological processes such as vascular tone^63^ and inflammation^64^. To study the in vitro effects of CO in HIERS, we chose two types of CORMs, CORMA1 and CORM-2, which are characterized by different CO-releasing kinetics; CORMA1 releases CO at a much slower rate under physiological conditions (half-life of approximately 21 min at 37 °C and pH 7.4)^65^, while CORM-2 releases CO at a fast rate (less than 1 min)^66^. Here we showed that both CORMs confer protection against HIER stress in ECs. This is in good agreement with a previous study that has shown that CORM-2 induces HO-1 expression in HUVECs and protects against ER stress-induced by homocysteine as well as thapsigargin and tunicamycin^67^. An interesting finding of our work is that CORM-2 but not CORMA1 induced HO-1 protein expression and CORM-2 provided more efficient protection. We speculate that the more pronounced protection by CORM-2 is associated with its HO-1 inducing effect; however, it is noteworthy that CO can mitigate HIER stress without HO-1 induction through a currently unknown mechanism which highlights the potential therapeutic benefit of CORMs to prevent HIER stress.

HO-1 deficiency is extremely rare in humans. Only two cases have been reported characterized by hemolysis, inflammation, nephritis, asplenia, and in one of the two cases, early death^30,68^. ECs derived from an HO-1 deficient patient are severely damaged by heme^30^. In line with these results, we showed significant cell death in HO-1- knocked down ECs exposed to heme. Interestingly, neither BVR nor ferritin heavy chain (FT-H) knockdown was lethal suggesting that neither BVR nor FT-H are vital to the HO-1-mediated protection of ECs against heme-induced cell death suggesting that the protective effect of the HO-1/ferritin system is primarily dependent on HO-1 but not FT-H or BVR. Next, we asked whether heme-induced cell death could be inhibited by any of the currently known commercial cell death inhibitors. HO-1-deficient cells, based on study on the first human case with HO-1 deficiency by Yachie et al.^30^, are extremely sensitive to heme-induced cell death without a significant change in the amounts of heme in the cell culture medium. This raised the question how cells die in heme toxicity in the absence of HO-1. An earlier report has shown that necrostatin-1 (an inhibitor of necroptosis) blocks heme-induced cell death in murine macrophages^69^. Therefore, we next asked whether heme-induced cell death could be inhibited by any of the currently known commercial cell death or ER stress inhibitors in HO-1-knocked down ECs. We have shown that ferroptosis, necroptosis as well apoptosis and ER stress inhibitors are ineffective to attenuate heme-induced cell in EC culture when HO-1 is knocked down. This is in good agreement with a previous study which has shown that neither ferrostatin nor nectrostatin-1 is effective in heme-induced cytotoxicity in HO-1-silenced cells^31^. These results indicate that heme-induced cell death in HO-1-knocked down ECs might be unique and remains to be still elusive. We suggest that heme-induced cell death occurs via HIER stress.

In conclusion, this study demonstrates the existence of HIER stress, a previously unsuspected heme-driven cell and tissue damage mechanism of heme stress in ECs, and its possible etiologic role in human atherosclerosis. Based on these observations, we postulate that HIER stress might be a pathogenic factor in other disorders involving hemorrhage and hemolysis. Our observations may raise the idea that free heme can induce special cell and organ damage based on its subcellular localization. This hypothesis is supported by the fact labile heme can be detected in the ER, the plasma membrane, mitochondria, nucleus, and the cytosol as elegantly demonstrated by Hamza’s group^70^. Further research is needed to reveal free heme-induced cell damages in a subcellular compartment-specific manner. Our results also highlight the importance of the ER-specific localization HO-1 to protect against extensive ER damage by heme. We propose that selective targeting of HIER stress by induction of HO-1 by HA or CORMs may be of therapeutic value in the treatment of heme-mediated disorders, and it is reasonable to speculate that the pathologic outcomes associated with hemorrhage might be, at least partly, due to ER stress.

## Methods

### Reagents

Reagents were purchased from Sigma-Aldrich (St. Louis, MO, US) unless otherwise specified. Hemin chloride stock solution (2 mM) was prepared protected from light in sterile 20 mM NaOH on the day of use for each experiment. Heme arginate derived from Orphan Europe.

### Cell culture

Human aortic endothelial cells (HAoECs) were obtained from Lonza (Allendale, NJ, US). Cells were grown in EBM Endothelial growth medium supplemented with 10% FBS, 100 U/mL penicillin, 100 µg/mL streptomycin, and amphotericin B. They were grown to 90% confluence and used from passages 4 to 6. The medium was changed every 2 days. Heme treatments were carried out in serum- and antibiotic-free CM199 medium. Briefly, hemin chloride stock solution in sterile 20 mM NaOH was diluted in serum- and antibiotic-free CM199. Cells were washed twice with Hank’s Balanced Salt Solution pH 7.4 supplemented with Ca^2+^ and Mg^2+^ (HBSS+) and treated with different hemin concentrations for 2 h. Cells were then washed with HBSS+ and fresh CM199 with 10% fetal bovine serum (FBS) and antibiotics were added and cells were further incubated for 3 h, 6 h, and 16 h in a CO_2_ (5%) incubator. Cells treated with 1 µM of thapsigargin, a non-competitive inhibitor of the sarco/endoplasmic reticulum Ca^2+^ ATPase (SERCA), for 3 h, 6 h, and 16 h as positive controls of ER stress.

To examine whether heme also induces ER stress in serum-containing medium, cells were exposed to heme (50 µM) in CM199 medium supplemented with 1 or 5% FBS, 100 U/mL penicillin, 100 µg/mL streptomycin, and amphotericin B for 3, 6, and 24 h. ER stress markers were then analyzed by qPCR or immunoblot as described above.

In some experiments, ECs were pre-incubated with either 4-PBA (5 mM) or VPA (5 mM) overnight, then exposed to heme (25 µM) in serum- and antibiotics-free CM199 supplemented with either 4-PBA (5 mM) or VPA (5 mM), then further incubated with CM199 supplemented with 10% FCS, antibiotics, and either 4-PBA (5 mM) or VPA (5 mM) for 3 h and 6 h.

For heme arginate (HA) experiments, cells were treated with 10 µM of HA in CM199 with 10% FCS and antibiotics overnight, then challenged with heme (25 µM) as described above. In some experiments, we pre-treated ECs with various concentrations of BR (0.1; 1, and 10 µM) in reduced serum medium containing 1% FBS (referring to 2.91 µM albumin) for 16 h, then cells were treated with heme (25 µM).

In another set of experiments, HO-1 expression was knocked down by siRNA antiHO-1 as described above, then ECs were pre-incubated with HA (10 µM) in CM199 medium supplemented with 10% FCS and antibiotics for 16 h. Then, ECs were washed and exposed to heme (10 µM) for 2 h in CM199 medium without FCS and antibiotics. ECs were then washed with HBSS+ and further incubated with CM199 supplemented with 10% FCS and antibiotics.

For CO experiments, CORM-2 was dissolved in DMSO, while CORMA1 in cell culture grade water right before the experiments. ECs were pre-treated to various doses (20 and 40 μM) of CORM-2 or CORMA1 in CM199 supplemented with 2% FCS and antibiotics for 6 h, then ECs were treated with heme (25 μM) as described above.

### Cell lysis and immunoblot

Cell lysis and immunoblots were performed as previously described^17^. Briefly, cells were washed with cold phosphate-buffered saline pH 7.4 then lysed with RIPA buffer containing protease and phosphatase inhibitors (50 mM Tris pH 7.5, 150 mM NaCl, 1% Igepal CA-630, 1% Sodium-deoxycholate, 0.1% SDS, 1× Complete Mini Protease Inhibitor Cocktail, 1× PHOSSTOP phosphatase inhibitor cocktail, and incubated for 15 min on ice. Lysates were clarified by spinning at 16,000×g, 4 °C for 15 min. Protein content was determined using the bicinchoninic acid assay (Pierce BCA Protein Assay Kit, Thermo Fisher Scientific, Waltham, Massachusetts, US). Cell extracts (30 µg protein) were electrophoresed on 10% Tris–glycine SDS-PAGE gels, then the proteins were transferred to 0.22 µm nitrocellulose membrane (GE Healthcare, Chicago, IL, US) and blocked with 5% w/v BSA or milk for 60 min according to the manufacturer’s guide. Primary antibodies against XBP1s and IRE1α (ERN1) from Cell Signaling Technology (Danvers, MA, US) were diluted 1:1000, Grp78 from Proteintech Group (Manchester M3 3WF, United Kingdom) and ATF-5 (Abcam, Cambridge, United Kingdom) were diluted 1:5000, while BLVRA antibody (Novus Biologicals, Centennial, CO, US) was diluted 1:1000 in blocking solution at 4 °C overnight. Membranes were then stripped and reprobed with HO-1 antibody (Proteintech Group, Manchester M3 3WF, United Kingdom) at a dilution of 1:5000 or HO-1 antibody (Proteintech Group, Manchester M3 3WF, United Kingdom) and ferritin heavy chain (FTH) antibody (Cell Signaling Technology, Danvers, MA, US) at a dilution of 1:1000. To ascertain equivalent protein loading in the samples, the membranes were stripped and reprobed again with a mouse anti-human GAPDH antibody (Proteintech Group, Manchester M3 3WF, United Kingdom) at a dilution of 1:10,000, The antigen–antibody complex was detected by WesternBright ECL HRP substrate (Advansta, Menlo Park, California, US).

### RNA isolation and quantitative reverse transcription‐polymerase chain reaction

RNA isolation and quantitative reverse transcription‐polymerase chain reaction were performed as previously described^17^. Briefly, cells were grown on six‐well plates and total RNA was isolated with TriReagent (Zymo Research, Irvine, CA, US) and reverse-transcribed using High-Capacity cDNA Reverse Transcription Kit (Applied Biosystems Inc., Foster City, CA, US). ERN-1, HO-1, ATF5, CHOP, Grp78, BVRA, HO-1 and GAPDH mRNA expressions were determined by TaqMan Gene Expression Assays (Thermo Fisher Scientific, Waltham, Massachusetts, US) and were normalized to GAPDH (ERN1: Hs00980097_m1;HO-2: Hs01558390_m1; ATF5:Hs01119208_m1; CHOP: Hs00358796_g1; Grp78: Hs00607129_gH; HO-1: Hs01110250; BVRA: Hs00167599_m1; GAPDH: Hs02758991_g1). In some experiments, GAPDH was normalized to PGK1 (Hs00943178_g1), TBP (Hs00427620_m1), and ACTB (Hs99999903_m1). Reverse transcriptions and qPCRs were carried out using the C1000 Thermal Cycler with CFX 96 Real-Time PCR System (Bio‐Rad, Hercules, CA, US). Relative mRNA expressions were calculated with the ΔΔCt method using GAPDH as an internal control.

### Tissue samples

Our model deals with groups of patients, rather than a patient’s disease history, to analyze HIER stress in healthy subjects and patients who underwent carotid endarterectomy due to atherosclerosis. Carotid arteries from patients who underwent carotid endarterectomy were obtained from the Department of Surgery at the University of Debrecen. The sample collection was approved by the Scientific and Research Ethics Committee of the Scientific Council of Health of the Hungarian Government under the registration number of DE OEC RKEB/IKEB 3712-2012. Written informed consent was received from the participants. Specimens were examined by a trained pathologist and classified according to AHA guidelines. Type I (healthy), IV (atheromatous), and VI (complicated) lesions were selected for further investigation.

### Immunohistochemistry

Immunohistochemistry was performed as previously described^17^. Briefly, the common carotid artery specimens were fixated with PBS buffered formaldehyde (4%) solution (4%) at pH 7.4 for 1 to 3 days—based on the size of the sample. In the case of calcified samples, 1.0 M/l EDTA/Tris buffer was used for the decalcification after fixation. The vascular segments were embedded in paraffin wax, then 3–5 μm thick slides were prepared through deparaffination used by xylene and ethanol. After inhibition of endogenous peroxidase (0.5% for 20 min) activity slides were subjected to antigen retrieval in a buffer solution (pH 9.0, RE7119, Leica, Wetzlar, Germany). For immunohistochemistry, samples were incubated with Dako EnVision FLEX Peroxidase-Blocking Reagent (Dako, Glostrup, Denmark) for 5 min in a wet chamber. Slides were then washed with EnVision FLEX Wash Buffer, Tris-buffered saline solution containing Tween 20, pH 7.6 (± 0.1). Antigen retrieval was performed in the epitope retrieval solution (RE-7119, Tris/EDTA-based buffer containing surfactant, Leica, Wetzlar, Germany) at pH 9 using a pressure cooker. Slides were then washed with distilled water and EnVision FLEX Wash Buffer, Tris-buffered saline solution containing Tween 20, pH 7.6 (± 0.1). Next, slides were incubated with CHOP (clone: rabbit polyclonal 15204-1-AP (Proteintech Group, Manchester M3 3WF, United Kingdom) primary polyclonal antibody at a dilution of 1:1000 used OPTIVIEW DAB DETECTION KIT based on protocol No. 6396500001. Next, slides were incubated with GRP78/BIP antibody (clone: rabbit polyclonal 11587-1-AP (Proteintech Group, Manchester M3 3WF, United Kingdom) primary polyclonal antibody at a dilution of 1:2000 used ULTRAVIEW UNIVERSAL DAB DETECTION KIT based on protocol No. 5269806001. The intensity and distribution of CHOP and GRP78/BIP specific immunostaining was assessed by light microscopy (Leica DM2500 microscope, DFC 420 camera, and Leica Application Suite V3 software, Leica). Counterstaining was performed with Gill Hematoxylin solution (105175 Merck Millipore, Billerica, MA, United States).

### RNA-sequencing

To obtain global transcriptome data of human carotid arteries biopsies from patients (*n* = 5) with atherosclerotic lesions, high throughput mRNA sequencing analysis was performed on Illumina Sequencing Platform (Illumina, San Diego, CA, USA). Total RNA was extracted and quantified from healthy carotid arteries, atheromas, and complicated lesions with hemorrhage, and RNA sample quality was checked on Agilent BioAnalyzer using Eukaryotic Total RNA Nano Kit (Agilent Technologies, Santa Clara, CA, USA) according to manufacturer’s protocol^71^. Sample preparation was performed as described earlier^71^. Briefly, samples with RNA integrity number (RIN) value greater than 8 were accepted for the library preparation process. RNA-Seq libraries were prepared from total RNA (200 ng) using NEBNext Ultra II RNA Sample Preparation Kit for Illumina (New England BioLabs, Ipswich, MA, USA) according to the manufacturer’s protocol. Briefly, poly-A tailed RNAs were purified by oligodT-conjugated magnetic beads and fragmented at 94 °C for 15 min. First-strand cDNA was generated by random priming reverse transcription and the second-strand synthesis step was performed to generate double-stranded cDNA. After the repairing ends and adapter ligation steps, adapter-ligated fragments were amplified in enrichment PCR, and finally, sequencing libraries were generated. The sequencing run was executed on Illumina NextSeq500 instrument (Illumina) using single-end 75 cycles sequencing. Aligned sequencing data have been deposited into the NCBI SRA database under accession no. PRJNA594843.

### Analysis of RNA-Seq data

Analysis of RNA-Seq data was performed as described earlier^71^. Briefly, raw sequencing data (fastq) was aligned to the human reference genome version GRCh37 using HISAT2 algorithm and BAM files were generated. Downstream analysis was performed using Strand NGS software, Version 3.4, Build 230243 (Strand Life Sciences, Bangalore, India; https://www.strand-ngs.com/). BAM files were imported into the software and DESeq1 algorithm was used for normalization. To identify differentially expressed genes atherosclerotic lesions and healthy biopsies, ANOVA with Tukey post hoc test was used. Heatmaps and dot plots were drawn using R packages pheatmap (R Core Team (2020). R: A language and environment for statistical computing. R Foundation for Statistical Computing, Vienna, Austria. URL http://www.R-project.org/.) and ggplot2^72^.

### Gene ontology analysis

Gene ontology analysis was performed as described earlier^71^. Briefly, lists of differentially expressed genes were analyzed using the Panther tool (http://www.geneontology.org/) and the GO Enrichment Analysis function to create a gene ontology (GO). GOs with fold enrichment > = 2 and p-value < 0.05 were selected and results were presented according to their -log10 p-value. Bar graph was drawn using R package ggplot2.

### siRNA transfection

Silencer select small interfering RNA specific to heme oxygenase-1, heme oxygenase-2, ferritin heavy chain, biliverdin reductase A and negative control siRNA were obtained from Ambion (Thermo Fisher Scientific, Waltham, Massachusetts, US). HAoEC transfection with siRNA was achieved using the Oligofectamine according to the manufacturer’s guide using 10 nmol/L siRNAs. (Thermo Fisher Scientific, Waltham, Massachusetts, US). Cells were then challenged with heme and the expression of ER stress markers was analyzed as described above.

### Confocal microscopy

Confocal microscopy was performed as previously described^17^. Briefly, cells were silenced or pretreated with HA then challenged with heme (10 µM) as described above. Cells were then washed with PBS pH 7.4 twice and fixed with 4% paraformaldehyde solution in PBS pH 6.9 for 15 min at 37 °C, then incubated with quenching solution pH 7.4 (100 mM Tris, 137 mM NaCl) for 10 min at room temperature. Coverslips were then washed twice with PBS pH 7.4, permeabilized with 0.1% Triton X-100 in PBS pH 7.4 for 15 min, washed again with PBS, and blocked for 60 min at room temperature with 10% goat serum and 2% BSA in PBS. Samples were then incubated sequentially with GRP78 Monoclonal Antibody (1H11-1H7) (Thermo Fisher Scientific, Waltham, MA, US) at a dilution of 5 µg/mL then with goat anti-mouse IgG conjugated to Alexa Fluor 488 (Thermo Fisher Scientific, Waltham, MA, US) at a dilution of 1:1000 for 60 min at room-temperature in antibody dilution buffer (1% BSA in PBS) with appropriate washing with PBS. Samples were then incubated with the HO-1 antibody (Manchester M3 3WF, United Kingdom) then with goat anti-rabbit IgG conjugated to Alexa Fluor 568 (Thermo Fisher Scientific, Waltham, MA, US) at a dilution of 1:1000 for 60 min. Nuclei were visualized with Hoechst. Samples were investigated with Lightning super-resolution microscopy using Leica Application Software X (Leica, Mannheim, Germany).

### Toxicity assay

Cells were plated on 96-well black plates and silenced as described above. ECs were exposed to heme (50 μM) in a culture medium containing 5% FBS. Parallel with that, ECs were exposed to well-known inhibitors of necroptosis (50 μM necrostatin), apoptosis (20 μM ZVAD), and ferroptosis (5 μM ferrostatin), or, ER stress inhibitors (5 mM phenylbutyric acid or 5 mM valproic acid) for 30 min in complete growth medium with 10% FCS, then exposed to heme (50 μM) for 24 h in CM199 with 5% FCS. Cells were then washed with HBSS+ and calcein AM was added to the cell in a final concentration of 1 µM and further incubated for 30 min in HBSS+. Fluorescence was measured at Ex/Em = 485/530 nm.

## Supplementary Information

Supplementary Figures.
