# Supplementary material for: Heme cytotoxicity is the consequence of endoplasmic reticulum stress in atherosclerotic plaque progression
Source: Sci Rep. 2021 May 17;11:10435. doi: 10.1038/s41598-021-89713-3 (PMC8129109; doi:10.1038/s41598-021-89713-3)

Supplementary figures

Heme cytotoxicity is the consequence of endoplasmic reticulum stress in atherosclerotic plaque progression

Dávid Pethő[1,2], Zoltán Hendrik[3,] Annamária Nagy[1,2], Lívia Beke[3], Andreas Patsalos[4,5], László Nagy[4,5], Szilárd Póliska[6], Gábor Méhes[3], Csaba Tóth[7], László Potor[8], John W. Eaton[9], Harry S. Jacob[10], György Balla[11], József Balla[1*], Tamás Gáll[1,8]

[1] Division of Nephrology, Department of Internal Medicine, Faculty of Medicine, University of Debrecen, Debrecen, Hungary

[2] Kálmán Laki Doctoral School, Faculty of Medicine, University of Debrecen, Debrecen, Hungary

[3] Department of Pathology, Faculty of Medicine, University of Debrecen, Debrecen, Hungary

[4] Department of Biochemistry and Molecular Biology, Faculty of Medicine, University of Debrecen, Debrecen, Hungary

[5] Departments of Medicine and Biological Chemistry, Johns Hopkins University School of Medicine and All Children's Hospital, St. Petersburg, FL USA

[6] Proteomics Core Facility, Department of Biochemistry and Molecular Biology, Faculty of Medicine, University of Debrecen, Debrecen, Hungary

[7] Division of Vascular Surgery, Department of Surgery, Faculty of Medicine, University of Debrecen, Debrecen, Hungary

[8] HAS-UD Vascular Biology and Myocardial Pathophysiology Research Group, Hungarian Academy of Sciences, University of Debrecen, Debrecen, Hungary

[9] James Graham Brown Cancer Center, University of Louisville, Louisville, KY, USA

[10] Department of Medicine, University of Minnesota, Minneapolis, MN, USA

[11] Department of Pediatrics, Faculty of Medicine, University of Debrecen, Debrecen, Hungary

Correspondence: *József Balla balla@belklinika.com

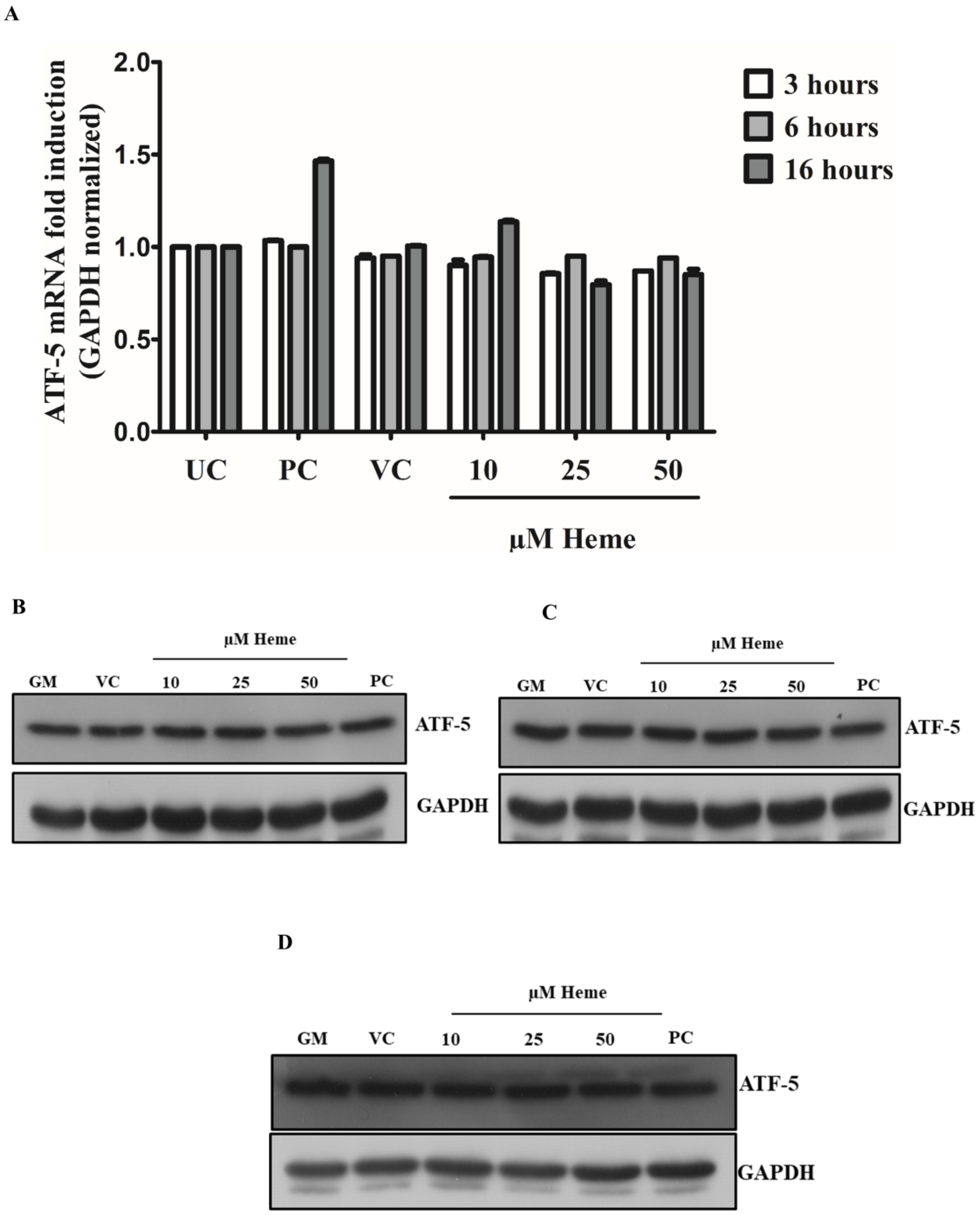

A
ATF-5 mRNA fold induction
(GAPDH normalized)
2.0
1.5
1.0
0.5
0.0
3 hours
6 hours
16 hours
UC
PC
VC
10
25
50
µM Heme
B
µM Heme
GM
VC
10
25
50
PC
ATF-5
GAPDH
C
µM Heme
GM
VC
10
25
50
PC
ATF-5
GAPDH
D
µM Heme
GM
VC
10
25
50
PC
ATF-5
GAPDH

Supplementary figure 1. Heme does not induces ATF-5 in EC cultures. ECs were treated with various doses of heme (10,25, and 50 µM) or corresponding vehicle solution to the highest heme dose (50 µM) in serum-and antibiotics-free CM199 for 2 h, then the medium was changed to CM199 with 10% FCS and antibiotics. Thapsigargin (1 µM) treated cells were used as ER stress control. Relative expression of ATF-5 mRNA ***(A,C,E)*** and protein ***(B,D,F)*** was analyzed after 3 ***(A,B)***, 6 ***(C,D)***, or 16 h ***(E,F)*** and normalized to GAPDH. Representative data of three independent experiments are shown representing ATF-5 levels 3, 6, and 16 h after the heme treatments. GM: growth medium; VC: vehicle control; PC: thapsigargin control. Data are shown as mean ± SEM of three independent experiments. Immunoblots are cropped from different parts of the same gel. Uncropped immunoblots are presented in the Supplementary information.

A

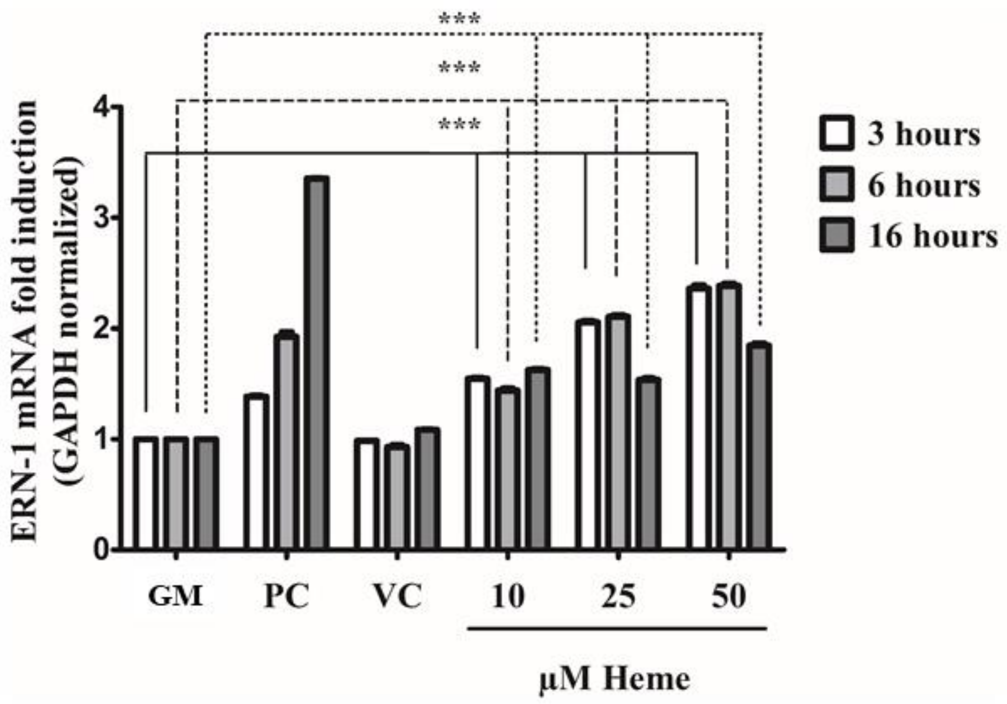

***
***
***
4
3
2
1
0
ERN-1 mRNA fold induction (GAPDH normalized)
3 hours
6 hours
16 hours
GM
PC
VC
10
25
50
µM Heme

B

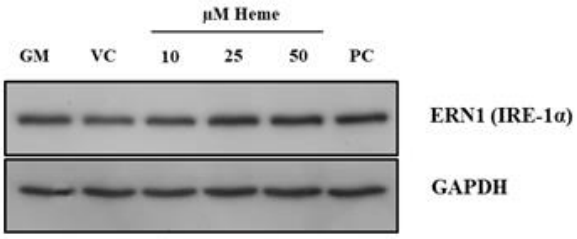

µM Heme
GM
VC
10
25
50
PC
ERN1 (IRE-1α)
GAPDH

C

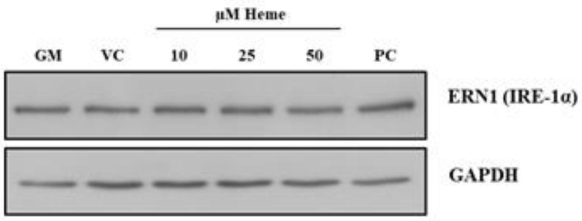

µM Heme
GM
VC
10
25
50
PC
ERN1 (IRE-1α)
GAPDH

D

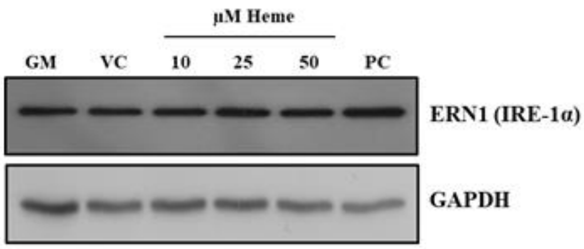

µM Heme
GM
VC
10
25
50
PC
ERN1 (IRE-1α)
GAPDH

Supplementary figure 2. Heme induces ERN1 mRNA but not protein expression in EC cultures. ECs were treated with various doses of heme (10,25, and 50 μM) or corresponding vehicle solution to the highest heme dose (50 μM) in serum-and antibiotics-free CM199 for 2 h, then the medium was changed to CM199 with 10% FCS and antibiotics. Thapsigargin (1 μM) treated cells were used as ER stress control. Relative expression of ERN1 mRNA ***(A,)*** and protein ***(B,C,D,)*** was analyzed after 3 ***(A,B)***, 6 ***(A,C)***, or 16 h ***(A,D)*** and normalized to GAPDH. Representative data of three independent experiments are shown representing ERN1 levels 3, 6, and 16 h after the heme treatments. GM: growth medium; VC: vehicle control; PC: thapsigargin control. Data are shown as mean ± SEM of three independent experiments. Immunoblots are cropped from different parts of the same gel. Uncropped immunoblots are presented in the Supplementary information. Statistical analysis was performed by one-way ANOVA test followed by Bonferroni correction. A value of $p < 0.05$ was considered significant. * $p < 0.05$, *** $p < 0.001$.

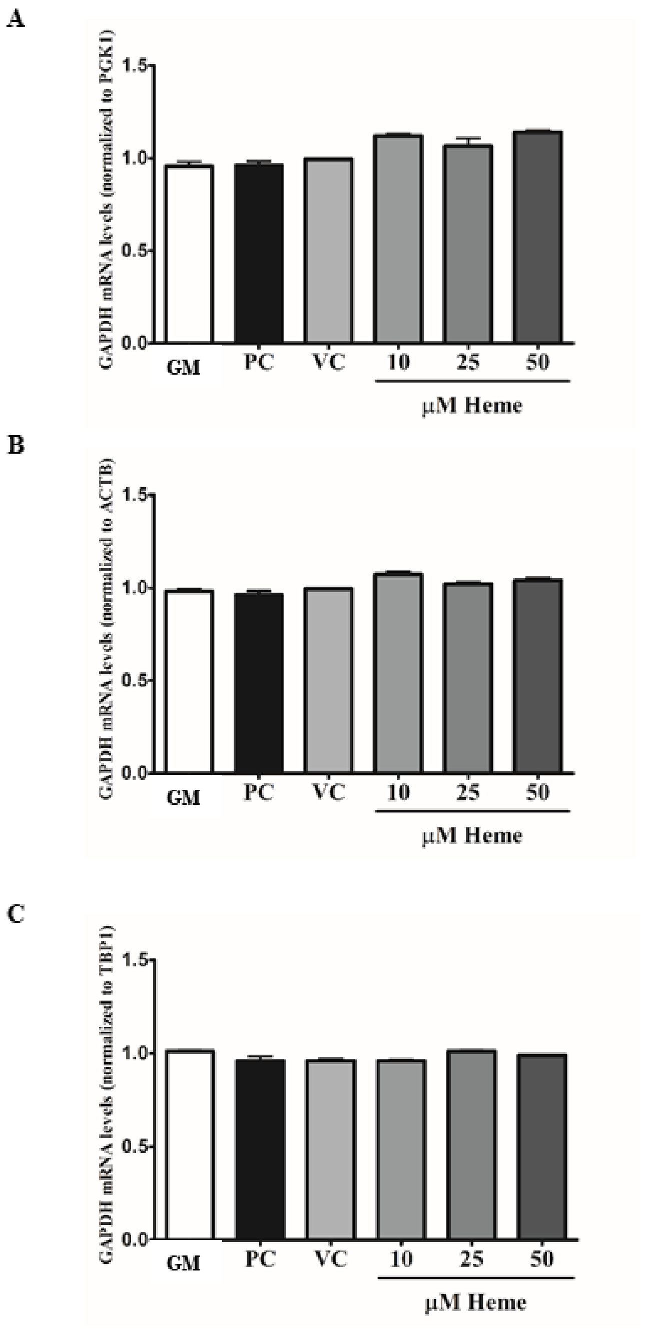
A
GAPDH mRNA levels (normalized to PGK1)
1.5
1.0
0.5
0.0
GM
PC
VC
10
25
50
μM Heme
B
GAPDH mRNA levels (normalized to ACTB)
1.5
1.0
0.5
0.0
GM
PC
VC
10
25
50
μM Heme
C
GAPDH mRNA levels (normalized to TBP1)
1.5
1.0
0.5
0.0
GM
PC
VC
10
25
50
μM Heme

Supplementary figure 3. Heme does not alter GAPDH expression under HIER stress conditions. Ecs to various doses of heme (10-50 μM) for 2 h in serum- and antibiotics-free CM199 medium followed by a 3-hour-incubation in CM199 medium containing 10% FCS and antibiotics followed by a qPCR analysis of GAPDH. GAPDH mRNA expression was then normalized to a set of other housekeeping genes such as Phosphoglycerate Kinase 1 (PGK1), β-actin, and TATA-binding protein 1 (TBP1).

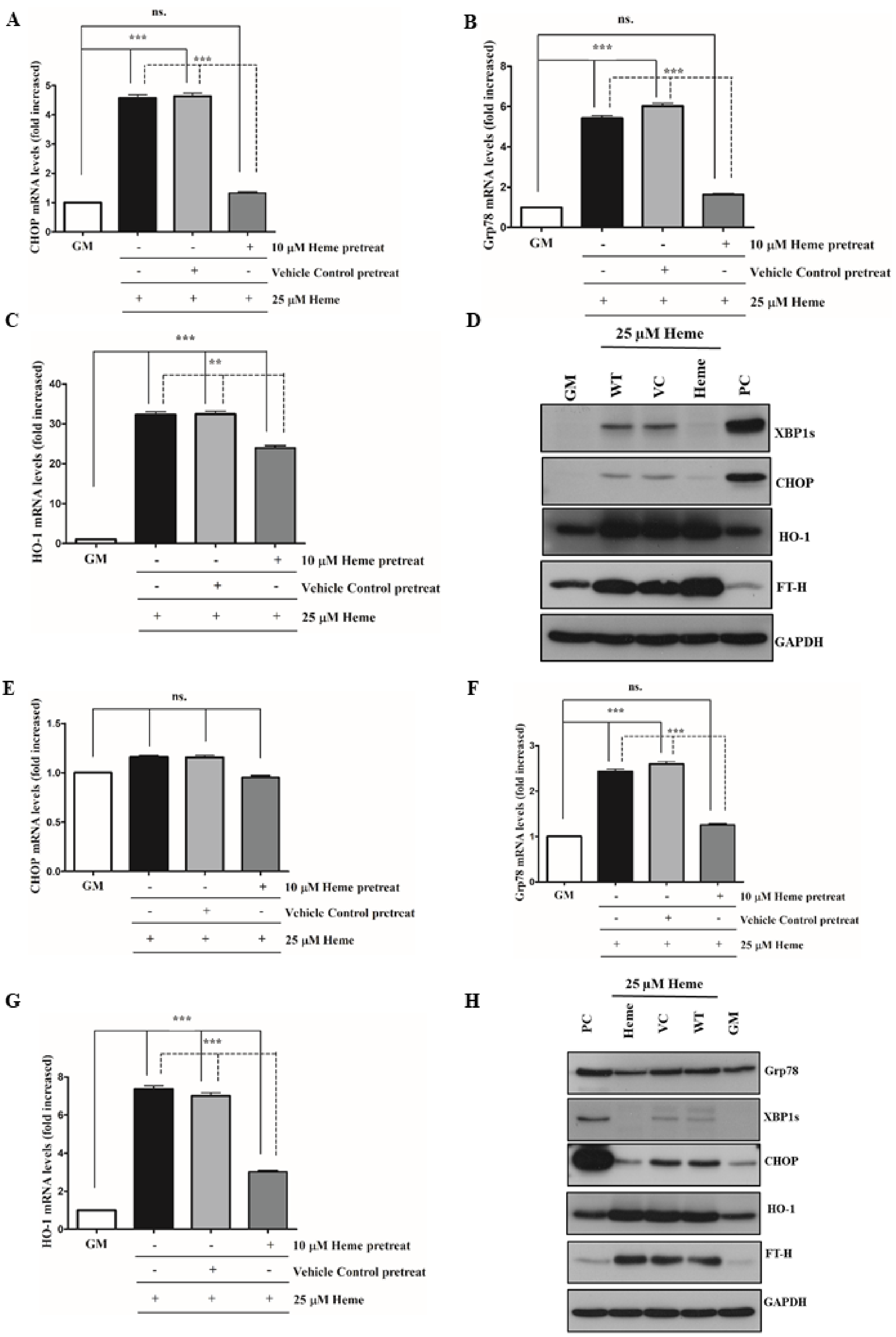

A
ns.
***
***
CHOP mRNA levels (fold increased)
GM
10 μM Heme pretreat
Vehicle Control pretreat
25 μM Heme
B
ns.
***
***
Grp78 mRNA levels (fold increased)
GM
10 μM Heme pretreat
Vehicle Control pretreat
25 μM Heme
C
***
**
HO-1 mRNA levels (fold increased)
GM
10 μM Heme pretreat
Vehicle Control pretreat
25 μM Heme
D
25 μM Heme
GM
WT
VC
Heme
PC
XBP1s
CHOP
HO-1
FT-H
GAPDH
E
ns.
CHOP mRNA levels (fold increased)
GM
10 μM Heme pretreat
Vehicle Control pretreat
25 μM Heme
F
ns.
***
***
Grp78 mRNA levels (fold increased)
GM
10 μM Heme pretreat
Vehicle Control pretreat
25 μM Heme
G
***
***
HO-1 mRNA levels (fold increased)
GM
10 μM Heme pretreat
Vehicle Control pretreat
25 μM Heme
H
25 μM Heme
PC
Heme
VC
WT
GM
Grp78
XBP1s
CHOP
HO-1
FT-H
GAPDH

Supplementary figure 4. Low-dose heme attenuates HIER stress in EC cultures. ECs were treated with 10 μM of heme in CM199 with 10% FCS and antibiotics overnight, then challenged with heme (25 μM) for 2 h in serum- and antibiotics-free CM199. Then, the medium was changed to CM199 with 10% FCS and antibiotics. Relative expression of *(A)* CHOP and *(B)* Grp78 mRNAs as well *(C)* HO-1 mRNA and *(D)* XBP1s, CHOP, HO-1, and ferritin heavy chain protein levels were analyzed 3 h after the heme treatment. In another set of experiments, relative expression of *(E)* CHOP, *(F)* Grp78, and (G) HO-1 mRNAs as well *(H)* Grp78, spliced XBP1, CHOP, HO-1, and ferritin heavy chain protein levels were analyzed 16 h after the heme treatment. GM: growth medium; HA: heme arginate; PC: positive control. Relative mRNA expressions were normalized to GAPDH. GAPDH was used as a loading control in immunoblots. Immunoblots are cropped from different parts of the same gel. Uncropped immunoblots are presented in the Supplementary information. Data are shown as mean ± SEM of three independent experiments. Statistical analysis was performed by one-way ANOVA test followed by Bonferroni correction. A value of $p < 0.05$ was considered significant. ***$p < 0.001$

Original blots

Figure 2C. Heme activates ER stress in EC cultures in a time- and dose-dependent manner - 3 h experiment.

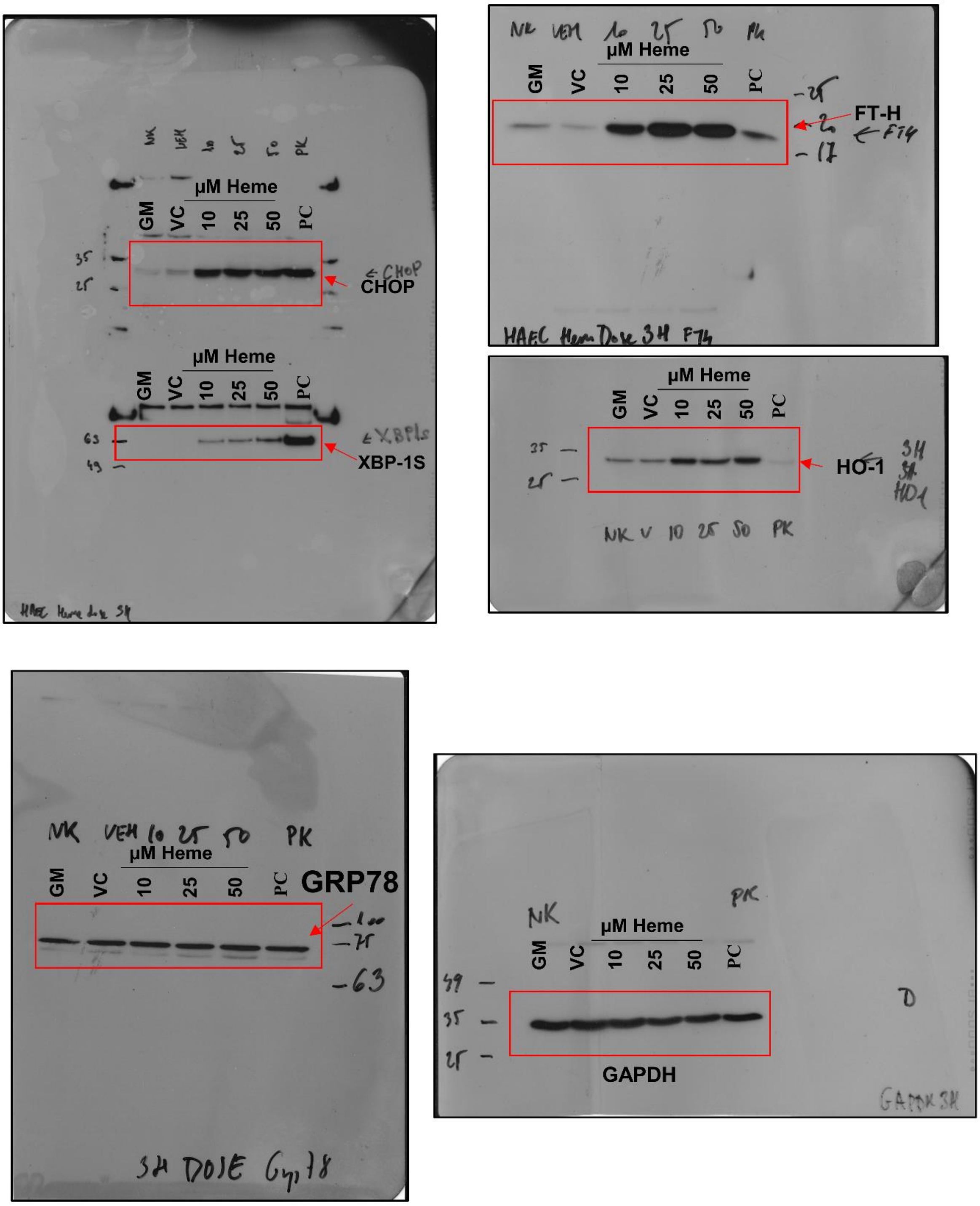

Figure 2D. Heme activates ER stress in EC cultures in a time- and dose-dependent manner - 6 h experiment.

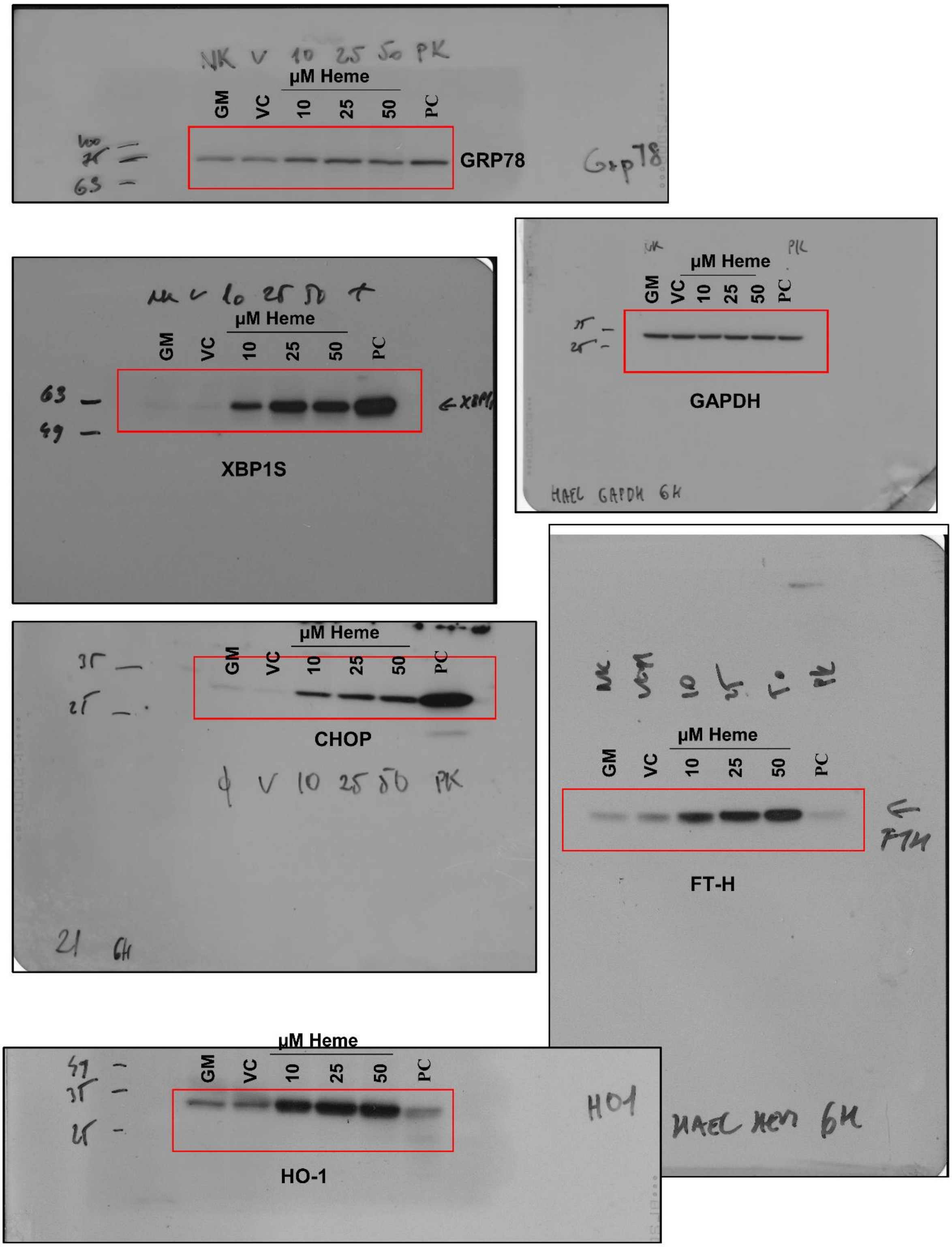

Figure 2E. Heme activates ER stress in EC cultures in a time- and dose-dependent manner - 16 h experiment.

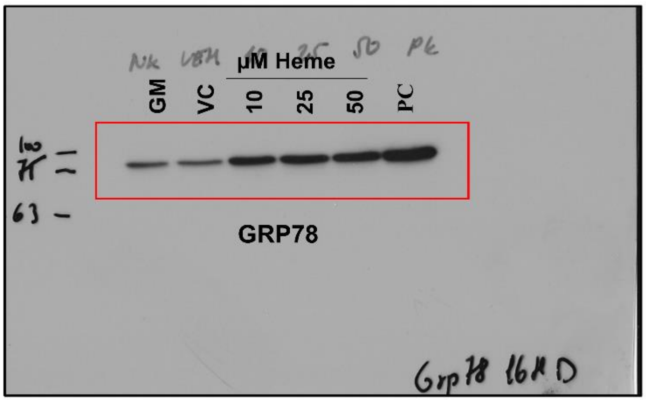

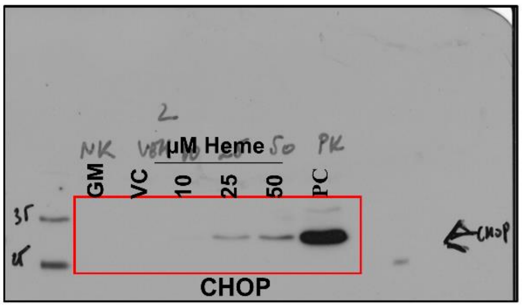

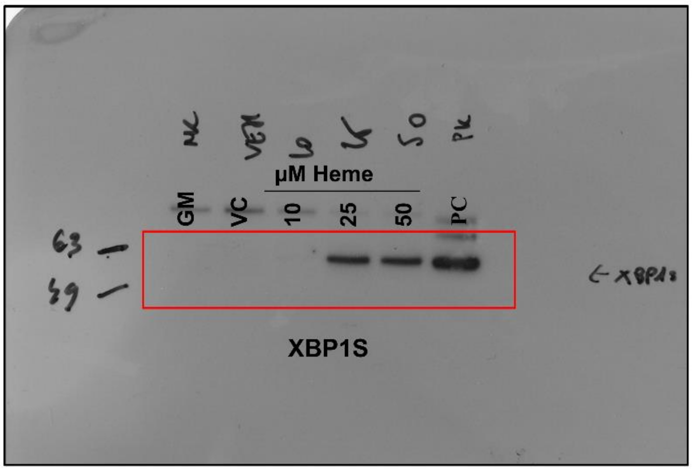

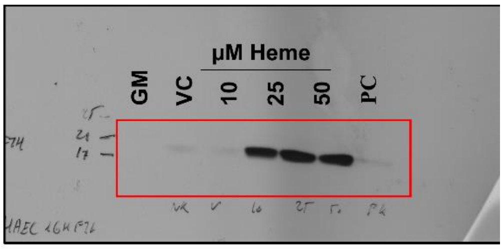

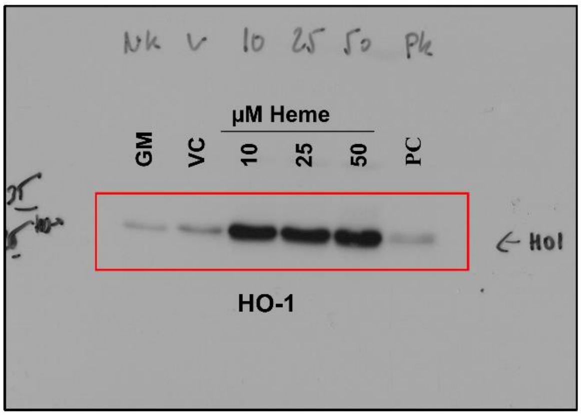

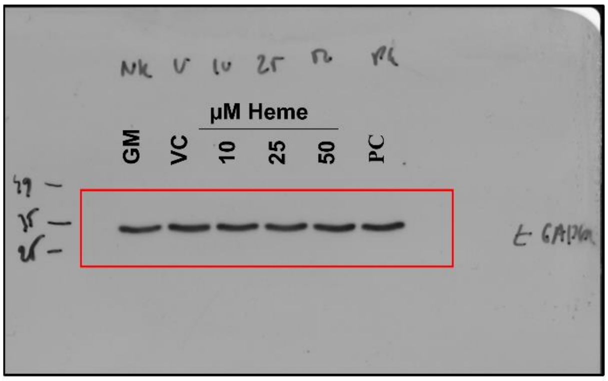

Figure 3G. Heme activates ER stress in EC cultures in the presence of serum.

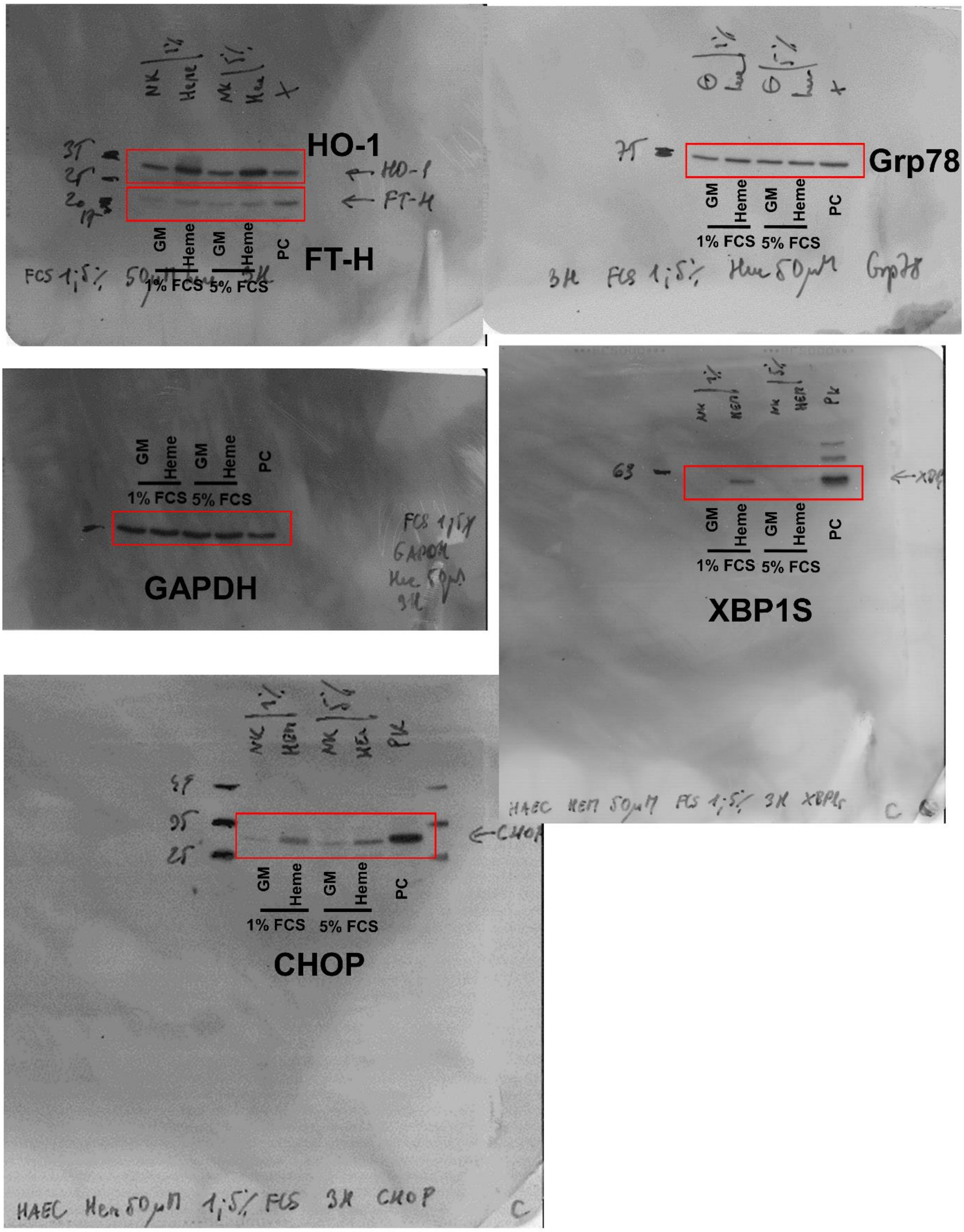

Figure 3H. Heme activates ER stress in EC cultures in the presence of serum.

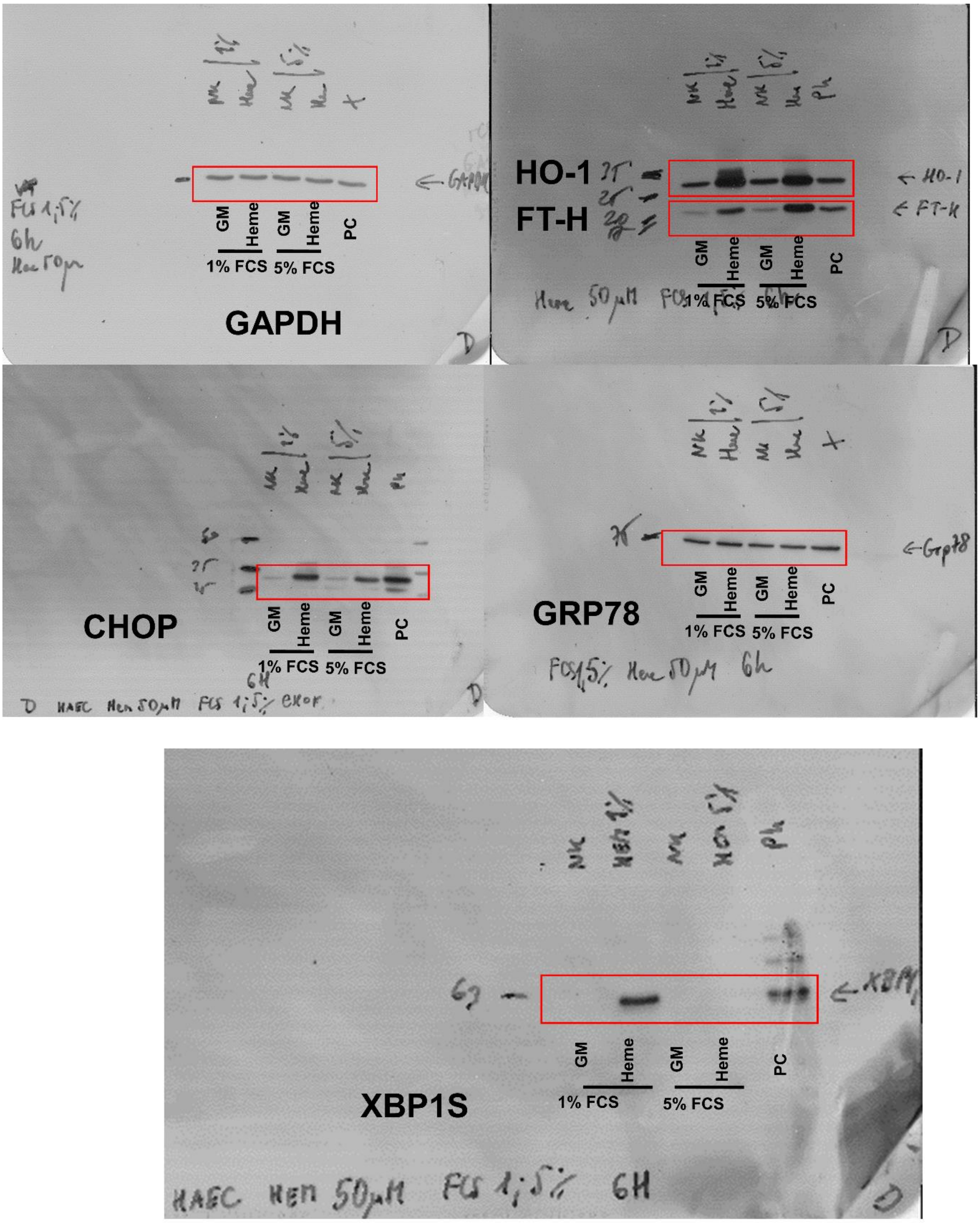

Figure 3I. Heme activates ER stress in EC cultures in the presence of serum.

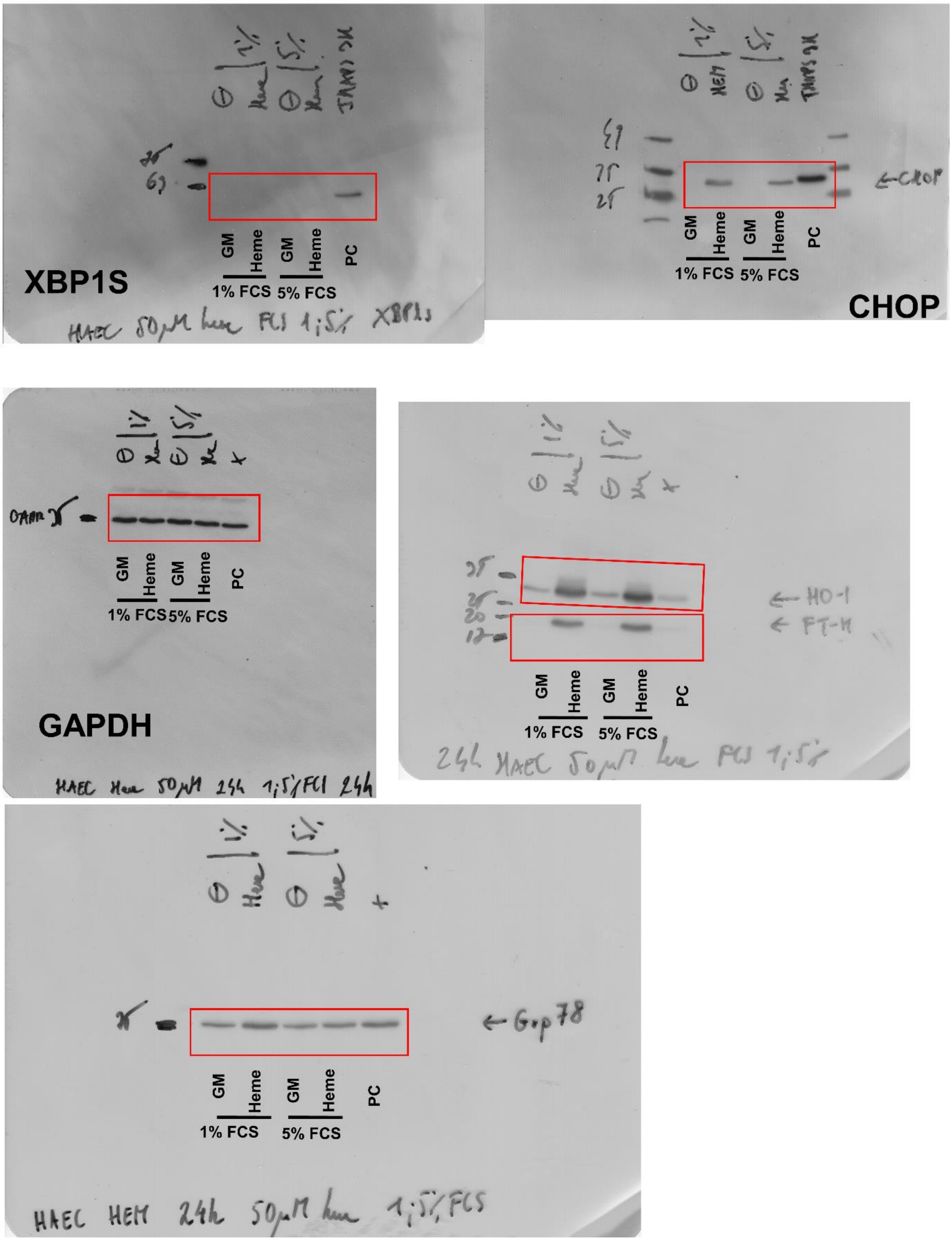

Figure 4D. ER stress inhibitors does not protect against HIER stress

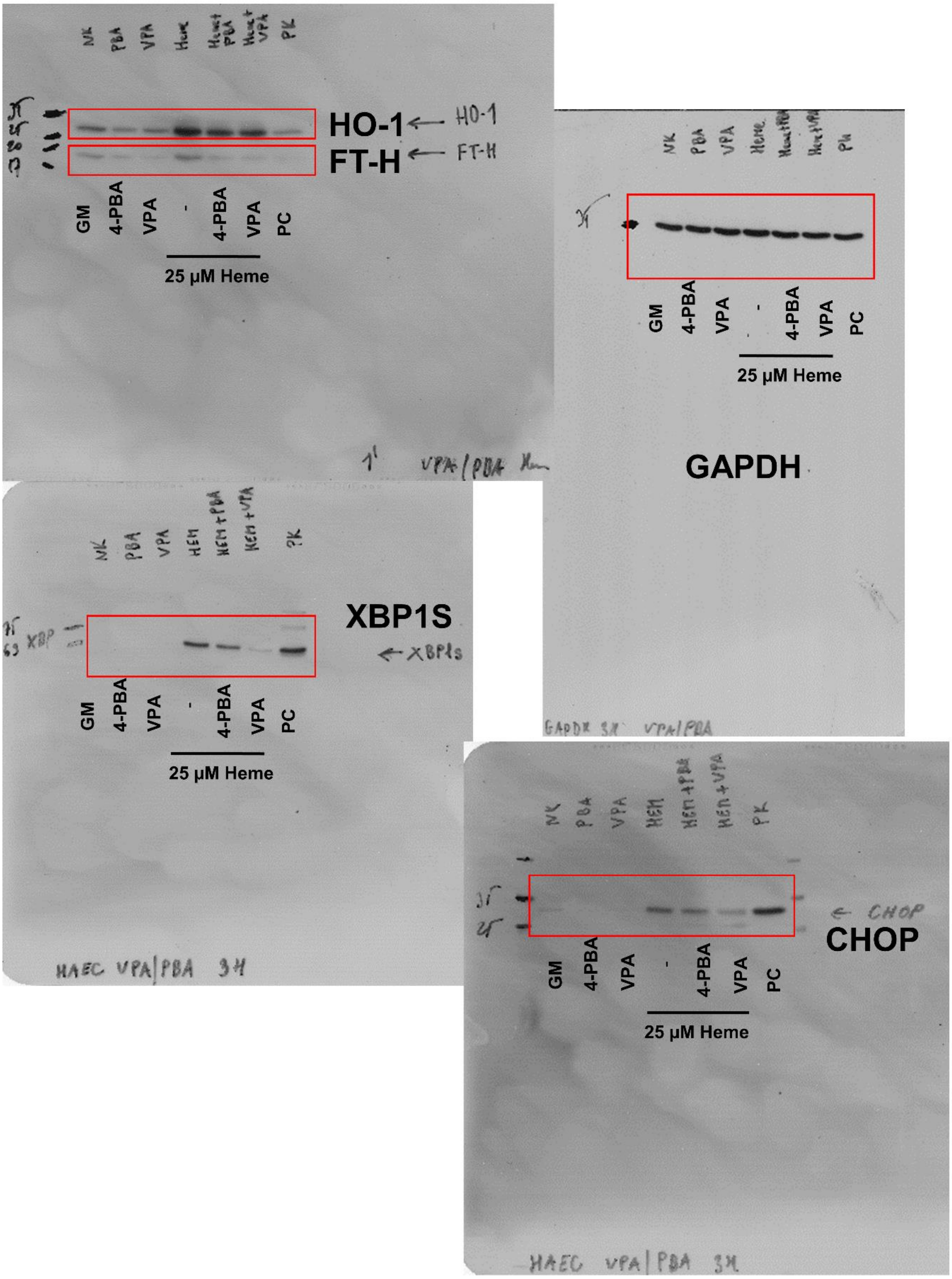

Figure 4H. ER stress inhibitors does not protect against HIER stress

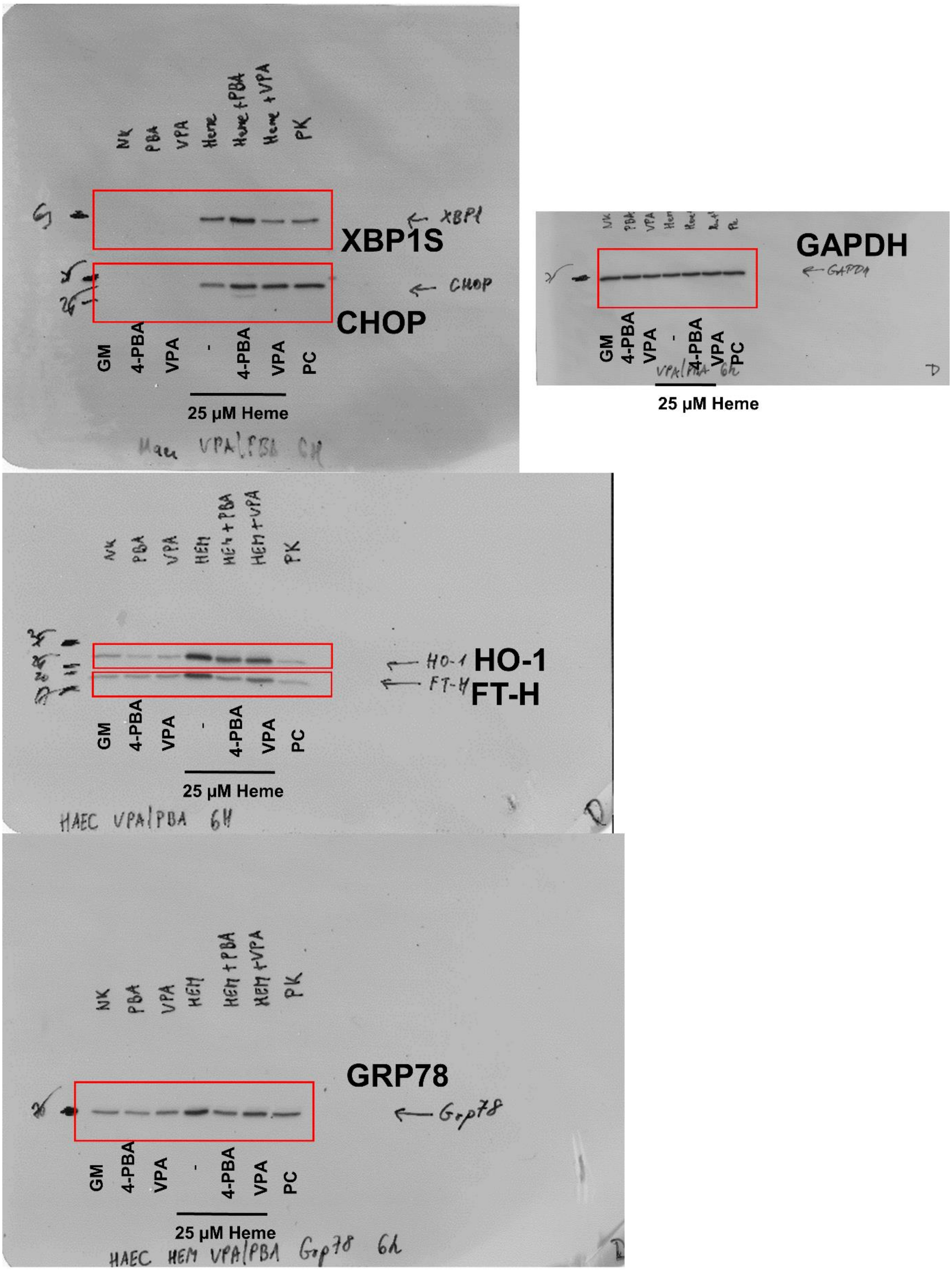

Figure 5C. Heme arginate attenuates HIER stress in EC cultures. Heme arginate pretreatment 3 h.

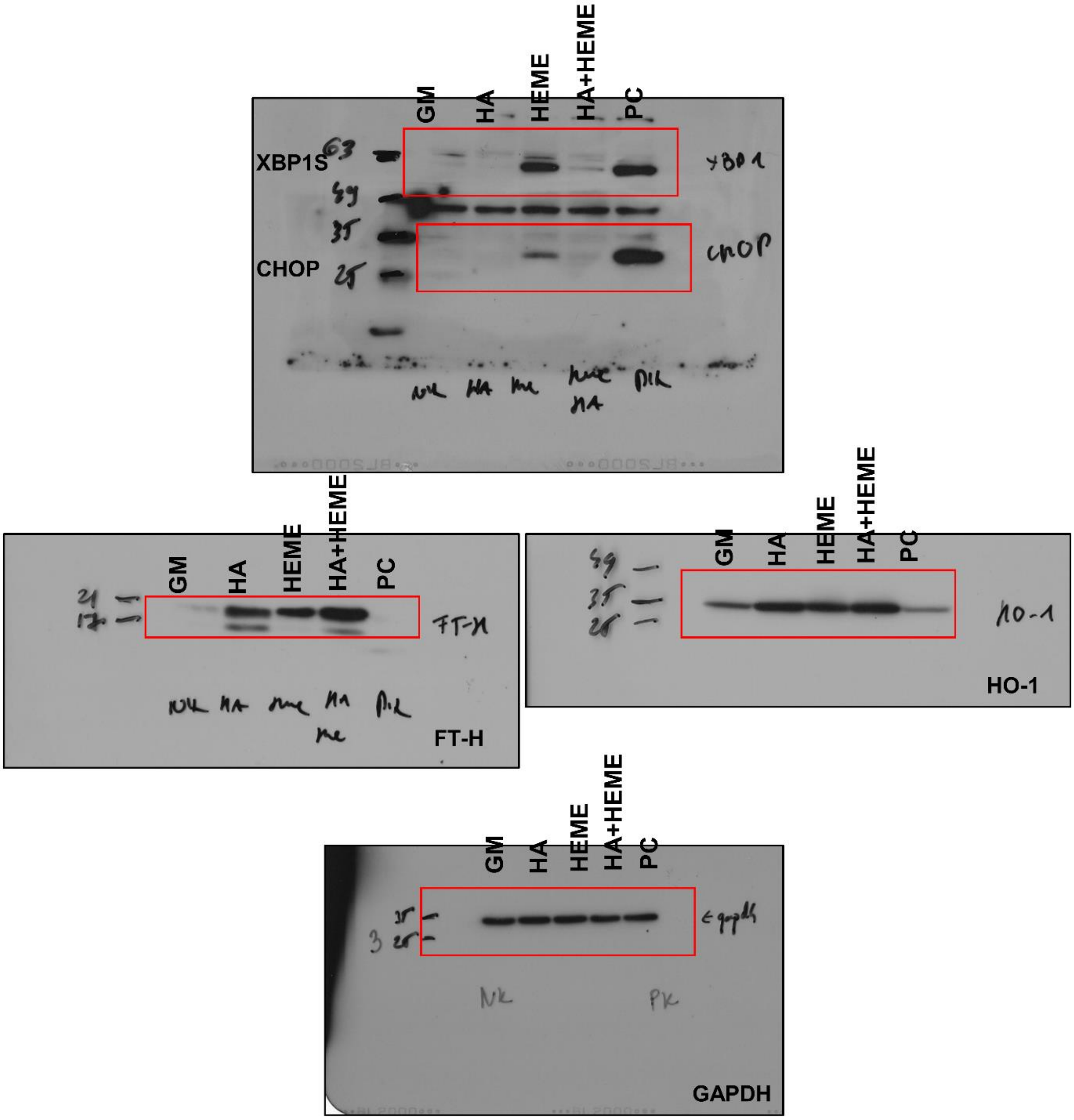

Figure 5F. Heme arginate attenuates HIIER stress in EC cultures. Heme arginate pretreatment 16 hr

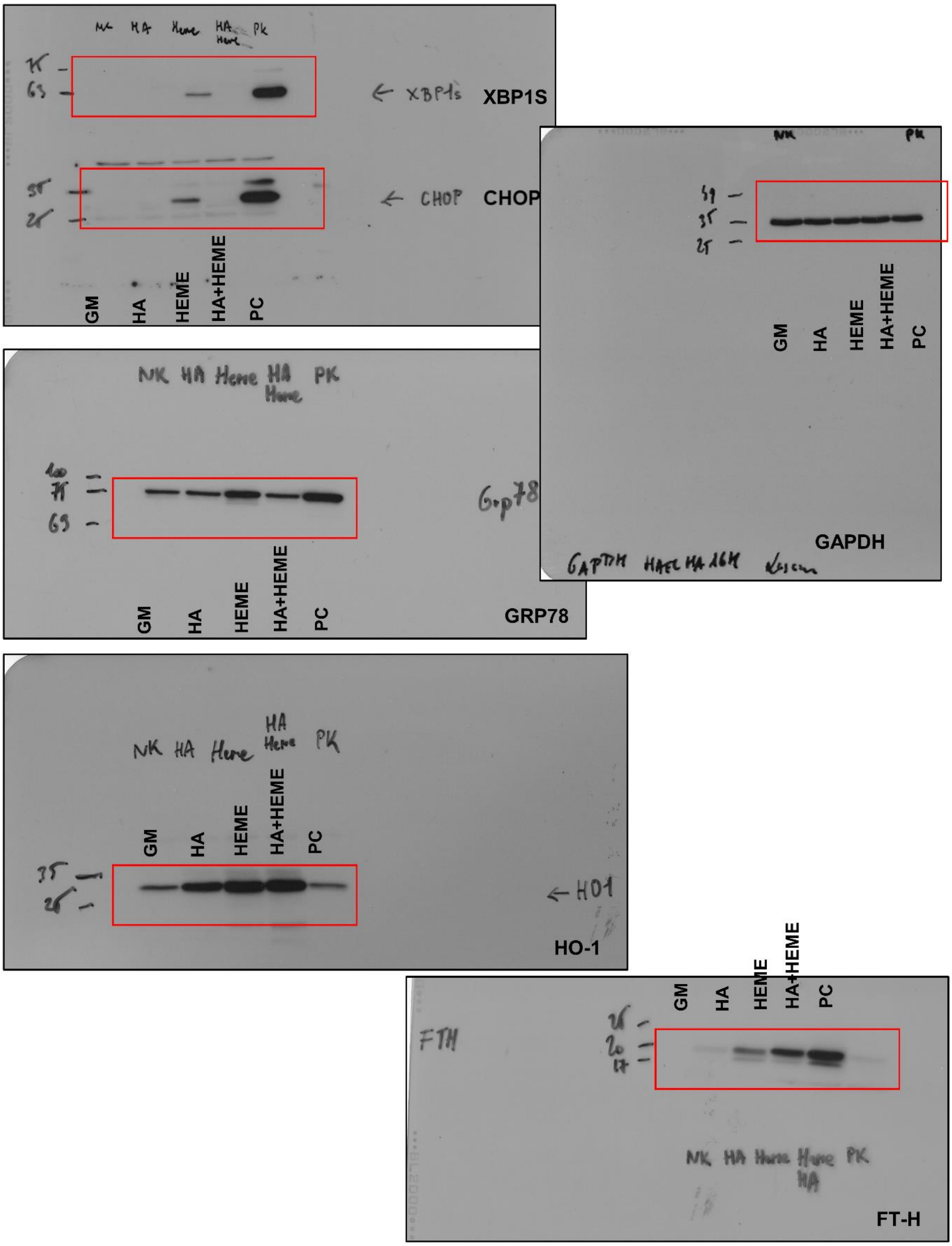

Figure 6D. Knocking down HO-1 but not ferritin heavy chain aggravates HIIER stress in EC cultures. FTH/ HO-1 silencing 3h experiment.

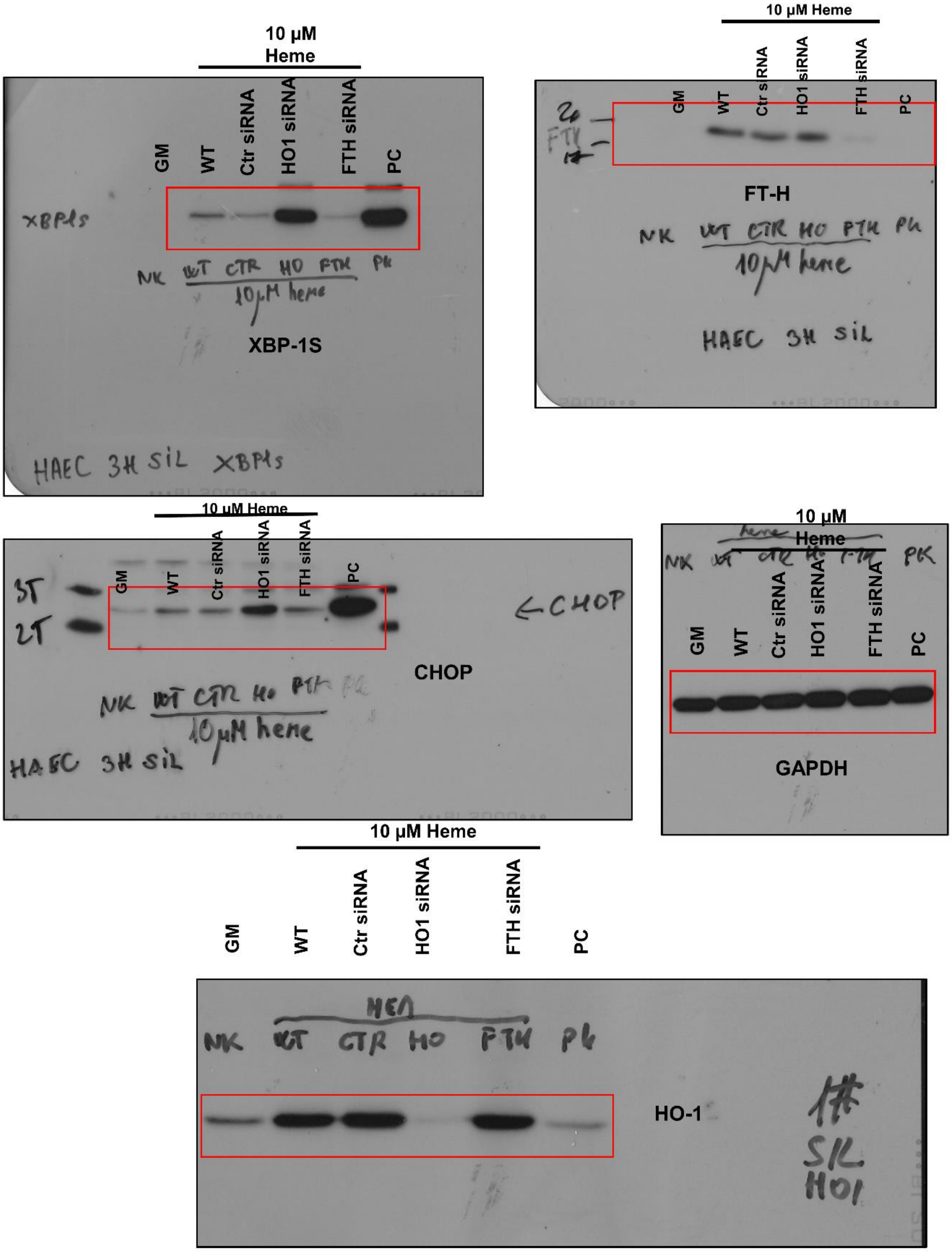

Figure 6H. Knocking down HO-1 but not ferritin heavy chain aggravates HIER stress in EC cultures. FTH/ HO-1 silencing 16 hr

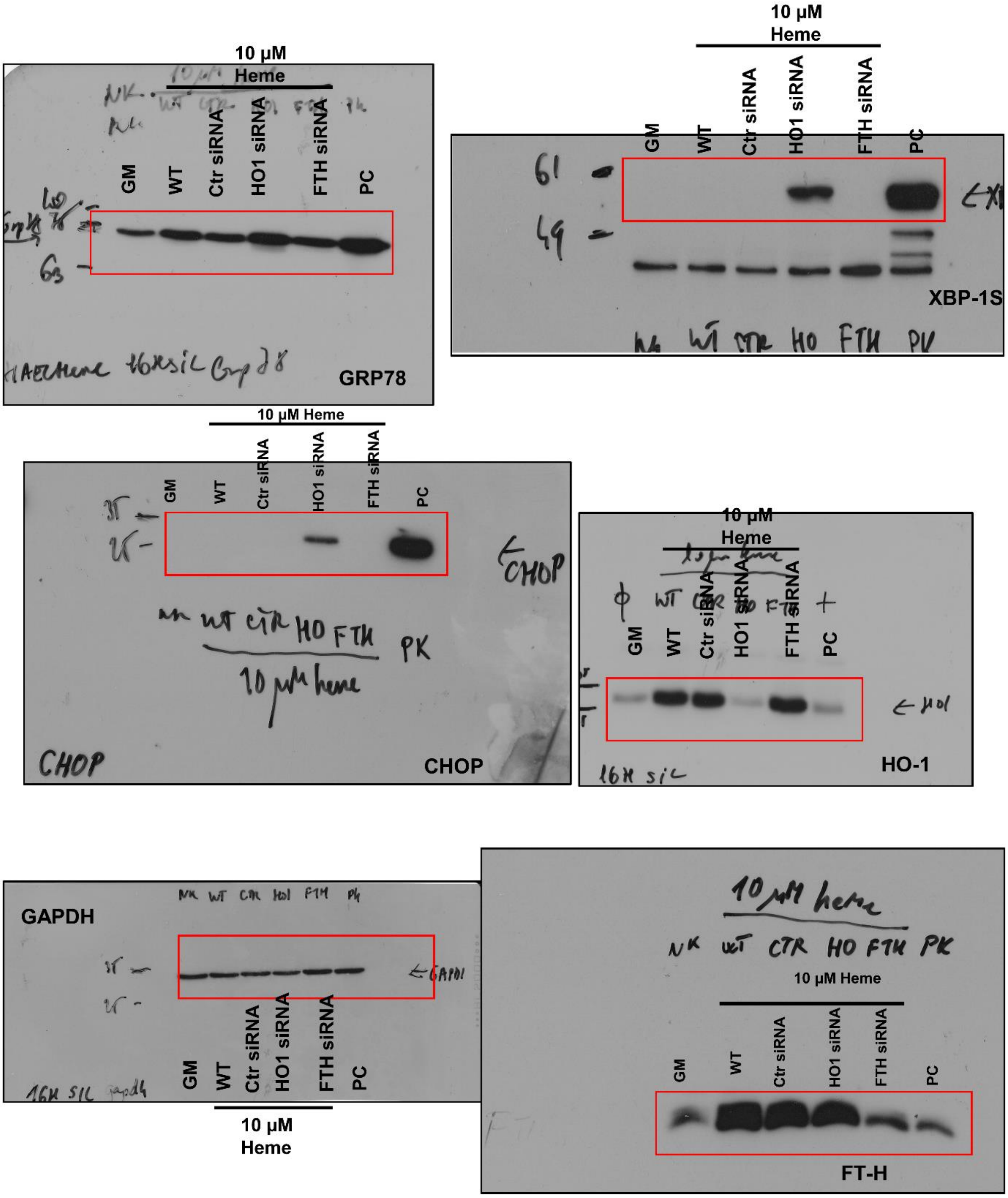

Figure 7D. Lack of protection by heme arginate against HIER stress in HO-1-silenced cells.

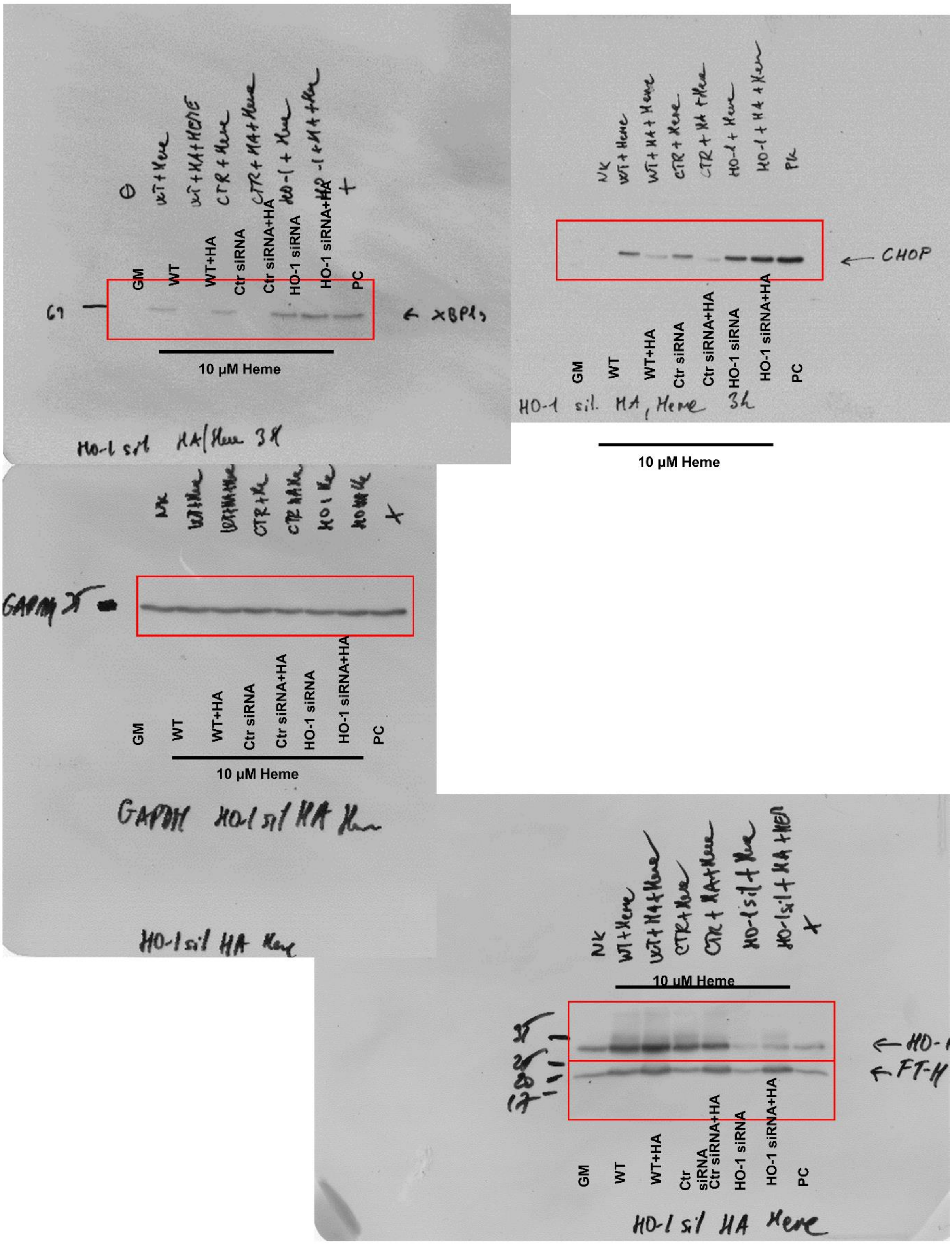

Figure 8D. Knocking down heme oxygenase-2 (HO-2) does not aggravate HIER stress.

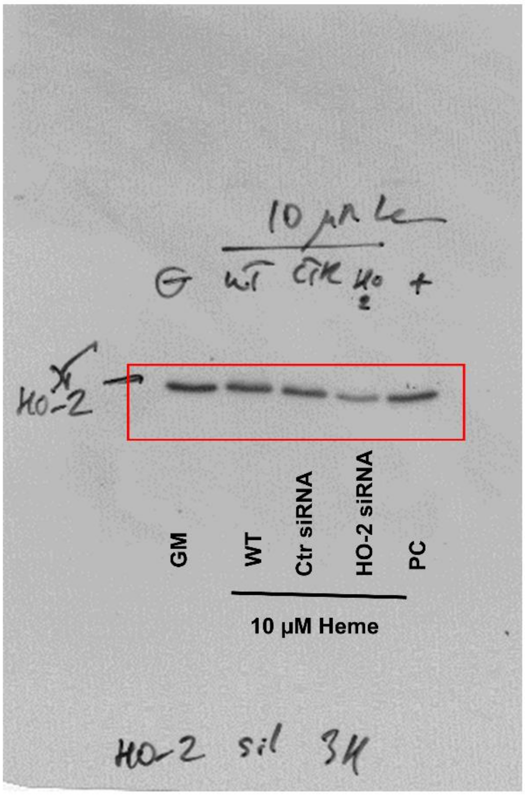

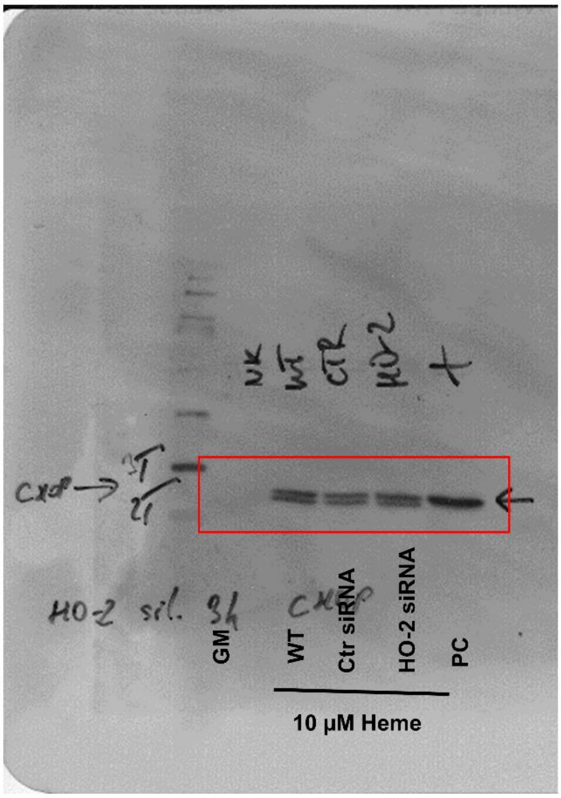

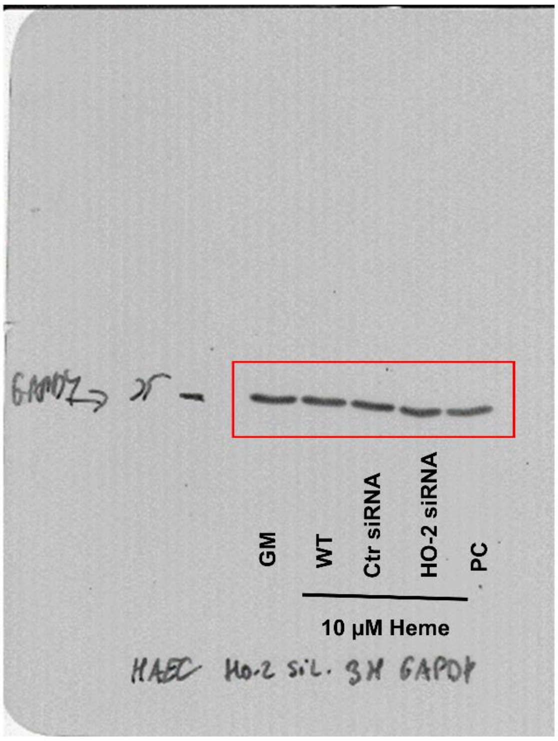

Figure 8G. Knocking down heme oxygenase-2 (HO-2) does not aggravate HIER stress.

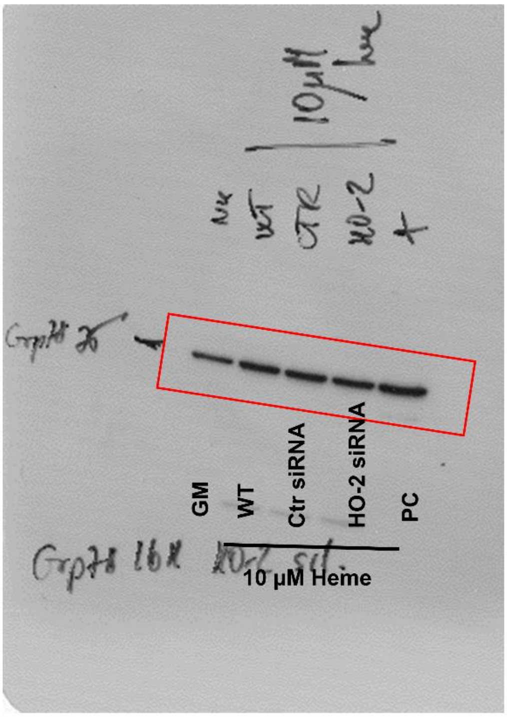

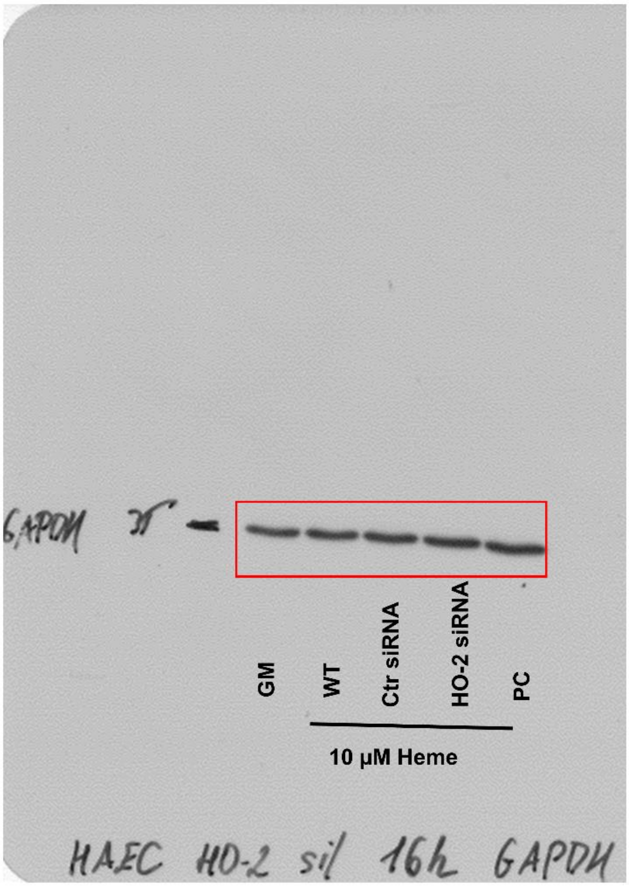

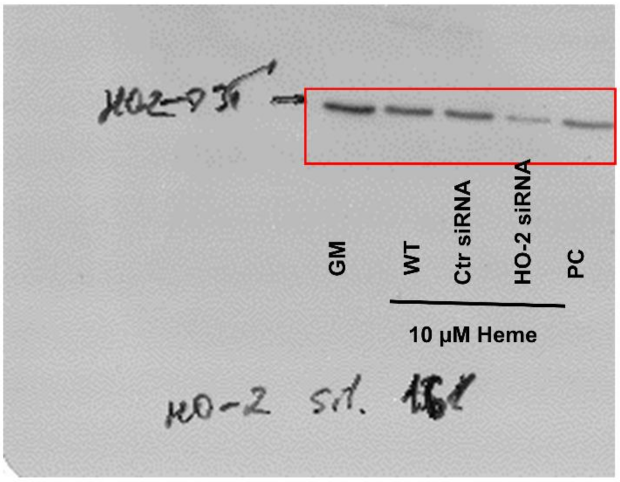

Figure 9D. Knocking down biliverdin reductase does not aggravate HIIER stress in EC cultures. BVRA/HO-1 silencing 3 h

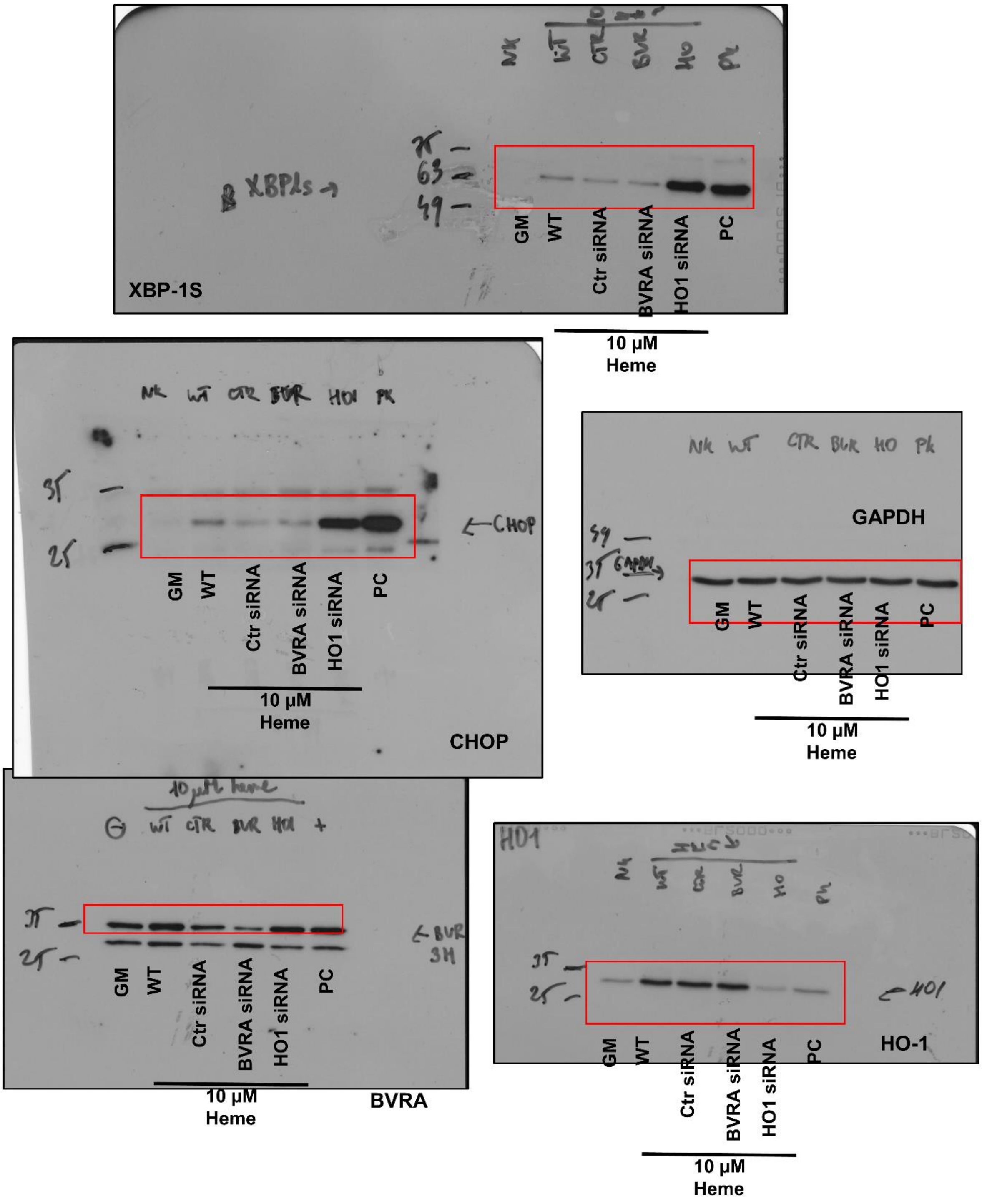

Figure 9H. Knocking down biliverdin reductase does not aggravate HIIER stress in EC cultures. BVR silencing+Heme arginate

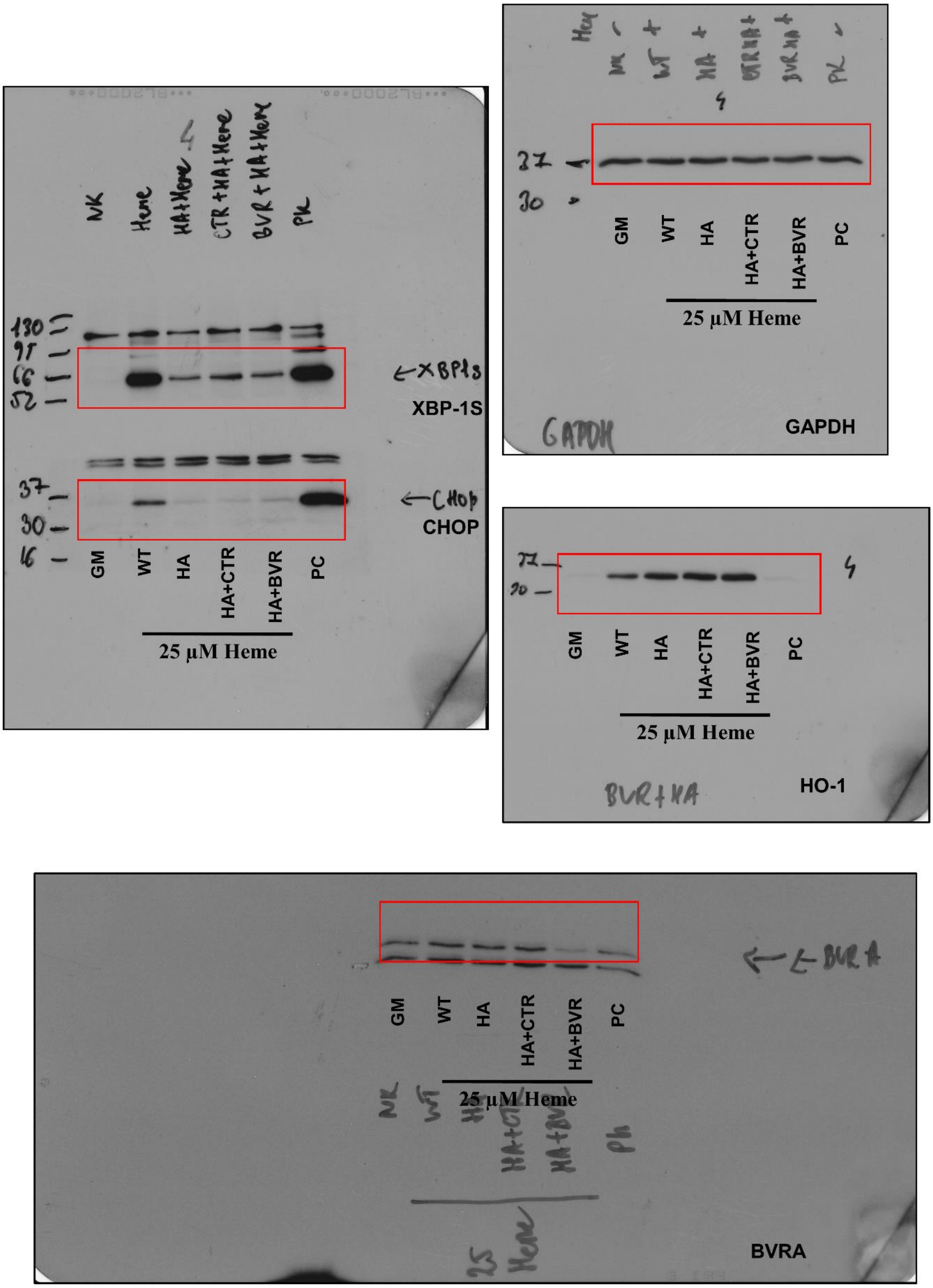

Figure 10D. Exogenous carbon monoxide but not bilirubin attenuates HIIER stress. BILIRUBIN

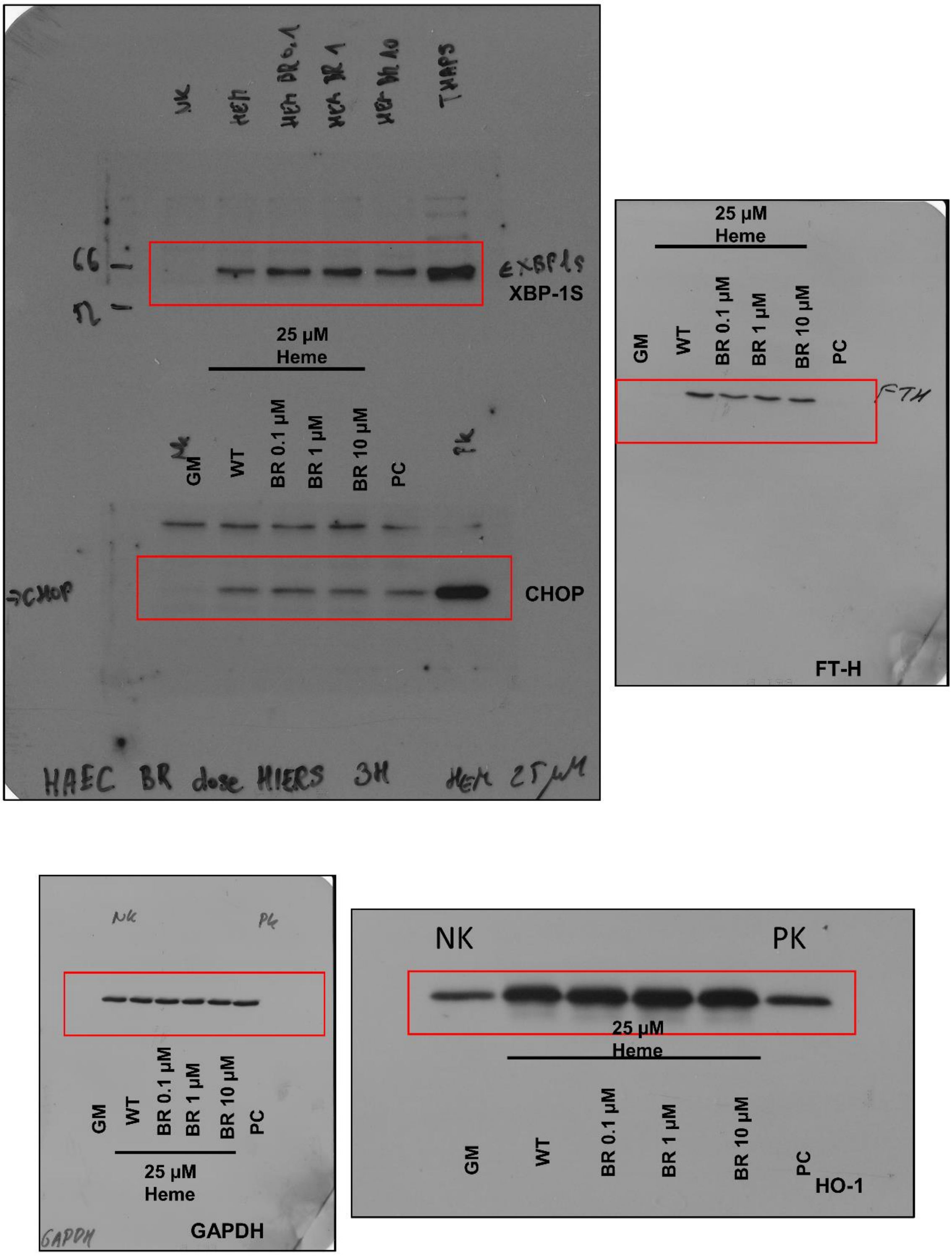

Figure 10H,I. Exogenous carbon monoxide but not bilirubin attenuates HIIER stress.

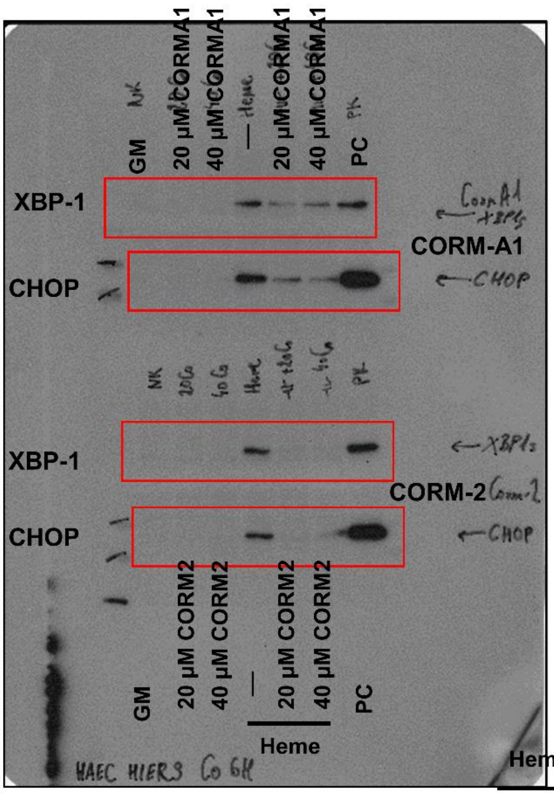

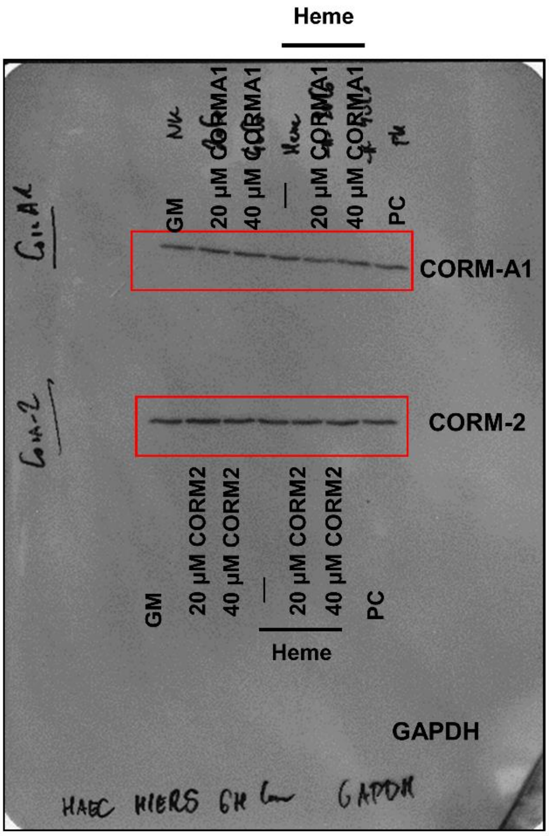

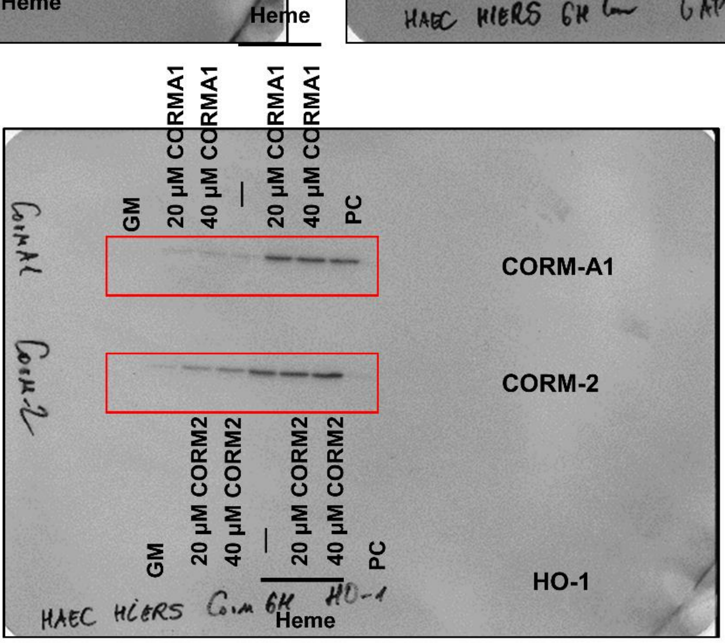

Supplementary figure 1. Heme does not induces ATF-5 in EC cultures.

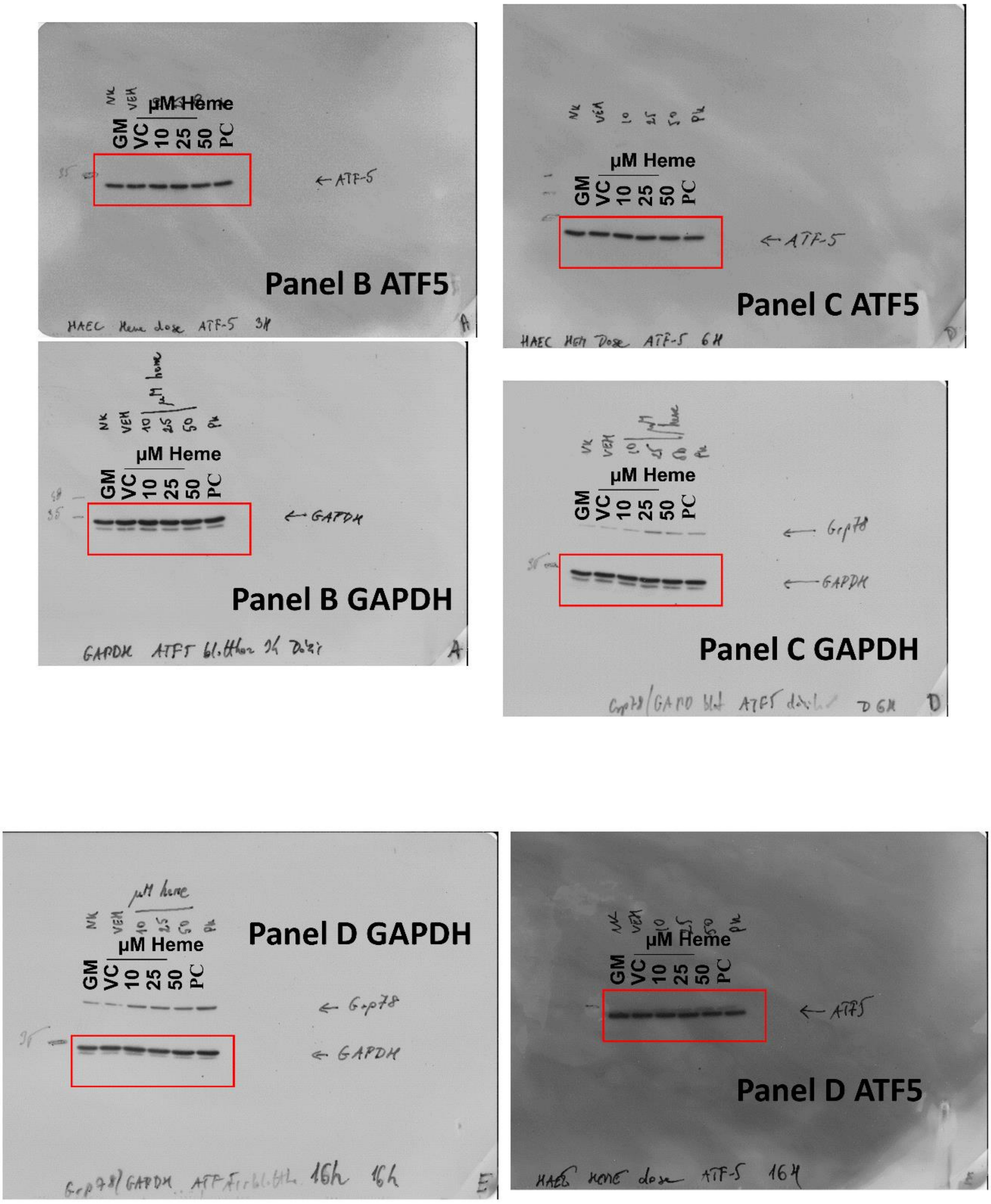

Supplementary figure 2. Heme induces ERN1 mRNA but not protein expression in EC cultures.

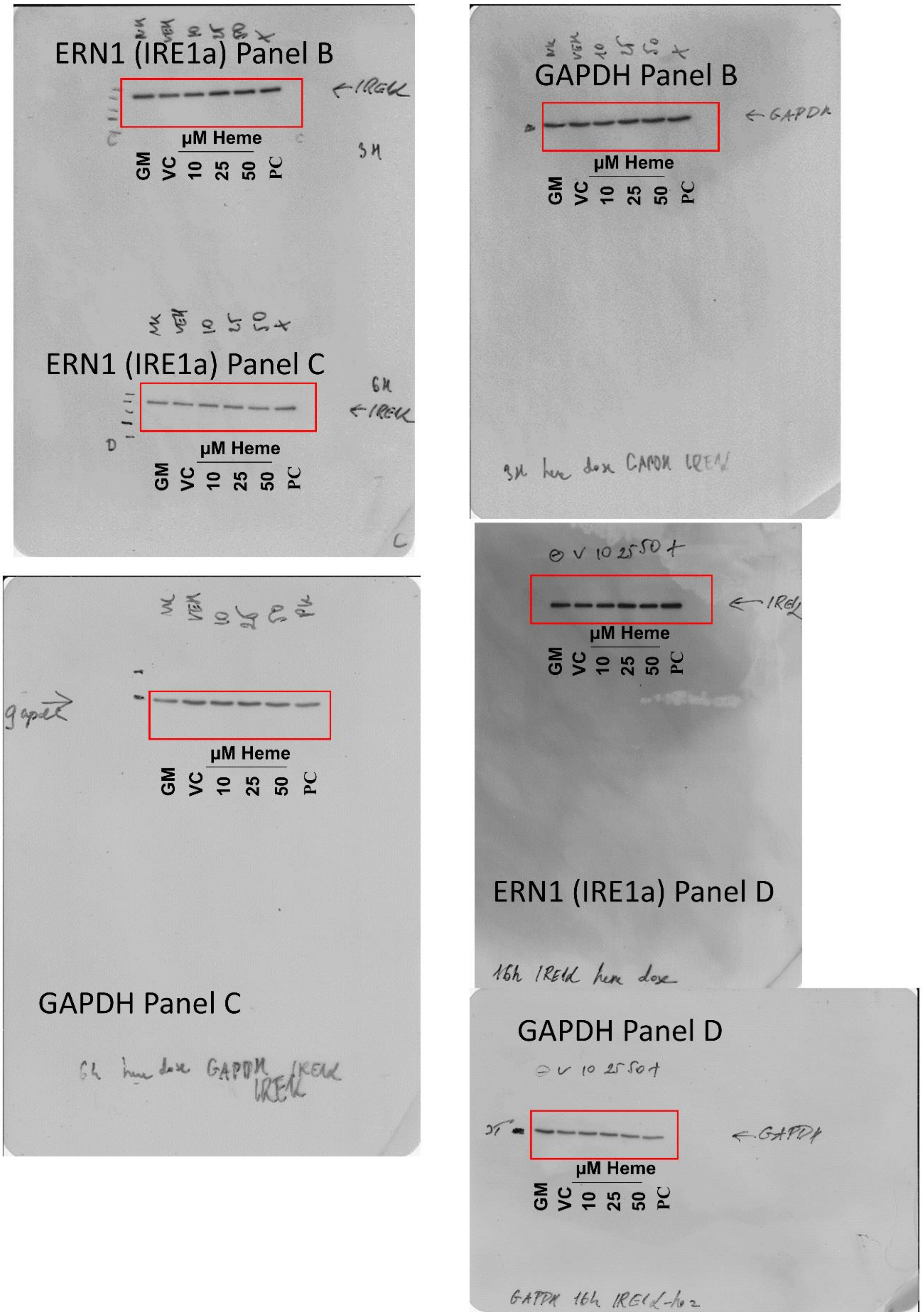

Supplementary figure 4D. Low-dose heme attenuates HIER stress in EC cultures.

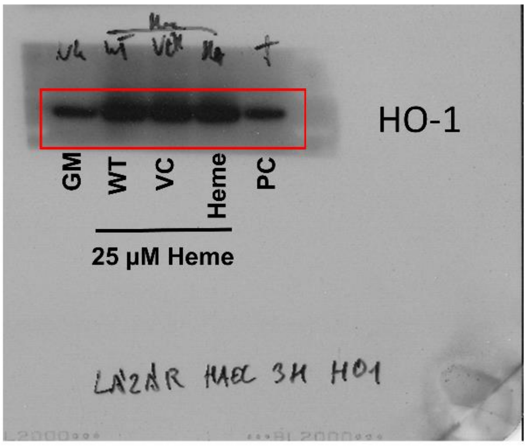

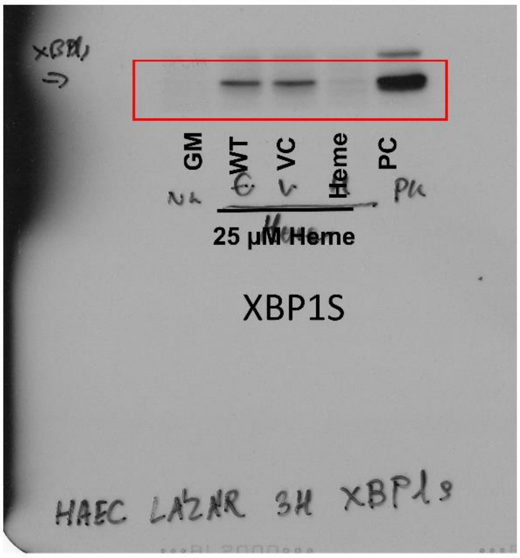

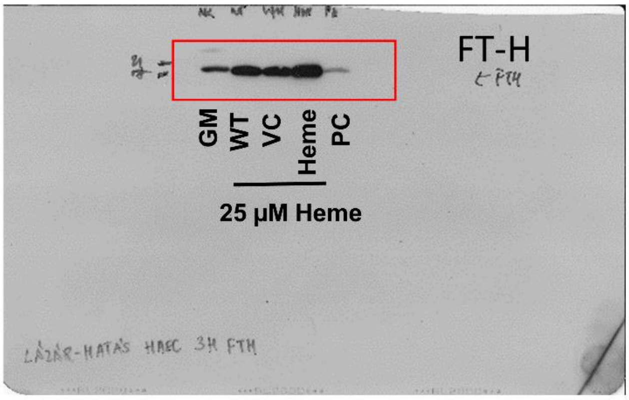

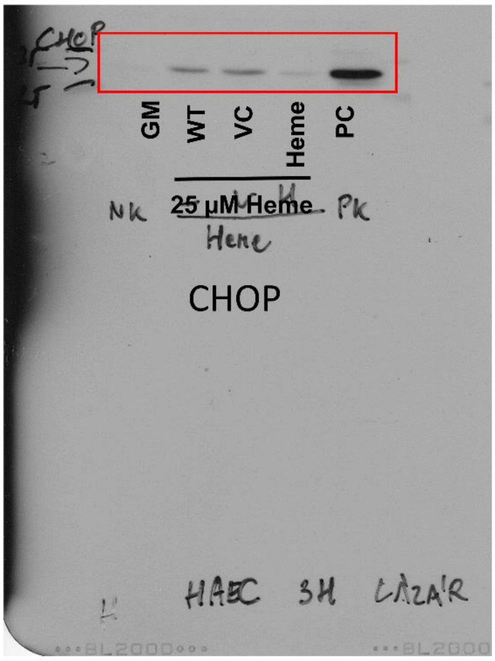

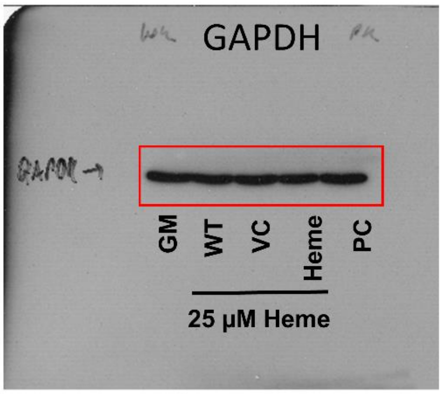

Supplementary figure 4H. Low-dose heme attenuates HIER stress in EC cultures.

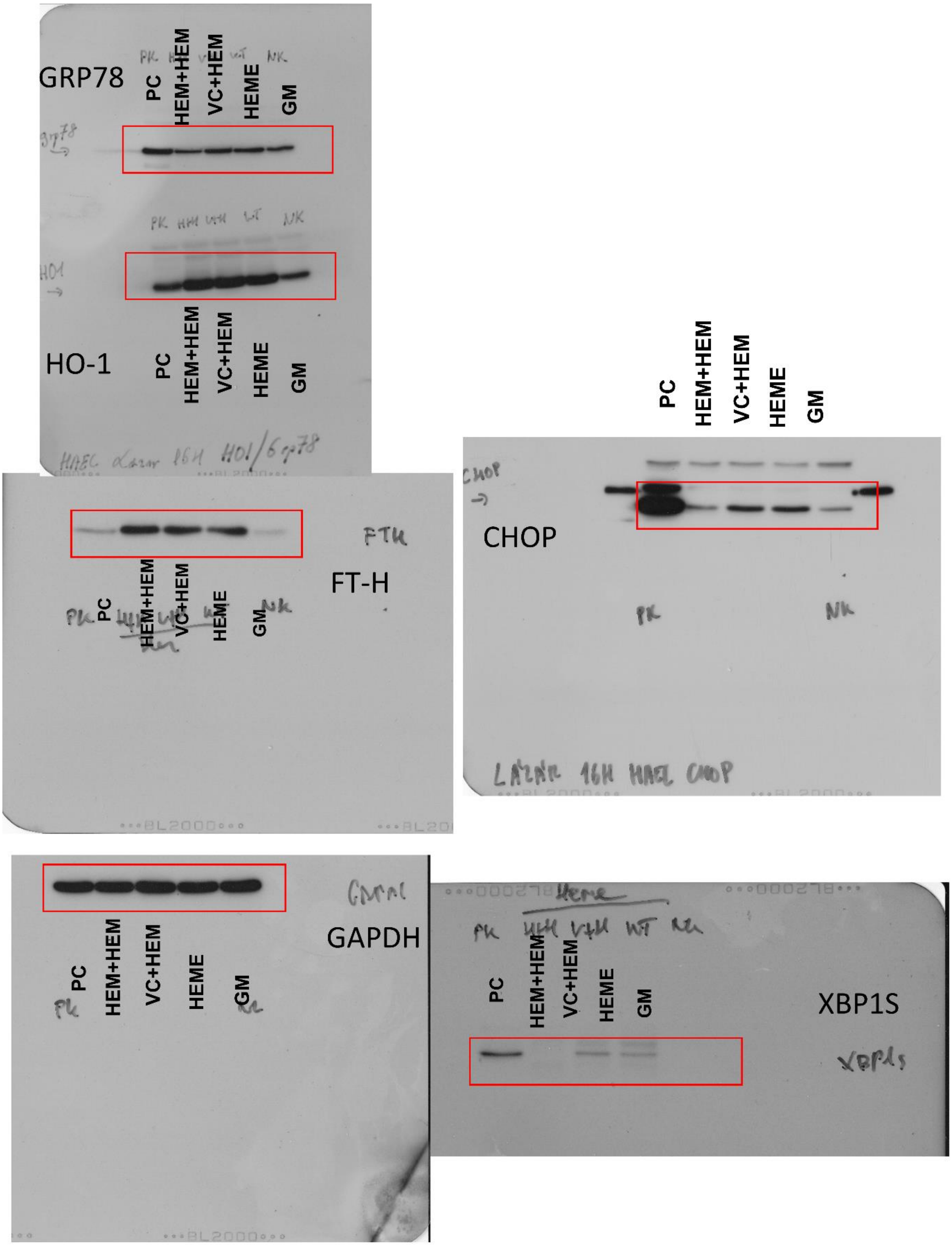

Supplement: Supplementary file 1 — Supplementary Figures. [file 41598_2021_89713_MOESM1_ESM.pdf]
